# Carfilzomib-specific proteasome β5/β2 inhibition drives cardiotoxicity via remodeling of protein homeostasis and the renin-angiotensin-system

**DOI:** 10.1016/j.isci.2025.113228

**Published:** 2025-07-29

**Authors:** Max Mendez-Lopez, Andrej Besse, Christian Zuppinger, Christian Perez-Shibayama, Cristina Gil-Cruz, Bogdan I. Florea, Angelina De Martin, Mechthild Lütge, Deborah Beckerova, Simon Klimovic, Xiang Zhou, Leo Rasche, Jan Pribyl, Vladimir Rotrekl, Burkhard Ludewig, Herman S. Overkleeft, Lenka Besse, Christoph Driessen

**Affiliations:** 1 Laboratory of Experimental Oncology, Division Oncology and Hematology, HOCH Health Ostschweiz, Cantonal Hospital St. Gallen, 9007 St. Gallen, Switzerland; 2 Department of Biology, Faculty of Medicine, Masaryk University, 62500 Brno, Czech Republic; 3 Department for Biomedical Research, Department of Cardiology, Bern University Hospital, 3008 Bern, Switzerland; 4 Institute of Immunobiology, HOCH Health Ostschweiz, Cantonal Hospital St. Gallen, 9007 St. Gallen, Switzerland; 5 Gorlaeus Building, Leiden Institute of Chemistry, 2333 Leiden, the Netherlands; 6 ICRC, St Anne’s University Hospital, 65691 Brno, Czech Republic; 7 Department of Biochemistry, Faculty of Science, Masaryk University, 62500 Brno, Czech Republic; 8 Department of Internal Medicine II, University Hospital of Würzburg, 97080 Würzburg, Germany; 9 Mildred Scheel Early Career Center, University Hospital of Würzburg, 97080 Würzburg, Germany; 10 CEITEC, Masaryk University, 62500 Brno, Czech Republic

**Keywords:** Pharmacology, Natural sciences, Biological sciences, Physiology

## Abstract

Compared to bortezomib treatment, multiple myeloma (MM) treatment with the proteasome inhibitor carfilzomib is associated with a higher incidence of cardiovascular adverse events. However, the mechanism underlying such cardiopathogenic side effects in MM patients remains elusive. Here, we show that carfilzomib-specific proteasome inhibition profoundly impairs cardiomyocyte contractility. Using an unbiased multiomics approach *in vitro* and *in vivo*, followed by *in vitro* validation, we elucidated carfilzomib-related changes in contractility proteins and cellular translation, retinol oxidative metabolism, and the angiotensin II derivative, angiotensin A. Subsequently, all-trans retinoic acid and angiotensin II type 1 receptor inhibitor prevented cardiomyocytes from experiencing carfilzomib-induced toxicity in human and murine *in vitro* and *in vivo* models through stabilization of protein and metabolic homeostasis. Our data reveal a mechanism underlying carfilzomib-induced cardiotoxicity that closely mirrors clinical observations and may open new avenues for management of such potentially lethal side effects in patients with MM.

## Highlights

• High-dose carfilzomib inhibits β2 proteasome subunit, reducing contractility and heart rate
• Contractility and heart rate impairment is associated with Aldh1a1 and Ang-A accumulation
• atRA co-treatment prevents carfilzomib-induced cardiotoxicity and reduces cardiac Ang-A

## Graphical abstract

**Figure:**
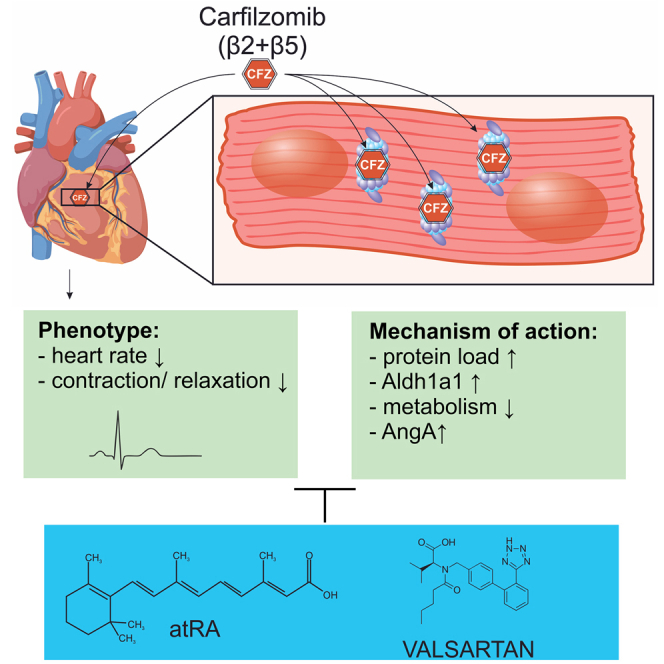

## Introduction

Multiple myeloma (MM) is a malignancy in which terminally differentiated plasma cells produce excessive amounts of monoclonal immunoglobulins.^1^ The ubiquitin-proteasome system (UPS) maintains an equilibrium between protein synthesis and degradation,^2^ and its pharmacological inhibition with proteasome inhibitors (PIs) causes proteotoxic cell death in MM cells.^3^ Currently, three PIs are clinically approved for the treatment of MM: the boronate-based inhibitors bortezomib (BTZ) and ixazomib (IXA), and the epoxyketone-based inhibitor carfilzomib (CFZ). The differential inhibition patterns of proteasome subunits β1, β2, and β5 by BTZ, IXA, and CFZ significantly influence their cytotoxic effects on MM cells.^4^^,^^5^ In contrast to BTZ and IXA, which target the proteasome subunits β5 and β1 at higher doses, CFZ is the only approved PI that co-targets β2 subunit, at higher doses, besides β1 co-targeting.^4^^,^^6^ This results in stronger functional proteasome inhibition, leading to different transcriptional and metabolic responses in MM cells, increased proteotoxic stress, and cytotoxicity.^4^^,^^7^ Clinically, CFZ treatment improves overall survival in patients with relapsed MM compared to BTZ. Moreover, high-dose CFZ, which co-targets the β2 subunit, restores therapeutic response in relapsed/refractory MM resistant to low-dose of the drug inhibiting only the β5 subunit.^6^^,^^8^ However, treatment of MM patients with CFZ above 56 mg/m^2^ is associated with significantly more cardiovascular adverse events (CVAEs), hypertension, and cardiotoxicity,^9^ presenting clinically as a reversible reduction in left ventricular ejection fraction in the absence of major cardiac structural changes.^10^^,^^11^

The molecular mechanism of CFZ-related cardiotoxicity remains incompletely understood. In murine models, prolonged CFZ administration decreased cardiac fractional shortening observed at the echocardiogram in young and aged mice, which was associated with increased myocardial oxidative stress, increased protein phosphatase 2A (PP2A) activity, and decreased AMP-activated protein kinase (AMPK) and Akt phosphorylation, decreased Mechanistic Target Of Rapamycin Complex 1 (mTORC1) activity, and autophagy,^12^^,^^13^ whereas others reported increased endoplasmic reticulum (ER) stress and autophagy following CFZ administration.^14^ Data from human induced pluripotent stem cell-derived cardiomyocytes (hiPSC-CMs) suggest major changes in ER stress induction, mitochondrial oxidative respiration and oxidative stress, Ca^2+^ transients, and cardiac contraction.^15^ These findings were later supported by observations of reduced oxygen consumption and both basal and maximal respiration following prolonged CFZ treatment, complemented by sarcomeric disarray due to decreased sarcomere protein turnover rate and the induction of heat shock proteins and ER stress.^16^ However, the degree of ER stress and unfolded protein response (UPR) induction is commonly observed both for BTZ and CFZ and does not fully explain increased incidence of CFZ-related heart failure.

In the *in vivo* models, cardiac contractility was improved by pre-treatment with anti-diabetic drugs metformin or empagliflozin, both of which induced autophagy and abrogated other CFZ-specific effects.^12^^,^^13^^,^^14^ Although it has been suggested that CFZ-related cardiotoxicity is associated with an off-target effect,^12^ heart failure has also been reported after treatment with BTZ and IXA^17^ and off-target activity contrasts with the superior specificity of CFZ.^18^ Thus, cardiotoxicity of CFZ may be in part related to the degree of functional proteasome inhibition. Due to their terminally differentiated nature, cardiomyocytes rely on the rapid turnover of sarcomere proteins for adequate contractility.^19^ Similarly, defects in the UPS are associated with heart failure during pressure overload,^20^ leading to cardiomyopathies.^21^^,^^22^ Thus, it is likely that interference with cardiomyocyte protein synthesis or degradation via proteasome inhibition leads to detrimental effects on cellular functions, such as cardiomyocyte contractility.

The early proteasome-related effects of CFZ on cardiomyocyte function, the direct comparison to BTZ, as well as the consequences of inhibiting different proteasome subunits in cardiomyocytes, remain unknown. We aimed to elucidate whether the functional consequences of β2 proteasome subunit co-inhibition provided by high-dose CFZ in the heart differ from those of β1 co-inhibition provided by high-dose BTZ during the early stage, before the onset of ER stress and the UPR induction. Our study provides evidence for a mechanism by which stronger functional proteasome inhibition by high-dose CFZ results in direct and rapid cardiomyocyte toxicity via a sharp block of protein turnover, and energy and metabolic changes in physiological models. These findings may have significant implications for MM patients, particularly when further validated in MM models.

## Results

### Carfilzomib-mediated β5 and β2 proteasome inhibition impairs cardiomyocyte contractility

**Figure 1:**
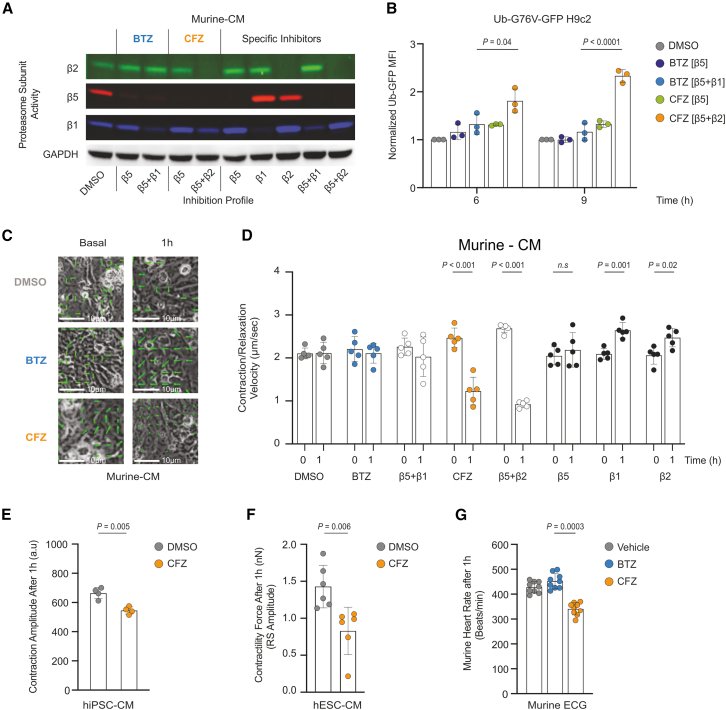
CFZ-mediated β5 and β2 proteasome inhibition results in impaired cardiomyocyte contractility (A) Activity-based probes showing proteasome subunit activities in beating Murine-CMs after 1 h exposure to BTZ (75 nM and 300 nM), CFZ (625 nM and 2,500 nM), or proteasome subunit-specific inhibitors and their combination (β5: NC005 = 10 μM; β1: NC001 = 5 μM; β2: LU102 = 3 μM). (B) Functional proteasome inhibition in Ub^−G76V−^GFP-positive H9c2 rat cardiomyoblasts. Cells were treated for 1 h with DMSO, BTZ, or CFZ at the doses inhibiting the β5 (BTZ = 75 nM; CFZ = 625 nM) β5 and β1 (BTZ = 300 nM) or β5 and β2 (CFZ = 2,500 nM) subunits; the data represent the mean ± SD (*n* = 3 independent experiments in duplicate). (C) Motion contractility vectors measured by automated image analysis of Murine-CM after treatment with DMSO, BTZ (300 nM), or CFZ (2,500 nM). The scale bar (5 mm) represents 10 μm. (D) Contraction velocity in Murine-CMs after 1 h treatment with DMSO, BTZ (300 nM), CFZ (2,500 nM) or proteasome subunit-specific inhibitors (β5: NC005 = 10 μM; β1: NC001 = 5 μM; β2: LU102 = 3 μM); the data represent the mean ± SD (*n* = 5 independent experiments in duplicate). (E) Contraction amplitude in hiPSC-CMs after 1 h treatment with CFZ (2,500 nM); the data represent the mean ± SD (*n* = 4 independent experiments in duplicates). (F) Contractility force of hESC-CMs after 1 h exposure to CFZ (2,500 nM) measured with atomic force microscopy; the data represent the mean ± SD (*n* = 6 independent experiments). (G) Murine heart rate measured with ECG 1 h after the injection with vehicle, BTZ (1 mg/kg), or CFZ (16 mg/kg); the data represent the mean ± SD (*n* = 9 mice). Statistical significance was tested with two-way ANOVA and Bonferroni correction for multiple comparisons. Two-tailed Student’s t test was performed when the data consisted of two groups. For all experiments, a p-value < 0.05 is considered statistically significant. See also Figures S1 and S2, and Videos S1, S2, S3, S4, S5, and S6. BTZ, bortezomib; CFZ, carfilzomib; DMSO, dimethyl sulfoxide; a.u, arbitrary units; MFI, median fluorescence intensity; nN, nanoNewtons; Murine-CMs, murine neonatal cardiomyocytes; hiPSC-CMs, human induced pluripotent stem cell-derived cardiomyocytes; hESC-CMs, human embryonic stem cell-derived cardiomyocytes.

BTZ inhibits β5 and β1 proteasome subunits at concentrations above 100 nM, while CFZ inhibits β5 and β2 at concentrations above 3,000 nM in MM cells after 1 h of treatment.^4^ In MM patients, the C_max_ for CFZ 30 min after infusion varies between 1,000 ng/mL (27 ng/m^2^ dose) and 3,000 ng/mL (70 mg/m^2^ dose),^23^ approximating in 2,500 nM *in vitro*. To compare the effects of BTZ and CFZ on proteasome subunit activity in cardiomyocytes, primary murine neonatal cardiomyocytes (Murine-CMs) and H9c2 cardiomyoblasts were exposed for 1 h to increasing concentrations of BTZ or CFZ or proteasome subunit-selective inhibitors for β1 (NC-001),^24^ β2 (LU-102),^25^ or β5 (NC-005),^26^ simulating PI exposure during intravenous clinical application.^27^^,^^28^ Lower concentrations of BTZ (75 nM) and CFZ (625 nM) led to selective inhibition of proteasome β5 activity, as expected (Figure 1A). CFZ resulted in additional full co-inhibition of β2 subunit activity and partial co-inhibition of β1 activity in cardiomyocytes at concentrations at 2,500 nM, whereas BTZ fully inhibited the β1 subunit at concentrations above 150 nM, but did not affect β2 activity, consistent with our data on MM cells and in MM patients^4^^,^^6^ (Figures 1A and S1A). The subunit-selective inhibitors provided near-complete inhibition of the activity of their respective targets β1, β2, β5, as expected (Figure 1A).

To address the functional effect of the differential proteasome subunit inhibition patterns on protein turnover in viable cardiomyocytes, we used H9c2 cells equipped with the ubiquitinated-GFP (Ub^−G76V−^GFP) construct.^29^ Only high-dose CFZ treatment at a β5+β2 co-inhibitory concentration (2,500 nM) resulted in an increased GFP signal, indicating a decrease in proteasomal protein degradation, in contrast to high-dose BTZ treatment at a β5+β1 co-inhibitory concentration (300 nM) (Figure 1B).

Next, we explored the effect of CFZ-type β5+β2 proteasome co-inhibition on cardiomyocyte contraction using isolated neonatal Murine-CMs.^30^ We quantified the contraction velocity of Murine-CMs before and after 1 h pulse treatment with BTZ, CFZ, or the subunit-selective PIs by video recording^31^ (Figure S1B, Videos S1, S2, S3, S4, S5, and S6). Treatment of Murine-CMs with either a combination of β5 and β2 selective inhibitors or with high-dose CFZ significantly reduced spontaneous contractile velocity (Figures 1C, 1D, and S1C–S1F). In contrast, cardiomyocyte contractile velocity was unaffected by the selective inhibition of the β2 proteasome subunit alone, in the absence of β2 co-inhibition achieved with high-dose BTZ, or by combination of subunit-selective β5 and β1 inhibitors (Figures S1C–S1F). Taken together, our data show that proteasome β5 and β2 subunit co-inhibition, achieved by high-dose CFZ or subunit-selective inhibitors, impairs cardiomyocyte contractile velocity.

Video S1. Analysis of motion vectors before DMSO treatment, related to Figure 1

Video S2. Analysis of motion vectors after 1 h treatment with DMSO, related to Figure 1

Video S3. Analysis of motion vectors before BTZ treatment, related to Figure 1

Video S4. Analysis of motion vectors after 1 h treatment with BTZ (β5 and β1 inhibition), related to Figure 1

Video S5. Analysis of motion vectors before CFZ treatment, related to Figure 1

Video S6. Analysis of motion vectors after 1 h treatment with CFZ (β5 and β2 inhibition), related to Figure 1

We confirmed the acute impairment of cardiomyocyte contractile velocity by high-dose CFZ in two *in vitro* human cardiac models. First, we measured contractile velocity in hiPSC-CMs as previously described^32^ (Figure S2A). The contraction amplitude of hiPSC-CMs rapidly decreased within 1 h of CFZ treatment and persisted reduced for 24 h, suggesting an impairment in contractility (Figures 1E, S2B, and S2C). Next, we assessed the direct effect of high-dose CFZ on the contractile force in human embryonic stem cell-derived cardiomyocyte (hESC-CM) clusters (embryonic bodies) by measuring the decline in the RS amplitude.^33^ CFZ caused cardiomyocyte embryonic bodies dysfunction by significantly reducing the contraction force after 1 h (Figures 1F and S2D). These data are consistent with acute, direct impairment of cardiomyocyte contractile velocity in Murine-CMs following high-dose CFZ treatment, inhibiting the β5+β2 proteasome subunits.

We used BALB/c mice to investigate the effect of CFZ on proteasome subunit activity in murine bone marrow and hearts 1 h after drug injections (Figure S2E). Consistent with the *in vitro* data, CFZ almost completely inhibited β5 proteasome activity and decreased β2 and β1 proteasome activity *in vivo* by > 50% at doses of 8–16 mg/kg. The 16 mg/kg resembles the 2,500 nM *in vitro* dose and a clinically achievable high dose CFZ. In contrast, treatment with BTZ at the established dose of 1 mg/kg^18^ did not inhibit the β2 proteasome subunit in the heart, but reduced β5 and β1 activity by > 90% (Figures S2F and S2G), consistent with the *in vitro* data. Next, we investigated the differential effects of BTZ- and CFZ-mediated proteasome inhibition on cardiomyocyte function *in vivo*. Murine heart rate and rhythm were monitored with an external electrocardiogram (ECG) under anesthesia 1 h after *i.v.* treatment with vehicle (Captisol), BTZ (1 mg/kg), or CFZ (16 mg/kg). A single application of CFZ at the dose co-inhibiting cardiac β5 and β2/β1 proteasome activity induced acute bradycardia with a mean heart rate (MHR) of 340.98 beats/minute, in contrast to the MHR of 452.6 beats/minute after BTZ-type β5 and β1 inhibition or vehicle (MHR of 428.05 beats/minute) (Figure 1G). We did not observe arrhythmias upon acute PI treatment. An interactive view of the electrocardiograms can be found at https://maxmendezl.shinyapps.io/ShinnyApp/.

Taken together, these data show that in contrast to BTZ, CFZ treatment affects more profoundly proteasome function via co-inhibition of the proteasome β2 subunit in cardiomyocytes, subsequently decreasing cardiomyocyte contractile velocity and force in murine and human cardiac models *in vitro*, and heart rate *in vivo*.

Carfilzomib treatment reduces quantity of contractility proteins and proteins involved in oxidative metabolism, and induces the retinol oxidative pathway to cope with contractility impairment *in vitro*.

To identify candidate dysregulated proteins related to acute contractile velocity dysfunction in isolated Murine-CMs, we performed quantitative proteome analysis using LC/MS-MS after 1 h of treatment with proteasome inhibitors at previously established doses (DMSO; BTZ = 300 nM; CFZ = 2,500 nM).

**Figure 2:**
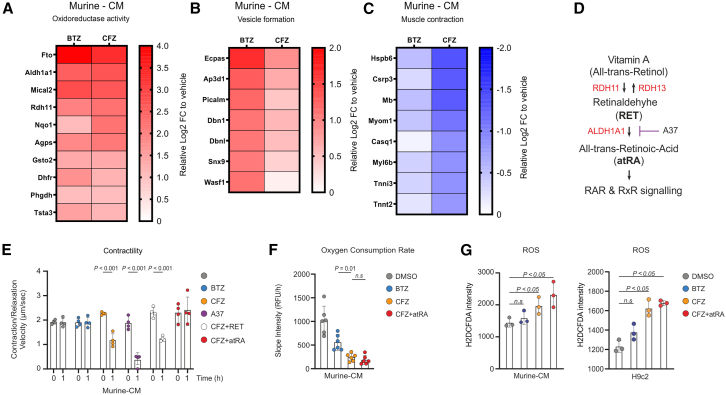
CFZ-induced cardiomyocyte contractile dysfunction is related to depletion of contractility proteins and changes in the retinol oxidative pathway (A–C) Heatmaps representing significantly deregulated proteins after 1 h of treatment with BTZ (300 nM), or CFZ (2,500 nM) and normalized to DMSO. (A) Proteins accumulated after both BTZ and CFZ treatment (Log2FC ≥ 1 for BTZ and CFZ; *p* < 0.01), clustering into oxidoreductase activity GO BP. (B) Proteins significantly accumulated after BTZ treatment (Log2FC for BTZ ≥ 1; Log2FC_BTZ - Log2FC_CFZ ≥ 0.5; *p* < 0.01), clustering into the endocytic vesicle compartment, based on GO CC. (C) Proteins depleted by CFZ, but not BTZ treatment (Log2FC for CFZ ≤ −0.8; Log2FC_BTZ - Log2FC_CFZ ≥ 0.5; *p* < 0.01), clustering into the muscle contraction process, based on GO BP, (*n =* 3 biological replicates). (D) Retinol pathway and proteins identified to be deregulated by BTZ and CFZ treatment. (E) Beating velocity of Murine-CMs after exposure to proteasome inhibitors (BTZ = 300 nM; CFZ = 2,500 nM) or after modulation of the retinol pathway (RET = 2 μM; A37 = 6 μM); the data represent the mean ± SD (*n* = 4 independent experiments in duplicate). (F) Oxidative phosphorylation analysis in Murine-CMs after 1 h of treatment with BTZ (300 nM), CFZ (2,500 nM), or CFZ (2,500 nM) and atRA (15 μM); the data represent the mean ± SD (*n* = 3 independent experiments in duplicate). (G) ROS measurement using H2DCFDA staining in Murine-CMs (on the left) and H9c2 cells (on the right) after 1 h of treatment with BTZ (300 nM), CFZ (2,500 nM) or CFZ (2,500 nM) and atRA (15 μM); the data represent the mean ± SD (*n* = 3 independent experiments). Statistical significance was tested with two-way ANOVA and Bonferroni correction. For all experiments, a p-value < 0.05 is considered statistically significant. See also Table S1 and Figure S3. Aldh1a1, aldehyde dehydrogenase 1A1; atRA, all-trans retinoic acid; BP, biological process; BTZ, bortezomib; CC, cellular component; CFZ, carfilzomib; DMSO, dimethyl sulfoxide; ECG, electrocardiography; FC, fold change; Murine-CMs, murine neonatal cardiomyocytes; GO, gene ontology; hiPSC-CMs, human induced pluripotent stem cell-derived cardiomyocytes; RDH11, retinol dehydrogenase 11; RDH13, retinol dehydrogenase 13; RET, retinaldehyde; RFU/h, relative fluorescence units per hour; ROS, reactive oxygen species.

Proteasome inhibition by both inhibitors led to the accumulation of multiple identical proteins (Table S1), mainly related to the regulation of oxidation-reduction processes and metabolism (Figure 2A). The changes related to metabolism of retinoic acid (Aldh1a1 and Rdh11) were slightly stronger for CFZ than for BTZ (Figure 2A). BTZ over CFZ treatment led to a more significant accumulation of proteins involved in actin binding and vesicle formation, such as Dbn1, DbnI, Ecpas, Picalm, Snx9, and Wasf1 (Figure 2B), whereas CFZ specifically increased the level of only a few nuclear/cytoplasmic proteins (Figure S3A), which are involved in multiple different processes including DNA damage-induced ubiquitination.^34^ Interestingly, both PI caused a rapid decrease in the levels of proteins related mainly to mitochondria and respiration (Figures S3B and S3C). Moreover, BTZ over CFZ more significantly decreased the levels of additional proteins localized in the mitochondrial matrix and membrane (Figures S3D and S3E), which are involved in oxidative phosphorylation (OXPHOS) and the tricarboxylic acid cycle, suggesting mitochondrial impairment and metabolic shutdown. On contrary, CFZ, but not BTZ, significantly reduced the quantity of proteins involved in myofibril assembly and muscle contractility (Figure 2C). Thus, *in vitro*, CFZ treatment specifically depleted the proteins involved in contractility, whereas it induced the accumulation of Aldh1a1, which was validated by immunoblotting (Figure S3F).

Aldh1a1 oxidizes retinol to all-trans retinoic acid (atRA; Figure 2D), which is subsequently involved in detoxification of acetaldehydes and lipid peroxides, all generated by oxidative stress—a known contributor to cardiotoxicity. Thus, its upregulation may represent a cellular defense mechanism against proteasome inhibitor-induced contractility impairment.^35^ Inhibition of Aldh1a1 enzymatic activity in Murine-CMs using the small molecule A37 led to a rapid abrogation of cardiomyocyte contractile velocity (Figure 2E) without apoptosis stimulation (Figure S3G). Notably, co-treatment with CFZ and atRA prevented contractile velocity impairment (Figure 2E), while co-incubation with CFZ and the Aldh1a1 enzyme substrate, retinaldehyde (RET), showed no rescue effect, further supporting the importance of RA metabolism in cardiomyocyte contractility.

To further dissect whether atRA rescues contractile velocity impairment *in vitro* by preventing CFZ-induced changes in OXPHOS, we assessed the effect of BTZ, CFZ, and atRA on the OXPHOS pathway by measuring the oxygen consumption rate (OCR). Both PIs caused a rapid decrease in OXPHOS, consistent with the impairment of mitochondria-related proteins in the *in vitro* model; however, CFZ-mediated proteasome inhibition led to stronger reduction of the OCR than BTZ-mediated inhibition (Figure 2F). Nonetheless, co-treatment with CFZ and atRA did not prevent the CFZ-related decrease in OCR in Murine-CMs (Figure 2F). Likewise, CFZ, but not BTZ, significantly induced reactive oxygen species (ROS) formation in Murine-CMs and H9c2 cardiomyoblasts, which was not prevented by atRA (Figure 2G).

These data suggest that CFZ-related contractility impairment is significantly associated with CFZ-induced alterations in the turnover of contractility proteins, as well as changes in the retinol oxidative pathway. Specifically, the end-product, atRA, alleviates the contractile velocity changes through an alternative mechanism.

### Carfilzomib treatment *in vivo* deregulates proteins important for protein translation and cardiomyocyte metabolism, which is prevented by atRA

**Figure 3:**
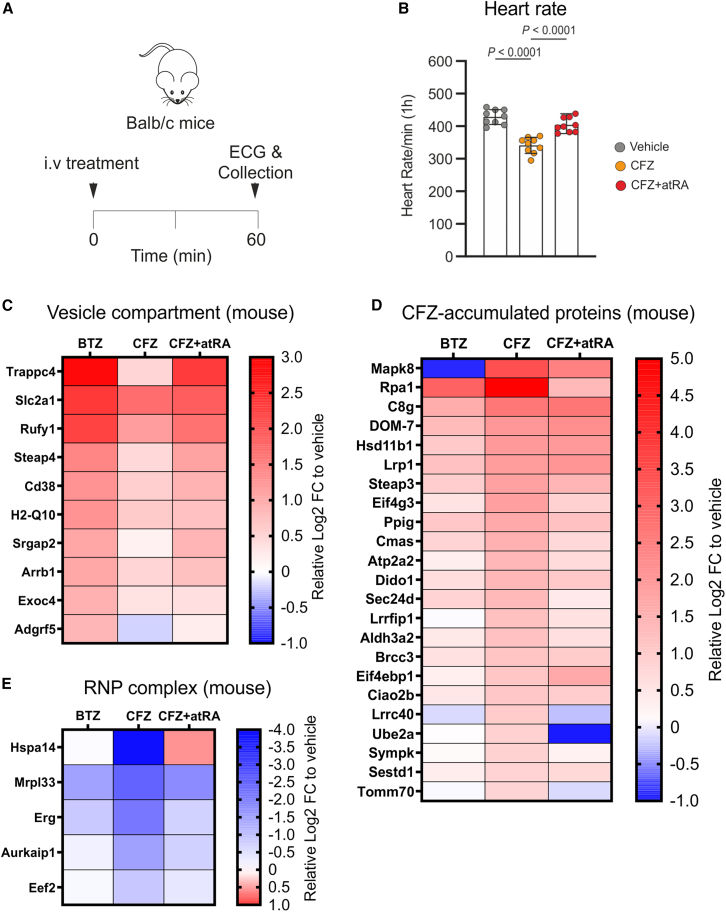
CFZ treatment impairs key regulators of cardiomyocyte contractility and metabolism, which can be restored by atRA co-treatment (A) Experimental design of the work flow in mice used to evaluate ECG and collect hearts for proteomic, metabolomic, and scRNA-seq analysis. (B) Murine heart rate, measured by ECG at the end of 1 h of treatment with vehicle, CFZ (16 mg/kg) or CFZ + atRA (16 mg/kg and 1 mg/kg), the data are presented as the mean ± SD (*n* = 9 mice per group). A p-value < 0.05 is considered statistically significant. (C–E) Heatmaps representing deregulated proteins in murine hearts after 1 h of treatment with BTZ (1 mg/kg) vs. vehicle, CFZ (16 mg/kg) vs. vehicle, and CFZ + atRA (16 mg/kg and 1 mg/kg) vs. vehicle. (C) Proteins accumulated specifically after BTZ treatment, but not after CFZ treatment (Log2FC for BTZ ≥ 0.8; Log2FC_BTZ - Log2FC_CFZ ≥ 0.5; *p* < 0.05), clustering to the vesicle compartment, based on GO CC. (D) Proteins significantly accumulated after CFZ treatment, but not after BTZ treatment (Log2FC for CFZ vs. vehicle ≥ 0.8; Log2FC_CFZ vs. Vehicle - Log2FC_BTZ vs. Vehicle ≥ 0.5; *P* (CFZ vs. Vehicle) < 0.05). (E) Proteins significantly reduced after CFZ treatment, but not after BTZ treatment (Log2FC for CFZ vs. Vehicle ≤ −0.8; Log2FC_BTZ vs. Vehicle - Log2FC_CFZ vs. Vehicle ≥ 0.5; *P* (CFZ vs. Vehicle) < 0.05) clustering to the ribonucleoprotein (RNP) complex, based on GO CC (*n =* 3 mice per group). Statistical significance was tested with a t test. See also Tables S2 and S3; Figure S4. atRA, all-trans retinoic acid; BTZ, bortezomib; CC, cellular compartment; CFZ, carfilzomib; ECG, electrocardiography; FC, fold change; GO, gene ontology.

Following the *in vitro* data, we investigated to what extent atRA ameliorates the effects of CFZ on heart rate and what are the global proteomic changes associated with CFZ-related toxicity *in vivo*. Mice were treated with vehicle (Captisol), BTZ, CFZ, or CFZ+atRA for 1 h, followed by ECG monitoring, heart extraction, and LC-MS/MS analysis of the global heart proteome (Figure 3A). The interactive view of the ECGs can be accessed via the link provided in the data and code availability section. Co-treatment of mice with CFZ and atRA significantly improved CFZ-induced bradycardia (Figure 3B). Importantly, atRA alone had no influence on heart rate (Figure S4A), but it partly prevented the β2 proteasome inhibition caused by CFZ (Figures S4B and S4C).

Proteomic analysis revealed that both CFZ and BTZ administration causes significant accumulation of multiple identical proteins involved in the proteasome network and myofibril organization (Figure S4D and Table S2). Specifically, consistent with the *in vitro* data, both drugs induced Aldh1a1 accumulation (Figure S4D). Moreover, both drugs induced the accumulation of protein phosphatase 6 regulatory subunit 3 (Ppp6r3/Saps3), regulating cellular metabolism via AMPK inactivation,^36^ previously reported as a cause of acute cardiac dysfunction by CFZ.^12^ BTZ treatment specifically induced the accumulation of multiple proteins (Figure S4E and Table S2) involved in formation of vesicles (Figure 3C), consistent with the *in vitro* data. In contrast, among proteins specifically accumulated after CFZ treatment (Figure 3D) were stress-related Jnk1 (Mapk8), DNA damage-related Rpa1 and Ube2a, translation initiation (eIF4g3) and repression (eIF4ebp1) factors, and contractility proteins, such as Serca2/Atp2a2 and Lrp1.

*In vivo*, both PI significantly reduced the levels of several proteins related to the ER compartment (Figure S4F), but not the mitochondria, as the *in vitro* data suggested. The proteins specifically reduced by BTZ treatment did not cluster into any significant processes or compartments (Figure S4G and Table S2). On contrary, proteins that were specifically decreased by CFZ treatment (Figure S4H) significantly clustered in ribonucleoprotein complex formation and translation (Figure 3E). Thus, CFZ, but not BTZ, significantly affected proteins involved in protein translation, which likely impaired the protein turnover.

AtRA co-treatment did not prevent the quantitative changes in proteins commonly deregulated (accumulated or reduced) after treatment with BTZ and CFZ, suggesting that it does not prevent contractility impairment solely by lowering the protein burden on the proteasome (Figure S4D and Table S3). However, it partly reduced accumulation and depletion of proteins significantly affected specifically by CFZ treatment (Figures 3D and 3E). Co-treatment with atRA significantly lowered the CFZ-mediated effect on Serca2 and on the decrease of several proteins involved in translation (Hspa14, Erg, and Aurkaip1, Figure 3E). Moreover, CFZ and atRA induced “BTZ-like” changes in the proteins that clustered to vesicle formation (Figure 3C), suggesting autophagy induction, previously observed for atRA.^37^ This implies that autophagy induction may play an important role in cardio-protection, as has been already proposed.^12^^,^^14^

Based on these data, we hypothesize that atRA reduces CFZ-induced contractility impairment *in vivo* via at least two mechanisms: (1) by reducing the extent of proteasome inhibition, provided by proteasome β2 co-inhibition, and (2) by alleviating translation impairment, which is associated with the induction of vesicle formation and autophagy, thereby supporting normal cardiac function and metabolism.

### Carfilzomib treatment induces a cardiomyocyte-specific transcriptional response that is alleviated by atRA

**Figure 4:**
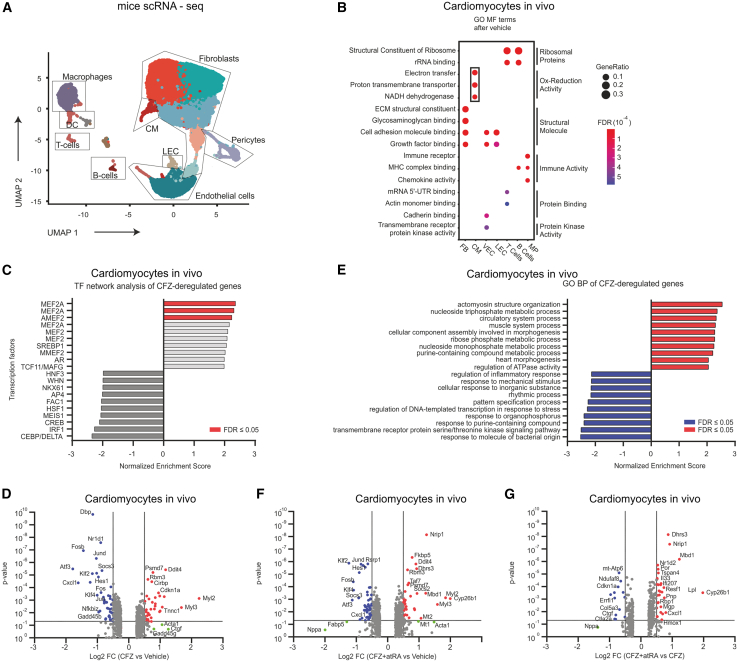
CFZ treatment causes cardiomyocyte-specific dysregulation in the transcriptional network related to cardiomyopathy-related remodeling (A) UMAP plot visualization of the cellular subtypes identified in murine hearts after acute proteasome inhibition with CFZ vs. vehicle (16 mg/kg, *n* = 2 mice per group). (B) Dotplot heatmap showing GO MF terms enriched in cluster marker genes after treatment with vehicle. Hypergeometric test for overrepresentation; Benjamini-Hochberg correction. (C) GSEA followed by TF network analysis of the differentially expressed genes in cardiomyocytes following CFZ (16 mg/kg) treatment and normalized to vehicle treatment. Significantly enriched TF networks are depicted in red. (D) Volcano plots representing genes with deregulated expression in the murine cardiomyocytes following CFZ (16 mg/kg) *i.v.* injection and normalized to vehicle treatment. Red represents genes with significantly increased expression (Log2 FC > 0.5, *p* < 0.05), blue represents genes with significantly decreased expression (Log2 FC < −0.5, *p* < 0.05), and green represents genes with a high but nonsignificant fold change. (E) GSEA followed by GO BP analysis of the differentially expressed genes in the cardiomyocytes following CFZ (16 mg/kg) *i.v.* injection and normalized to vehicle treatment. Red represents significantly enriched BP, blue represents significantly decreased BP. (F and G) Volcano plots representing genes with deregulated expression in murine cardiomyocytes following CFZ + atRA (16 mg/kg and 1 mg/kg, respectively) treatment and normalized to vehicle treatment (F) and CFZ + atRA treatment and normalized to CFZ treatment (G). Red represents genes with significantly increased expression (Log2 FC > 0.5, *p* < 0.05), blue represents genes with significantly decreased expression (Log2 FC < −0.5, *p* < 0.05), and green represents genes with a high but nonsignificant fold change. See also Tables S4, S5–S7, S8, and S9, and Figures S5A–S5D. atRA, all-trans retinoic acid; BP, biological process; CFZ, carfilzomib; CM, cardiomyocytes; GO, gene ontology; GSEA, gene set enrichment analysis; MF, molecular function; TF, transcription factor; UMAP, uniform manifold approximation and projection.

To specifically dissect the transcriptional landscape of CFZ-treated cardiomyocytes *in vivo* and the effect of atRA co-treatment on transcriptional changes as a consequence of CFZ-mediated repression of protein turnover, we analyzed the transcriptome of murine hearts at a single cell resolution using single-cell RNA sequencing (scRNA-seq) after 1 h treatment with vehicle, CFZ or CFZ + atRA (as depicted in Figure 3A). Unsupervised clustering revealed 13 distinct cell subsets present in the heart (Figure 4A). Computation of cluster-specific genes revealed transcriptional signatures consistent with fibroblasts (*Col1a1*, *Col1a2*, and *Tcf21*), vascular endothelial cells (*Cd36*, *Cdh5*, and *Pecam1*), macrophages (*Cd74*, *Cd14*, and *Cx3cr1*), pericytes (*Acta2*, *Mcam*, and *Itga7*), B cells (*CD79a* and *Ighd*), T cells (*Cd3e* and *Tcrg-C1*), lymphatic endothelial cells (*Prox1* and *Flt4*), and dendritic cells (*Itgax*) (Figure S5A and Tables S4 and S5). Cardiomyocytes were identified with the cardiac muscle troponins signature (*Tnni3*, *Tnnt2*, and *Tnnc1*; Figure S5A). Gene ontology (GO) analysis of cardiomyocytes vs. all other cell populations revealed enrichment in genes related to electron transfer, proton transport, and NADH dehydrogenase activity (Figure 4B), consistent with higher demands of cardiomyocytes for oxidative metabolism under normal conditions, in contrast to other cell populations.

Gene set enrichment analysis (GSEA) of CFZ vs. vehicle-deregulated genes in cardiomyocytes revealed that the upregulated genes are enriched with motifs for transcription factor binding, with Mef2a showing the highest enrichment score (Figure 4C and Table S6) Specifically, the expression of myosins (*Myl2* and *Myl3*), actins (*Acta1*, and*, Actc1*), tropomyosins (*Tpm1*), and troponins (*Tnnc1*) (Figure 4D and Table S7) was induced. Likewise, GSEA followed by KEGG pathway analysis confirmed that CFZ-induced genes are involved in actinomyosin structure organization and heart morphogenesis (Figure 4E and Table S8). Thus, the induced expression of these genes may compensate for the reduced turnover of structural proteins identified after CFZ treatment. Notably, CFZ vs. vehicle induced the expression of proteasome-related genes, consistent with Nrf1-mediated proteasome recovery after proteasome inhibition (Figure S5D).^38^

In contrast, GSEA of cardiomyocyte-specific genes downregulated by CFZ treatment suggested the involvement of cAMP response element-binding protein (Creb), which regulates the expression of *Atf3* and genes related to the AP-1 complex (*Fos*, *Fosb*, *Jun*, and *Jund*), all of which were consistently downregulated in our data (Figure 4D and Table S7). Moreover, the downregulated genes (*Gadd45b*, *Dbp*, and *Nr1d1)* are involved in other processes, e.g., response to ATP metabolites (such as organophosphorus) and in cellular rhythmicity (e.g., circadian rhythm genes regulating cellular metabolism; Figure 4E and Table S8).

GSEA followed by GO classification of the CFZ-induced genes in other cell populations within the heart, including endothelial cells, pericytes, fibroblasts, and macrophages, revealed a common feature: significant downregulation of genes involved in the response to ATP metabolites, as well as genes associated with cellular rhythmicity (Tables S7 and S8). Thus, CFZ-mediated proteasome inhibition induces acute and complex transcriptomic changes across multiple cell types in the heart. In cardiomyocytes, these changes involve disturbances in cellular remodeling and metabolic and energy homeostasis, closely resembling the changes seen due to Ca^2+^ handling impairment in cardiomyopathies.^39^ These data support our hypothesis that the primary cause of CFZ-induced cardiotoxicity lies within cardiomyocytes, rather than in vascular or endothelial niches.

Co-treatment with CFZ and atRA slightly ameliorated CFZ-induced transcriptomic changes but did not prevent their occurrence as the induction of myosins (Figures 4F and S5B), downregulation of stress-related genes (Figure S5C), or induction of proteasome-related genes was still prominent (Figure S5D). However, in contrast to CFZ monotherapy, atRA co-treatment induced the expression of several known atRA-regulated genes^40^^,^^41^ (e.g., *Cyp26b1*, *Dhrs3*, *Nrip1*, and *Dbp*) in multiple cell types. These genes are involved in the metabolism of atRA and lipids (Figure 4G and Table S9). Specifically, in cardiomyocytes, atRA co-treatment alleviated the CFZ-induced decrease in *Atf3*, AP-1 complex genes, *Nr1d1*, *Nr1d2*, and *Dbp* (Table S9). The data suggest that atRA does not counteract the CFZ-induced transcriptional changes in cardiomyocytes but rather improves cellular protein homeostasis and metabolic status.

### Carfilzomib impairs Mef2a and c-AMP-responsive signaling in cardiomyocytes, which is related to rapid functional proteasome inhibition, but not to UPR induction

**Figure 5:**
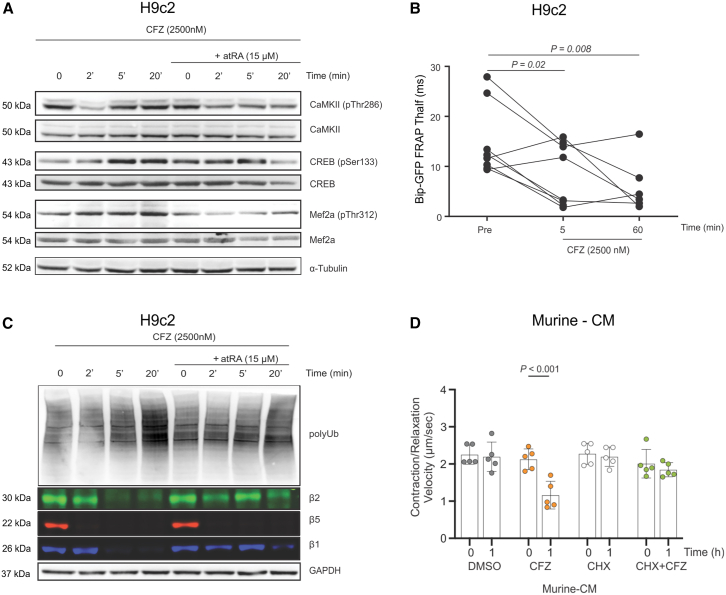
CFZ-mediated proteasome inhibition affects Mef2 and Creb signalinging and poly-Ub-protein accumulation, which is prevented by atRA (A) Induction of transcription factor signaling in H9c2 cardiomyoblasts after treatment with CFZ (2,500 nM) or CFZ + atRA (2,500 nM and 15 μM, respectively). Western blot analysis was performed at the indicated time points using the indicated concentrations of the drugs. Representative images of three independent experiments are shown. (B) Half-life of BiP-GFP recovery after photobleaching in H9c2 cells before CFZ (2,500 nM) treatment and 5 and 60 min after CFZ treatment. Statistical significance was tested using a t test between pre- and post-treatment time points. A p-value < 0.05 is considered statistically significant. (C) Accumulation of polyubiquitinated protein and inhibition of individual proteasome subunits in H9c2 cardiomyoblasts after the treatment with CFZ (2,500 nM) or CFZ + atRA (2,500 nM and 15 μM, respectively). Activity-based proteasome probe labeling and western blot analysis were performed at the indicated time points with the indicated concentrations of the drugs. Representative images of three independent experiments are shown. (D) Contraction velocity in Murine-CMs after 1 h of treatment with DMSO, CFZ (2,500 nM), CHX (20 μg/mL) or CFZ + CHX combination; the data are presented as the mean ± SD (*n* = 5 independent experiments in duplicate). Statistical significance was tested with two-way ANOVA and Bonferroni correction. A p-value < 0.05 is considered statistically significant. See also Figures S5E–S5G. atRA, all-trans retinoic acid; BTZ, bortezomib; CFZ, carfilzomib; CHX, cycloheximide; CM, cardiomyocytes; DMSO, dimethyl sulfoxide; MFI, median fluorescence intensity.

Our omics data suggested that the CFZ-mediated rapid contractile decrease is directly related to impaired protein turnover and transcriptional changes mediated by several transcription factors (such as Mef2 or cAMP-responsive elements). Subsequently, using H9c2 cardiomyoblasts, we functionally validated that CFZ treatment led to the rapid phosphorylation of Ca^2+^/calmodulin-dependent protein kinase II (CaMKII) and downstream factors Mef2a and Creb (Figure 5A), in contrast to CFZ and atRA co-treatment.

BiP (Binding Immunoglobulin Protein, an ER chaperone) is a central protein for chaperoning activity in the sarcomplasmic reticulum (SR). We equipped H9c2 cells with a BiP-mGFP construct that allows monitoring of BiP mobility and trafficking in living cells after photobleaching (FRAP).^42^ CFZ treatment increased the half-life of fluorescence recovery, indicating BiP to be more mobile early after proteasome inhibition, opposing to an effect induced by dithiothreitol (DTT) or ER stressors^42^ and suggesting no UPR induction in this time frame (Figure 5B). However, over a 20-min time window, CFZ induced accumulation of K48-ubiquitinated protein substrates (polyUb proteins) in the H9c2 cells, queuing for proteasomal degradation, which was partly prevented by atRA co-treatment (Figure 5C). Importantly, CFZ inhibited proteasome β5 subunits already after 2 min, whereas it co-inhibited the β2 and β1 subunits after 5 min. Co-treatment with atRA slowed the acute inhibition of the β2 and β1 proteasome subunits, likely preventing the rapid accumulation of polyUb proteins. Consistently, pretreatment with cycloheximide, a known inhibitor of translation elongation, prevented CFZ-mediated cardiotoxicity in murine-CMs (Figure 5D). These findings align with our *in vivo* observations, where atRA co-treatment resulted in a reduced/slower inhibition of proteasome activity. Notably, we ruled out that co-treatment with atRA would decrease the cytotoxic activity of CFZ in PI-naive (AMO-1 and MM1S) or PI-resistant (AMO-BTZ, AMO-CFZ, and MM1S-BTZ) myeloma cells *in vitro* (Figures S5E–S5G). Together, these results suggest that the swift and effective proteasome inhibition provided by high-dose CFZ impairs balance between protein synthesis and degradation, due to non-functional UPS and accumulation of defective proteins in early phases.

### Carfilzomib-mediated β5 and β2 proteasome inhibition causes a metabolic impairment and energy depletion in the murine heart

Next, we delineated the early metabolic changes occurring after high-dose CFZ treatment and investigated to what extent they are improved by atRA. For this, we analyzed the metabolites in murine hearts after 1 h of treatment with vehicle BTZ, CFZ, or CFZ and atRA at previously established doses using untargeted metabolomics.

**Figure 6:**
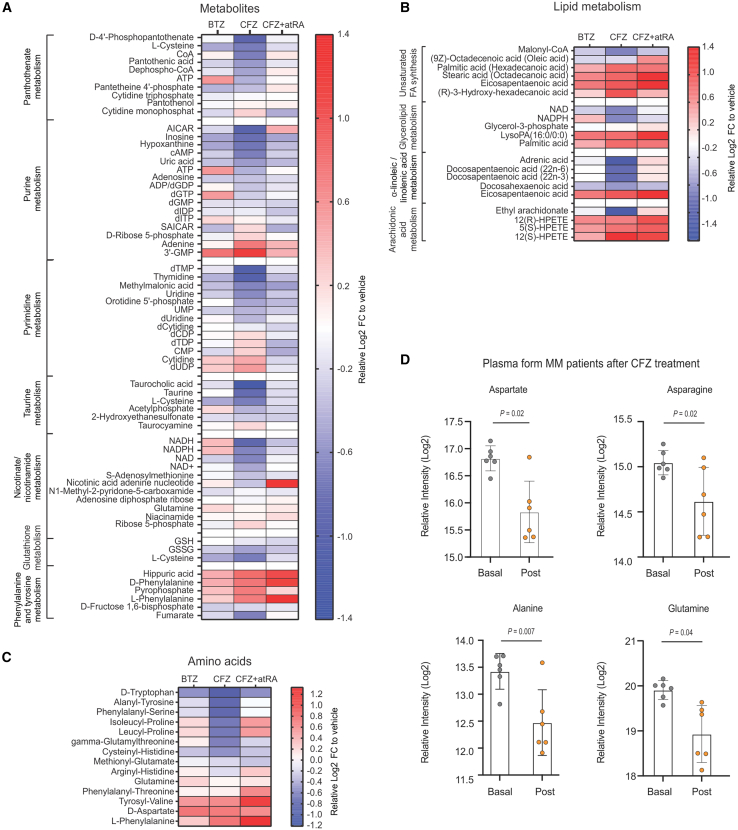
CFZ-mediated proteasome inhibition causes metabolic and amino acid depletion in murine hearts, consistent with energy deprivation (A) Heatmap depicting differences in the levels of cardiac-specific metabolites following 1 h *in vivo* BTZ (1 mg/kg), CFZ (16 mg/kg), or CFZ + atRA (16 mg/kg and 1 mg/kg, respectively) treatment after normalization to vehicle treatment and presented as the Log2 FC to vehicle (t test, *p* < 0.05). (B) Heatmap depicting differences in the levels of cardiac-specific lipid metabolites following 1 h *in vivo* BTZ (1 mg/kg), CFZ (16 mg/kg) or CFZ + atRA (16 mg/kg and 1 mg/kg, respectively) treatment after normalization to vehicle treatment and presented as the Log2 FC to vehicle (t test, *p* < 0.05). (C) Heatmap depicting differences in the levels of cardiac-specific amino acids following 1 h *in vivo* BTZ (1 mg/kg), CFZ (16 mg/kg), or CFZ + atRA (16 mg/kg and 1 mg/kg, respectively) treatment after normalization to vehicle treatment and presented as the Log2 FC to vehicle (t test, *p* < 0.05). In all experiments, *n* = 3 mice per group. (D) Relative levels of circulating amino acids in MM patients before (basal level) and 1–8 h after CFZ treatment; (*n* = 6 MM pt). Statistical significance was tested with a t test. A p-value < 0.05 is considered statistically significant. See also Tables S10 and S11, and Figures S6 and S7. atRA, all-trans retinoic acid; BTZ, bortezomib; CFZ, carfilzomib; FC, fold change.

Enrichment analysis of the deregulated metabolites after PI treatment and their classification using the KEGG database revealed that CFZ, but not BTZ treatment, led to depletion of a significant number of intracellular metabolites (Figures S6A and S6B), related to pantothenate and CoA synthesis, taurine metabolism, glutathione, and purine and pyrimidine metabolism (especially decreased adenosine and thymidine metabolites: AICAR, inosine, cAMP, ATP, and dTMP; Figures 6A and S6A; Table S10). Importantly, only CFZ induced changes in cellular lipids, observed as a depletion of α-linoleic and linolenic acid metabolites (Figure 6B and Table S10). CFZ treatment led to deregulation of cellular amino acids observed as a depletion of D-tryptophan, alanyl-tyrosine, and phenylalanine-serine and accumulation of tyrosyl-valine, D-aspartate, and L-phenylalanine (Figure 6C). These data are in line with recent research showing CFZ-mediated amino acid scarcity in myeloma cells recovering after CFZ treatment *in vitro*.^7^

Next, enrichment analysis of the metabolites that accumulated after PI treatment, followed by KEGG classification, revealed CFZ-related changes in phenylalanine metabolism, arachidonic acid metabolism generating hydroperoxy fatty acids, and in the synthesis of unsaturated fatty acids (Figures S6C and S6D). Our data further suggest that the metabolic changes induced by CFZ are local and specific in the heart, as only a few significant changes were observed in the circulating metabolites in murine plasma; they did not correlate with findings in the murine heart and overlapped between BTZ and CFZ treatment (Figures S6A and S6B; Table S11).

AtRA ameliorated the metabolic disturbances induced by CFZ in the heart by preventing metabolic decrease: the decrease in the metabolism of pantothenate, CoA, purines, pyrimidines, nicotinate, nicotinamide, and α-linoleic and linolenic acids as well as in some amino acids (Figures 6A–6C and Table S10), consistent with its improvement of CFZ-mediated effect on ribosomal proteins. However, it did not prevent CFZ-induced accumulation of metabolites (Figures 6A and 6B) and had only minor effects on CFZ-impaired metabolites in murine plasma (Figures S7A and S7B).

Finally, we examined the levels of circulating metabolites in MM patients exposed to CFZ by comparing the serum prior to and 1–8 h after CFZ infusion. We found decreased levels of circulating amino acids aspartic acid, asparagine, glutamine, and alanine (Figure 6D), consistent with the sharp decrease in intracellular glutamine after CFZ treatment reported previously.^7^ The prognostic value of the level of these amino acids in MM patients warrants further investigation. Taken together, these findings show that CFZ rapidly changes metabolic homeostasis in the heart, associated with purine/pyrimidine depletion and impairment of cardiac amino acids and lipids, suggesting impairment of ATP and protein translation.

### All-trans retinoic acid prevents CFZ-induced cardiotoxicity by alleviating CFZ-induced metabolic depletion and increased cardiac angiotensin A levels

AtRA co-treatment improved the lack of metabolic flow but, in general, did not prevent accumulation of metabolites following CFZ treatment. We searched for individual metabolites in murine hearts that significantly accumulated after CFZ treatment (but not after BTZ treatment) and whose accumulation was prevented by CFZ + atRA co-treatment.

**Figure 7:**
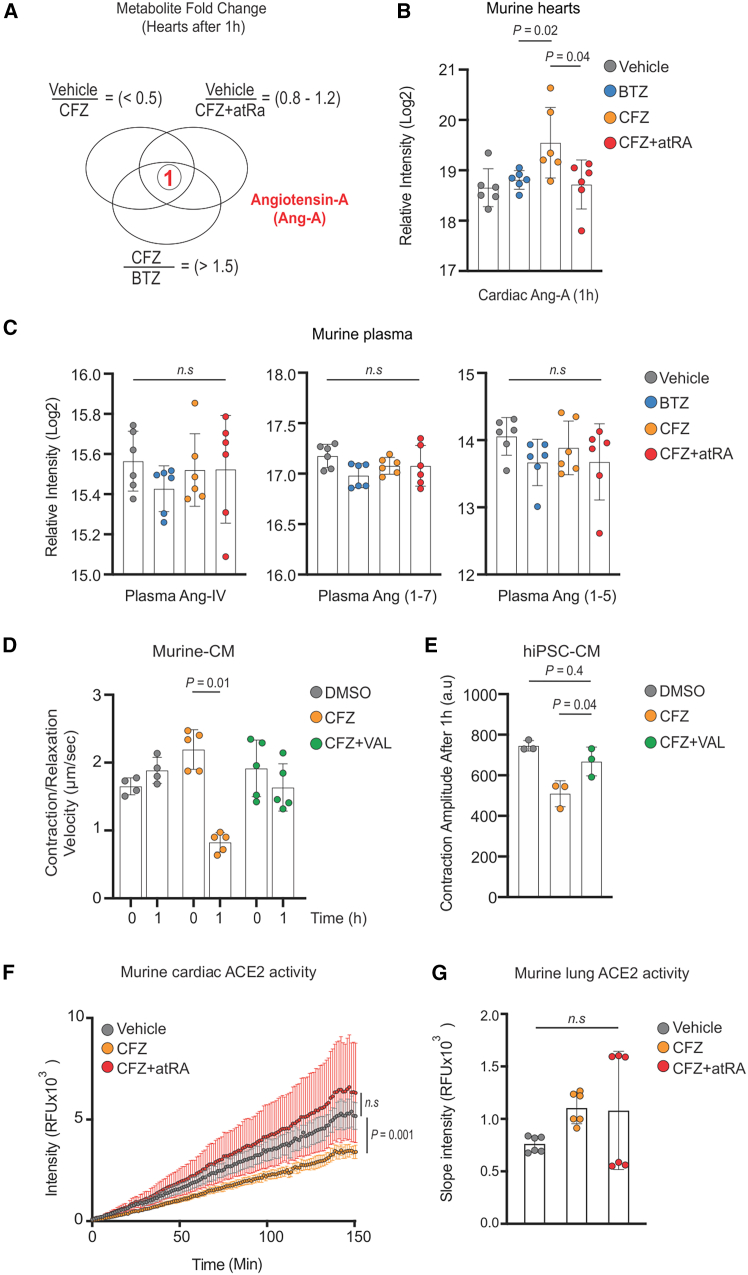
AtRA prevents CFZ-induced cardiotoxicity by decreasing cardiac angiotensin levels (A) Strategy for the selection of specific metabolites induced by CFZ. (B) Levels of murine cardiac angiotensin A measured at the end of 1 h of treatment with BTZ (1 mg/kg), CFZ (16 mg/kg) or CFZ + atRA (16 mg/kg and 1 mg/kg, respectively); the data represent the mean ± SD (*n* = 6 mice per cohort). (C) Plasma levels of angiotensin metabolites after treatment for 1 h with BTZ (1 mg/kg), CFZ (16 mg/kg), or CFZ + atRA (16 mg/kg and 1 mg/kg, respectively); the data represent the mean ± SD (*n* = 6 mice per cohort). (D) Contraction velocity in Murine-CMs after 1 h pulse treatment with CFZ (2,500 nM), VAL (200 nM), or their combination; the data represent the mean ± SD (*n* = 5 independent experiments in duplicate). (E) Effect of CFZ (2,500 nM), VAL (200 nM), and their combination on beating of hiPSC-CMs; the data represent the mean ± SD (*n* = 3 independent experiments). (F) Murine cardiac Ace2 enzymatic activity 1 h after treatment with CFZ (16 mg/kg) or CFZ + atRA (16 mg/kg and 1 mg/kg, respectively); the data represent the mean ± SD (*n* = 2 independent experiments in triplicate). (G) Murine lung Ace2 enzymatic activity 1 h after treatment with CFZ (16 mg/kg) or CFZ + atRA (16 mg/kg and 1 mg/kg, respectively); the data represent the mean ± SD (*n* = 2 independent experiments in triplicate). For all data, if there were more than 2 groups, statistical significance was tested with two-way ANOVA and Bonferroni correction. A p-value < 0.05 is considered statistically significant. Ace2, angiotensin converting enzyme 2; atRA, all-trans retinoic acid; BTZ, bortezomib; CFZ, carfilzomib; hiPSC-CMs, human induced pluripotent stem cell-derived cardiomyocytes; Murine-CM, murine neonatal cardiomyocytes; VAL, valsartan.

Using stringent criteria (Figure 7A), only one metabolite, angiotensin A (Ang-A), accumulated significantly in the hearts after CFZ exposure, in contrast to treatment with BTZ or CFZ and atRA co-treatment (Figure 7B). Ang-A is a product of angiotensin II (Ang-II) decarboxylation; which has 90% of the potency of Ang-II^43^ and triggers similar hemodynamic effects via the Ang-II type 1 receptor (Agt1r) directly in the heart.^44^ Importantly, Ang-A induction was not detected in murine plasma and the levels of angiotensin IV, angiotensin (1–7), and angiotensin (1–5) were not different after the treatments (Figure 7C). Likewise, the levels of circulating angiotensin (1–5), angiotensin (1–7), and angiotensin (5–8) did not differ within 8 h of CFZ administration in MM patients, supporting our results of local activity of the cardiac renin-angiotensin system (RAS) after CFZ-mediated proteasome inhibition.

To dissect whether local angiotensin signaling is involved in CFZ-induced contractile impairment, we evaluated the effects of the Agt1r inhibitor valsartan on the CFZ-induced functional effect in cardiomyocytes *in vitro*. Treatment with CFZ and valsartan prevented contractile velocity impairment in CFZ-treated Murine-CMs (Figure 7D) and rescued acute CFZ-induced contractile dysfunction in hiPSC-CMs (Figure 7E). To further mechanistically confirm the local activation of Ang-A, we analyzed the enzymatic activity of angiotensin converting enzyme 2 (Ace2, peptidyl-dipeptidase A2), which cleaves Ang-II into Ang (1–7) and decreases the activity and deleterious effects of Ang-II. We found decreased activity of Ace2 in murine hearts, after *in vivo* CFZ treatment (Figure 7F), and atRA co-treatment restored Ace2 activity in the hearts. Importantly, Ace2 activity was decreased specifically in the murine hearts and not in the lungs (Figure 7G). Thus, our data suggest that acute proteasome inhibition by CFZ leads to early metabolic changes in the heart, compatible with heart failure and local production of Ang-A in the heart. AtRA prevents CFZ-induced cardiotoxicity by lowering the metabolic stress and angiotensin activity in the heart.

## Discussion

The mechanism of cardiovascular adverse events, which are observed in up to 50% of patients treated with the second-generation PI CFZ,^9^ remains incompletely understood. In MM patients, CFZ administration causes proteasome β5 subunit inhibition at 20 mg/m^2^ and β2 subunit co-inhibition at doses over 36 mg/m^2^.^6^ Here, using multiple *in vitro* and *in vivo* models, we found that low-dose CFZ (625 nM or 4 mg/kg), which mimics the clinical dose of 20 mg/m^2^ in terms of selective proteasome β5 inhibition, did not affect cardiomyocyte contractility. In contrast, high-dose CFZ (2,500 nM or 16 mg/kg), reflecting the inhibition profile of proteasome β5, β2, and β1 subunits observed in patients receiving doses above 36 mg/m^2^, resulted in a rapid reduction in contractile velocity and force *in vitro* and induced bradycardia *in vivo*—presumably due to β2 subunit co-inhibition. The data align with previous observation by echocardiography, where CFZ significantly reduced fractional shortening percentage, suggesting reduced contractility also *in vivo*.^12^^,^^13^ Moreover, the decreased contractility phenotype may reflect issues with energy metabolism, which might influence ion channel function, or issues with autonomic control mechanisms potentially resulting in a decreased heart rate, which has been observed during heart failure.^45^^,^^46^

More importantly, the impaired contractile velocity was observed after *in vitro* treatment with a combination of β5 and β2 subunit-selective inhibitors, ruling out the possibility to be caused by an off-target of the drug and suggesting the velocity impairment to be related to β2 subunit co-inhibition. The phenotype was not observed after BTZ-type β5 and β1 proteasome inhibition or a combination of β5 and β1 subunit selective inhibitors, consistent with the higher rates of CVAEs in MM patients after high-dose CFZ treatment in contrast to BTZ treatment.^9^^,^^47^

By applying an integrated multiomics approach *in vitro* and *in vivo*, we found that β2 co-inhibition achieved with high-dose CFZ (functionally strongly impairing proteasome function) leads to a swift impairment of cardiac structural proteins and protein translation machinery as early as 1 h post-treatment, preceding the induction of the UPR, which typically occurs at later time points. This is associated with a transcriptomic signature reflecting changes in structural proteins directly in cardiomyocytes *in vivo*. Thus, our data suggest that early cardiac remodeling and impaired contractile velocity are direct consequences of proteasome inhibition, rather than secondary downstream effects. Consistently, disruption of turnover of contractility components and sarcomere cardiomyocyte proteins was associated with contractile dysfunction in CFZ-treated hiPSC-CMs and persisted for 24 h after the treatment.^15^^,^^16^

CFZ-specific β2 co-inhibition *in vivo* significantly decreased the metabolic flow to produce ATP, while BTZ, providing β5 and β1 proteasome inhibition, had a minor effect. Our findings correlate with previous work in the field, showing ATP deficiency, a hallmark of failing hart,^48^^,^^49^ to be associated with CFZ treatment in hiPSC-CMs.^16^ At the same time, the CFZ-mediated drop in ATP was rescued by atRA supplementation, although it did not prevent the drop in OXPHOS *in vitro*. Thus, our data suggest that CFZ-induced contractility impairment is related to an ATP decrease, which, however, is not solely caused by decrease in OXPHOS.

Proteasome inhibition in cardiomyocytes highlighted the importance of the retinol pathway. Cardiac atRA levels decline during heart failure, and increased ALDH1A1 levels have previously been observed in patients with idiopathic dilated cardiomyopathy (IDCM).^50^ In our *in vitro* and *in vivo* models, treatment with both PIs led to the accumulation of Aldh1a1. CFZ treatment caused more significant accumulation of Aldh1a1 than BTZ treatment, consistent with stronger functional proteasome inhibition and suggesting that an increased Aldh1a1 might be an early marker of damage after functional proteasome inhibition. AtRA has been shown to protect the heart from pressure overload–induced heart failure and myocardial injury, and the mechanisms underlying the protective effects of atRA are multiple, including modulation of protein synthesis,^50^ and decreased JNK-1 stress signaling.^51^ Likewise, the mechanisms underlying the protective effects of atRA in CFZ-induced cardiotoxicity are broad, but according to our evidence, they concentrate around balancing protein homeostasis, stress signaling, and autophagy induction. Autophagy promotes protein degradation, and therefore has been proposed to maintain amino acid pools to sustain protein synthesis during metabolic stress.^52^ In concordance with our data, CFZ-mediated cardiotoxicity is linked to autophagy impairment, which has been improved by metformin co-treatment.^12^ Our data further show that atRA lowers the level of CFZ-induced cardiac Ang-A, a derivative of Ang-II. Ang-II is produced locally in the human heart^53^ and leads to cardiac remodeling via the AGTR1 receptor.^54^ At the same time, decreased ACE2 activity, which results in increased Ang II, is associated with JNK-1 stress signaling.^55^ Our data show that CFZ treatment locally activates Ang-A and reduces Ace2, possibly due to increased cellular stress, which is, however, connected with its downstream pathologic signaling via Agtr1. This correlates with previous data showing that genetic ablation of Ace2 leads to cardiac hypertrophy and increased levels of Ang-II.^56^ Importantly, blockade of Ang-A activity directly in the cardiomyocytes through AGTR1/Agtr1 inhibition with valsartan prevented CFZ-induced toxicity in human and murine models, suggesting involvement of RAS in CFZ-induced cardiotoxicity. These findings need to be validated *in vivo* and lack of such confirmation represent the limitation of current study. However, a therapy containing an ACE inhibitor promptly improved CFZ-related cardiac dysfunction in a small cohort of MM patients,^57^ suggesting an effect of ACE inhibitors in patients. Overall, our results are consistent with previous data showing the role of atRA in the regulation of RAS,^58^^,^^59^ and reveal a novel mechanism for the prevention of CFZ-mediated cardiomyocyte toxicity. These results align with recent data showing that atRA, via RORδ receptors, enhances sensitivity to CFZ and re-sensitizes CFZ-resistant MM cells to CFZ *in vitro*.^60^ The data suggest as well that the addition of atRA to CFZ treatment improves the anti-MM effect without increasing acute CVAEs, providing a rationale for trials combining atRA and CFZ for MM therapy.

In summary, we provide evidence of a specific mechanism for cardiotoxicity following high-dose CFZ treatment involving proteasome β2 co-inhibition that impacts translation machinery and protein turnover, thus modulating cardiac metabolism, and cardiac Ang-A synthesis. Our results are consistent with the available clinical data showing higher rates of CVAE for CFZ than for the other PIs^61^ and observed for higher doses of CFZ.^62^ Our study also highlights the use of atRA and/or RAS modulators such as valsartan during CFZ treatment, which may enable mitigation of the acute cardiovascular toxicity of CFZ and improve MM control. Future clinical studies specifically designed to investigate these topics are needed to reach definitive conclusions.

### Limitations of the study

We acknowledge certain limitations of our study. First, H9c2 cardiomyoblasts are model, non-beating cells that can be manipulated *in vitro*, which allows for particular functional studies; however, they do not fully represent mature and beating cardiomyocytes. Therefore, further validation in a clinically relevant myeloma cardiotoxicity model is necessary. Second, while our unbiased LC-MS/MS analysis identified multiple pathways and proteins deregulated by CFZ, comprehensive validation of all potential hits was beyond the scope of the current manuscript. Future studies will be needed to explore these targets in greater detail. Third, the number of patient samples used to assess amino acid depletion was limited, and additional studies are required to better understand the clinical significance of these findings.

## Resource availability

### Lead contact

Requests for further information and resources should be directed to and will be fulfilled by the lead contact, Lenka Besse (lenka.besse@med.muni.cz).

### Materials availability

This study did not generate new unique reagents.

### Data and code availability

• The raw untargeted metabolomics data are available through the NIH Common Fund’s National Metabolomics Data Repository (NMDR) at the Metabolomics Workbench (https://www.metabolomicsworkbench.org): Murine heart and plasma samples can be accessed using NMDR: ST001846, ST001858. MM patients’ samples can be accessed using NMDR: ST001925. The raw data from label-free proteomic experiments conducted on murine heart tissue have been deposited in the PRIDE database (https://www.ebi.ac.uk/pride/archive) under the accession ID PXD041167. The raw data from label-free proteomic experiments conducted *in vitro* have been deposited in the PRIDE database under the accession ID PXD029077. The single-cell RNA-seq data have been deposited in the BioStudies database (https://www.ebi.ac.uk/biostudies/) using EMBL-EBI: E-MTAB-11028. All data are publicly available without restriction.
• Processed data in the form of Seurat objects (.rds files), containing cell barcode ID–to–cluster mappings along with additional downstream analysis outputs and the R code used for data analysis are available on Figshare: https://doi.org/10.6084/m9.figshare.c.6716034.v1.
• An interactive Shiny app for viewing the ECGs is available at https://maxmendezl.shinyapps.io/ShinnyApp/.
• Any additional information is available from the lead contact upon request.

## Acknowledgments

The work was supported by Cantonal Hospital St. Gallen Research Committee internal grants no. 17/09 and 20/04, 10.13039/501100013362Swiss Cancer Research Foundation (KFS-4990-02-2020), by the 10.13039/501100022716Swiss Cancer Foundation and by the project National Institute for Cancer Research (Program EXCELES, ID project no. LX22NPO5102)—Funded by the European Union—Next Generation EU.

## Author contributions

M.M.-L. and A.B. designed and performed the experiments using Murine-CM, H9c2 cardiomyoblasts, and *in vivo* models, and designed the figures. C.Z. performed the experiments with hiPSC-CMs. C.P.-S., C.G.-C., A.D.M., and B.L. contributed to the scRNA-seq experiments. M.L. analyzed the scRNA-seq data. D.B. and V.R. provided hESC-CM. S.K. and J.P. conducted the atomic force microscopy experiments. B.I.F. and H.S.O. provided the activity-based probes, subunit selective inhibitors, and data from *in vitro* LC-MS/MS experiment. X.Z. and L.R. provided the MM samples. M.M.-L. and L.B. wrote the original manuscript, evaluated the data, and performed the literature search. L.B. and C.D. conceptualized the study, secured funding, and provided critical review of the data. All authors reviewed and edited the manuscript.

## Declaration of interests

The authors declare no competing interests.

## STAR★Methods

### Key resources table

**Table:** 

REAGENT or RESOURCE	SOURCE	IDENTIFIER
**Antibodies**		

Phospho-CaMKII	Cell Signaling Technology	12716; RRID:AB_2713889
CaMKII (pan) (D11A10)	Cell Signaling Technology	4436; RRID:AB_10545451
Phospho-CREB	Thermo Fisher Scientific	MA5-11192; RRID:AB_10986840
CREB	Thermo Fisher Scientific	MA1-083; RRID:AB_558523
Phospho-Mef2a	Thermo Fisher Scientific	PA5-37642; RRID:AB_2554250
Mef2a	Thermo Fisher Scientific	PA5-27380; RRID:AB_2544856
K48-Ub (poly-Ub)	Thermo Fisher Scientific	MA5-35382; RRID:AB_2849283
Aldh1a1	Santa Cruz Biotechnology	SC-374149; RRID:AB_10917910
Tubulin	Proteintech	HRP-66031; RRID:AB_2687491
GAPDH	Proteintech	HRP-60004; RRID:AB_2737588

**Biological samples**		

Serum from 6 MM patients	This paper	N/A

**Chemicals, peptides, and recombinant proteins**		

Activity based probes	Leiden Uni	de Bruin et al., 2016^63^
NC001 (β1c/i selective inhibitor)	Leiden Uni	van der Linden et al., 2012^24^
LU-102 (β2c/i selective inhibitor)	Leiden Uni	Geurink et al., 2013^25^
NC005 (β5c/i selective inhibitor)	Leiden Uni	Britton et al., 2009^26^
A37	Tocris	5802
All-trans-Retinoic-Acid	Sigma-Aldrich	R2625
Bortezomib (PS-34)	Selleck Chemicals	S1013
Captisol	Ligand Pharmaceuticals	N/A
Carfilzomib (PR-171)	Selleck Chemicals	S2853
Cycloheximide	Sigma-Aldrich	C-7698
DMEM Medium	Sigma-Aldrich	D6429
Horse Serum	Biowest	S0910-500
Ketamine Hydrochloride	Sigma-Aldrich	Y0000450
Laminin	Sigma-Aldrich	L2020
Medium 199	Sigma-Aldrich	M4530-100ml
Retinaldehyde (Retinal)	Sigma-Aldrich	R2500
Sep-Pak tC18 cc Cartridge	Waters	186004618
Trypsin (Sequencing Grade)	Promega	V511A
Valsartan	Sigma-Aldrich	SML0142

**Critical commercial assays**		

Cell Counting Kit (CCK-8)	GLP Bio	GK10001
Neonatal Heart Dissociation Kit	Miltenyi Biotec, Germany	130-098-373
Aldefluor TM Kit	STEMCELL Technologies	01700
MitoXpress Xtra Oxygen Consumption Assay	Agilent Technologies, CA, USA	MX-200-4 and HS-100D-1
Ace2-quenched fluorogenic substrate	MedChem Express	HY-P2536
Chromium Single Cell 3ʹ GEM, Library & Gel Bead Kit v3	10X Genomics	PN-1000075
Chromium Chip B Single Cell Kit	10X Genomics	PN-1000073

**Deposited data**		

ShinyApp for an interactive view of the ECGs	This paper	https://maxmendezl.shinyapps.io/ShinnyApp/
Murine plasma samples metabolomics	https://www.metabolomicsworkbench.org	ID: ST001858
Murine heart samples metabolomics	https://www.metabolomicsworkbench.org	ID: ST001846
MM patients plasma samples metabolomics	https://www.metabolomicsworkbench.org	ID: ST001925
Label-free proteomics performed *ex vivo* from murine hearts	PRIDE Database	ID: PXD041167
Label-free proteomics performed *in vitro*	PRIDE Database	ID: PXD029077
Single-cell RNA-seq data	BioStudies database (https://www.ebi.ac.uk/biostudies/)	ID: E-MTAB-11028
Processed data in form of the Seurat objects as .rds files that contain cell-barcode-ID-to-cluster mappings	Figshare	
Cell-barcode-ID-to-cluster mappings as text files	Figshare	https://figshare.com/s/95671820aa4ae838fdeb https://figshare.com/s/14d6ffad4a9df9004357 https://figshare.com/s/1884d1d9213526ec9039 https://figshare.com/s/38c8eb5497120a2d3d9f
R codes used for analysis data	Github	https://github.com/MaxMendezL/CardiotoxicityCFZ

**Experimental models: Cell lines**		

H9c2 (2-1)	ATCC	CRL-1446
hIPSC-CM (iCell cardiomyocytes^2^) 01434	Cellular Dynamics	C1016
Murine-CM	Balb/cJ mice	N/A
hESC lines	Pesl et al., 2014^64^	N/A
AMO-1	DSMZ	ACC 538
MM1S	ATCC	CRL-2974
AMO-BTZ derivatives from AMO-1 cell line	Soriano et al., 2016^65^	N/A
AMO-CFZ derivatives from AMO-1 cell line	Soriano et al., 2016^65^	N/A
MM1S-BTZ derivatives from MM1S cell line	This paper	N/A
Lenti-X™ 293T Cell Line	Takara Bio	632180

**Experimental models: Organisms/strains**		

BALB/c	Charles Rivers	000651

**Recombinant DNA**		

Ub-G76V-GFP	Dantuma et al., 2000^29^	Addgene #11941
pMD2.G	Trono lab	Addgene #12259
psPAX	Trono lab	Addgene #12260
BiP-mGFP	Lai et al., 2010^42^	Addgene #62231

**Software and algorithms**		

Prism software v. 6.68 and v.8	NCH Software, CO, USA	
MATLAB R2020a	MathWorks, USA	R2020a - Updates to the MATLAB and Simulink product families - MATLAB & Simulink (mathworks.com)
“Musclemotion” for ImageJ	Sala et al., 2018^66^	GitHub - l-sala/MUSCLEMOTION: Open-source ImageJ plugin to quantify cardiomyocyte and cardiac muscle contraction *in vitro* and *in vivo*
CellRanger (v3.0.2)	Zheng et al., 2017^67^	GitHub - 10XGenomics/cellranger: 10x Genomics Single Cell 3' Gene Expression and VDJ Assembly
R v.3.6.2 using the scater R/Bioconductor package (v1.14.6)	McCarthy et al., 2017^68^	
R v.3.6.2 using the Seurat package (v.3.1.4)	Stuart et al., 2019^69^	
AnnotationDbi package for R	Pages et al., 2021^70^	AnnotationDbi: Manipulation of SQLite-based annotations in Bioconductor
ClusterProfiler package for R	Yu et al., 2012^71^	Bioconductor - clusterProfiler

### Experimental model and subject details

#### Isolation and culture of murine neonatal cardiomyocytes

Murine-CMs were isolated from 1 to 3-day-old pups of BALB/c mice with the Neonatal Heart Dissociation Kit (Miltenyi Biotec, Bergisch Gladbach, Germany). The cardiomyocytes were kept in culture as previously described with few modifications.^30^ Briefly, 1.5x10^5^ cells were plated onto laminin-coated 48-well plates with medium containing 65% DMEM, 19% M-199 Medium (Sigma-Aldrich, St. Louis, MO, USA), 10% Horse Serum (Biowest, Nuaillé, France) and 6% Fetal Calf Serum (FCS, Sigma-Aldrich). The FCS was removed from the culture medium after 24 h to avoid fibroblast overgrowth. Cardiomyocytes were cultured until a confluent beating monolayer was formed. All experiments were performed 3-4 days after isolation.

#### Culture of hiPSC-CMs

Commercially available hiPSC-CMs (iCell^2^ cardiomyocytes) were obtained from Cellular Dynamics International Inc. (Madison, WI, USA). Cryopreserved iCell^2^ cardiomyocytes were rapidly thawed, diluted in iCell^2^ plating medium and seeded onto 35 mm round dishes with 7 mm diameter wells and a glass bottom (MatTek Corp., Ashland, MA, USA) coated with human fibronectin (Sigma-Aldrich). After 48 h, the medium was changed to maintenance medium, after which it was changed every two days.

#### Generation of human embryonic stem cell-derived cardiomyocytes (hESC-CMs)

hESC-CMs forming cardiomyocyte clusters or embryonic bodies (EB) were generated as previously described.^64^ Beating hESC-CMs were differentiated for 28 days and seeded onto adherent 30 mm plates (TPP Techno Plastic Products AG, Trasadingen, Switzerland) to form clusters. Before experiments, the EBs were cultured in Tyrode medium (135 mM NaCl, 10 mM HEPES, 5.4 mM KCl, 0.9 mM MgCl_2_, 0.33 mM NaH_2_PO4, pH 7.4) supplemented with 5.5 mM glucose and 1.8 CaCl_2_ at 37°C in a standard incubator.

#### H9c2 rat cardiomyoblast cells

H9c2 rat cardiomyoblasts (ATCC, Manassas, VA, USA) were kept in culture with DMEM medium (Sigma-Aldrich) supplemented with 10% FCS (Sigma-Aldrich). Cells lines were STR-typed to confirm the authenticity (DSMZ, Braunschweig, Germany) and were routinely tested for mycoplasma contamination with a MycoAlert Mycoplasma Detection Kit (Lonza, Basel, Switzerland).

#### MM patients

Serum from 6 MM patients undergoing CFZ-based therapy was collected prior to and 1-8 h after CFZ administration at the University Hospital of Würzburg (paired samples). All procedures were performed in accordance with local ethical standards, according to the Declaration of Helsinki and approved by an Ethical Committee with number: 197/20-am. Detailed characteristics of the MM patients whose samples were used in the study are provided in Table S12. The cohort size was too small to draw any conclusions regarding the influence of age or sex on the evaluated parameters.

#### MM cell lines

Proteasome inhibitor naïve (AMO-1, MM1S), and resistant (AMO-BTZ, AMO-CFZ, MM1S-BTZ)^65^ cells were maintained in RPMI-1640 culture medium (Sigma-Aldrich) supplemented with 10% FCS (Sigma-Aldrich), 100 μg/ml streptomycin and 100 U/ml penicillin (Sigma-Aldrich). The cell lines were STR-typed to confirm the authenticity (DSMZ) and routinely tested for mycoplasma contamination (MycoAlert Mycoplasma Detection Kit, Lonza). The data on cell lines are presented in Figures S5E–S5G.

#### BALB/c mice

Eight- to twelve-week-old BALB/c male and female mice (Charles River Laboratories, Sulzfeld, Germany) were kept in isolated and ventilated cages with food *ad libitum*. The study was carried out in accordance with the 3Rs principles, and the experiments were approved by the local Committee for Animal Experiments (St. Gallen, Switzerland), approval no. 33037.

### Method details

#### Chemicals

A complete list of chemicals used in the study is provided in STAR Methods (key resources table). BTZ inhibited β5 at 75 nM and β5 + β1 proteasome subunits at 300 nM, whereas CFZ inhibited β5 and 625 nM and β5 + β2 subunits at 2500 nM. Selective proteasome-subunit inhibitors were provided by the Department of Chemistry, Leiden University,^24^^,^^25^^,^^26^ and were used at the following concentrations: for β5 inhibition, NC005: 10 μM, for β1 inhibition NC001: 5 μM and for β2 inhibition, LU102: 3 μM). atRA was solubilized in DMSO in a light-protected environment and further diluted in PBS for experiments. The concentrations were determined previously to be 15 μM for *in vitro* experiments^72^ and 1 mg/kg for intraperitoneal (*i.p.*) injection in mice.^73^

#### Motion vector image analysis of Murine-CMs

For vector analysis, 1.5x10^5^ Murine-CMs were isolated, plated and cultured until a monolayer of beating cells was formed. Between days 3-4, a 10-second video (60 frames per second) was taken before and after 1 h incubation with the respective compounds. Video files were converted to TIFF image sequences with Prism software version 6.68 (NCH Software, Canberra, Australia) and motion vectors were extracted with a publicly available image processing tool for MATLAB R2020a (MathWorks Inc., Natick, MA, USA).^28^ Vectors were extracted as contraction or relaxation velocities (μm/sec) before or after 1 h treatment. To address variability between cardiomyocytes from different isolations, vector velocities were normalized and expressed as the contraction/relaxation quotient (μm/sec) before (0 h) and after (1 h) exposure to the respective treatment.

#### Video analysis of hiPSC-CM contractility

A modified GoPro HeroBlack 6 camera (Back-Bone Gear Inc., Kanata, Canada) was used to record short video sequences at a high frame rate (240 frames per second) of cell cultures in a heating chamber with a warmed lid and temperature controller (Ibidi, Martinsried, Germany) on the stage of an inverted microscope (Nikon Eclipse TE2000-U) with a Nikon Plan Fluo 10x/0.3 phase contrast lens. The cultures were allowed to warm to 37°C for 10 minutes, and DMEM culture medium containing 25 mM HEPES buffer was used for recordings. The open-source macro “Musclemotion” for ImageJ^66^ was then used on an Apple MacBook Pro to extract motion data from videos after format conversion and data reduction steps.

#### Atomic force microscopy (AFM)

Atomic force microscopy measurements were performed according to a previously described protocol.^33^ For the experiments, the EBs were placed on an inverted microscope (Olympus IX-81S1F-3, Tokyo, Japan) with the AFM recording head (BioAFM NanoWizard 3, JPK, Berlin, Germany) placed on top. The cantilever (NITRA-tall B, AppNano, Mountain View, CA, USA) was carefully positioned on the beating EBs and the data were acquired with Scanning Probe Microscope Software v.4 (JPK, Berlin, Germany). The temperature was maintained at 37°C with a petri dish heater (JPK). Data acquisition was performed at 5 kHz and analyzed with an in-house algorithm for MATLAB 2019a (MathWorks). Recordings were made before and after 1 h of treatment with either DMSO or 2500 nM CFZ.

#### OXPHOS measurement

OXPHOS measurement was performed according to the manufacturer’s recommendations using a MitoXpress Xtra Oxygen Consumption Assay (Agilent Technologies, Santa Clara, CA, USA) on a Tecan Infinite M Plex instrument (Tecan Group, Männedorf, Switzerland). Briefly, 2x10^5^ Murine-CMs were seeded per well of a 96-well flat-bottom transparent plate and the cells were treated for 1 h with the specified compounds; afterward, the fluorescent compound was added. On top of each well, 100 μl of the oil was added, and the measurement was performed for 2 h.

#### Generation of the Ub^G76V^-GF-expressing H9c2 cell line (H9c2-Ub^GFP^) and assessment of functional proteasome inhibition

H9c2 cardiomyoblasts were transfected by the calcium-phosphate method with the Ub^G76V^-GFP vector (Addgene #11941, USA, a kind gift from Nico Dantuma), encoding a fluorogenic proteasome substrate that accumulates proportionally to the degree of functional proteasome inhibition, as specified before.^29^ Cells with stable Ub^G76V^-GFP expression were selected with G418 (500 ng/ml, Gibco/Thermo Fisher Scientific Waltham, MA, USA) for 5 days. On day 6, H9c2-Ub^GFP^ cells were exposed to 500 nM BTZ for 1 h and then sorted 8 h later to obtain the population with the highest GFP intensity, which was used for further experiments.

In the experiment, 1x10^5^ H9c2-Ub^GFP^ cells were incubated with the indicated concentrations of proteasome inhibitors for 1 h and then placed in drug-free medium. GFP intensity was quantified at the indicated time points by a BD Fortessa flow cytometer (BD Biosciences, San Jose, CA, USA).

#### Generation of the BiP-mGFP-expressing H9c2 cell line (H9c2-BiP)

H9c2 cardiomyoblasts were transduced with a fluorescent reporter for BiP mobility (Bip-mGFP; Addgene # 62231, a kind gift from Erik Snapp)^42^ via lentiviral transduction. Briefly, lentivirus was produced with the packaging plasmids pMD2.G and psPAX2 (Addgene #12259 and #12260, both gifts from Didier Trono) as specified elsewhere.^74^ Positive cells were enriched via cell sorting.

#### Confocal microscopy and FRAP

For FRAP analysis, images were acquired with an LSM 980 with Airyscan 2 confocal microscope (Carl Zeiss AG, Oberkochen, Germany) equipped with a live cell chamber (set at 37°C and 5% CO2) and ZEN software with a 63X oil-immersion objective. H9c2-Bip cells were seeded in an 8-well cell culture chamber on a cover glass, coated with laminin and incubated overnight. The following day, the cells were placed into a live cell chamber. The image was focused on GFP, and the SR areas in the cells were labeled. Cells were excited with a 488 nm laser, and the emission at 510 nm was recorded (GFP). Images were acquired with 16–bit image depth, and the recovery of fluorescence was followed for 10 min (1 image per 0.45 sec). Subsequently, the cells were treated with the respective chemicals, and bleaching and recovery monitoring was repeated in the same cells after 5 min and 60 min of treatment.

#### Activity based proteasome probes labeling, SDS-PAGE, western blotting and data analysis

The activity of proteasome subunits was assessed with activity-based proteasome probes (ABPP) as previously described.^63^ Briefly, equal amounts of proteins were extracted, labeled with the ABPP cocktail for 1 h and separated by SDS–PAGE on 12% NuPage™ bis-tris gels (Thermo Fisher Scientific). Proteasome subunit activity was visualized in gel with a Fusion Solo S Western Blot and Chemi Imaging System (Vilber-Lourmat, Collégien, France), and the band intensity was quantified using ImageJ software. A total of 30 μg of protein was loaded per well and used across all experiments. For the subsequent detection of protein levels and to control for equal loading, the gels were blotted as previously described^65^ and the membranes were incubated with anti-p-CaMKII, anti-CaMKII, anti-p-Mef2a, anti-Mef2a, anti-p-CREB, anti-CREB, anti-K48-Ub and anti-GAPDH and anti-αTubulin as loading controls. The intensity of specific bands was quantified using ImageJ software.

#### Label-free proteomics *in vitro*

Murine-CMs were exposed to BTZ (300 nM), CFZ (2500 nM) or DMSO for 1 h and proteins were quantified by LC–MS/MS. Briefly, dry pellets were sonicated in lysis buffer (PBS, 5 mM DTT), followed by a 3 min incubation at 95°C with 2% SDS. Reduction was performed with 10 mM DTT for 30 min at 37°C, followed by alkylation with 50 mM iodoacetamide for 30 min in a light-protected environment. Two hundred micrograms of protein were precipitated with chloroform/methanol and digested overnight in 4 μg/ml trypsin (Promega Corporation, Madison, WI, USA). Desalting was performed in Sep-Pak tC18 (200 μg) cartridges (Waters Corporation, Milford, MA, USA). Analysis was performed with the Synapt G2-Si High-Definition Mass Spectrometer (Waters Corporation). The raw files were searched with Progenesis QIP software against the UniProt mouse database. The protein concentrations in the BTZ or CFZ conditions were normalized to those in the DMSO condition to assess global proteome changes between the conditions. P values were calculated using ANOVA and the statistical significance was defined as p value ≤ 0.01. The experiment consisted of 6 biological replicates pooled together into two experimental groups and run in parallel. Raw data as well as analysis is presented in Table S1. A defined cutoff value of a Log2FC = 0.8 was used to differentiate proteins that accumulated or were reduced after treatments. Overrepresentation analysis (ORA) of identified proteins and their classification into the specified processes was performed using WebGestalt online tool and GeneOntology (GO) and network database.^75^

#### *In vivo* treatment and electrocardiography assessment

Mice were injected with the drugs CFZ (16 mg/kg, *i.v.*), BTZ (1 mg/kg, *i.v*.), atRA (1 mg/kg *i.p.*) CFZ and atRA (CFZ: 16 mg/kg *i.v.*, atRA: 1 mg/kg *i.p.*) or vehicle (Captisol, *i.v.*) for 1 h. One hour after drug injection, the mice were anesthetized with ketamine 80 mg/kg intramuscularly and maintained with oral isoflurane 1% during the time of electrocardiogram (ECG) monitoring. The ECGs were recorded with an external ECG monitoring system (IVIS Lumina LT Series III, PerkinElmer, Waltham, MA, USA). ECG analysis and heart rate calculation were performed with an in-house algorithm developed for R Studio (v.3.5.1).

#### Ace2 enzymatic activity evaluation

The *ex vivo* enzymatic activity of Ace2 was determined as described previously^76^ with few modifications. Briefly, BALB/c mice were treated with CFZ or CFZ and atRA for 1 h as described above. Subsequently, the mice were euthanized and proteins from the hearts and lungs were extracted using MagNA Lyser (Roche, Basel, Switzerland) and reaction buffer (1 M NaCl, 0.5 mM ZnCl_2_, 75 mM Tris, pH 7.5). A specific Ace2-quenched fluorogenic substrate (Mca-Ala-Pro-Lys-Dnp)-OH; MedChemExpress, Monmouth Junction, NJ, USA) was added to 20 μg of protein to a final concentration of 30 μM. The measurement was performed for 2 hours at excitation of 320 nm and emission 410 nm using a Tecan Infinite M Plex instrument (Tecan).

#### Label-free proteomic and untargeted metabolomic analysis *in vivo*

BALB/c mice were treated with vehicle, BTZ, CFZ and CFZ + atRA for 1 h as described above. Subsequently, the mice were euthanized. Hearts and plasma were collected. Specifically, blood was collected from murine hearts immediately following euthanasia. After collection, the mice were perfused with 20 ml of PBS containing 2 mM EDTA. The perfusion was carried out by injecting the PBS-EDTA solution into the heart, and then allowing the solution to flow out through the veins via the vena cava. This procedure was repeated until the organs were cleared of blood. Afterwards, the heart has been removed from the animals, snap frozen and stored at -80°C until analysis.

Label-free proteomics was performed on a Q-Exactive HF (Thermo Fisher Scientific) to acquire MS data in data independent acquisition (DIA) mode. Quantification of peptides and proteins was performed using MSstats software packages by BGI Tech Solutions (Hong Kong). A defined cutoff value of a Log2FC = 0.8 and statistical significance of p < 0.05 was used to differentiate proteins that significantly accumulated or were reduced after treatments. Overrepresentation analysis (ORA) of identified proteins and their classification into the specified processes was performed using WebGestalt online tool and GeneOntology (GO) database.^75^

Untargeted metabolomics was performed on a QTOF high-resolution tandem mass spectrometer (Waters Corporation) by BGI Tech Solutions (Hong Kong). To select for differentially expressed metabolites, raw data were filtered by the variable importance in projection (VIP) values of the first two principal components in the PLS-DA model (VIP ≥ 1), fold change (≥ 1.2 or ≤ 0.8) and q-value < 0.005. Enrichment analysis was performed in MetaboAnalyst.^77^

#### Sample preparation and scRNA-seq of murine hearts

BALB/c mice (2 mice per cohort) were treated with vehicle, CFZ or CFZ + atRA for 1 h as described above. Subsequently, the mice were euthanized and perfused with 20 ml of PBS, and small heart tissue pieces were digested three times for 20 min at 37°C with 120 μg/ml collagenase P (Roche) and 25 μg/ml DNase I (Applichem, Darmstadt, Germany) in medium (RPMI-1640 containing 5% FCS (Sigma-Aldrich) and 10 mM HEPES (Sigma-Aldrich). To eliminate remaining erythrocytes, the cell suspensions were incubated with MACS anti-Ter119 microbeads (Miltenyi Biotec) and passed through a MACS LS column (Miltenyi Biotec). Unbound single-cell suspensions were stained with Live/Dead Fixable e780 Aqua staining. Live cells were sorted with a BD FACS Melody cell sorter (BD Biosciences). Sorted single-cell suspensions were run on a 10x Chromium analyzer (10X Genomics, Pleasanton, CA, USA).^78^ cDNA library generation was performed following the established commercial protocol for the Chromium Single Cell 3’ Reagent Kit (v3 Chemistry). Libraries were run on a NovaSeq6000 for Illumina sequencing. A total of 12 samples were processed in two batches, with all conditions present in both batches. Preprocessing and gene expression estimation were performed using CellRanger (v3.0.2)^67^ with Ensembl GRCm38.9 release as reference to build the index. Additional quality control and further analysis were run in R v.3.6.2 using the scater R/Bioconductor package (v1.14.6)^68^ for quality control and the Seurat package (v.3.1.4)^69^ for downstream analysis. First, in each sample, cells with exceedingly high or low numbers of detected genes or uniwue molecular identifier (UMI) counts (> 2.5 median absolute deviations from the overall median) and cells with a large fraction of mitochondrial reads (> 2.5 median absolute deviations above the median fraction) were excluded.

#### ScRNA-seq data analysis

Gene identifiers were retrieved with the org.Mm.eg.db and AnnotationDbi package for R.^70^ Gene Set Enrichment Analysis (GSEA) was performed in WebGestalt.^75^ Gene Ontology (GO) and pathway enrichment analyses were performed by overrepresentation methods with the enrichGo function within the clusterProfiler package for R.^71^ Visualizations were performed with clusterProfiler and enrichPlot.

Differential gene expression analysis was performed using the Seurat wrapper function FindMarkers. The calculation of average Log2 fold change (avg Log2FC) function is based on the Wilcoxon rank sum test, which compares the distribution of expression values for a given gene between two groups or clusters. The avg Log2FC thresholds that are presented were set at 0.25. For visualization, genes with p < 0.01 and avg Log2FC ≥ 0.5 were used.

#### Assessment of MM cell cytotoxicity

Viability of cells was determined after 48h of treatment using Cell Counting Kit (CCK-8) (GK10001, GLP Bio, Montclair, CA, USA) according to manufactures protocol. For the experiments, 1x10^4^ cells/well in 96-well plates were seeded.

### Quantification and statistical analysis

#### Software, statistics and algorithms

For *in vitro* LC–MS/MS, statistical significance was defined as *P* ≤ 0.01 using analysis of variance (ANOVA). For *in vivo* proteomics, statistical significance was defined as *P* ≤ 0.05 using Student’s t test. For scRNA-seq data, statistical significance was defined as *P* ≤ 0.01. A two-sided *P* value ≤ 0.05 was considered to indicate statistical significance in functional *in vitro* assays. The software used in this work is specified in key resources table. Unless otherwise specified, all data were evaluated in R Studio v.3.5.1 (2018-07-02), GraphPad Prism v6 and v8 (GraphPad Software, La Jolla, CA) and MATLAB (MathWorks).

## Supplemental information

Document S1. Figures S1–S7 and Tables S5, S6, S7, and S12

Document S2. Original western blot data

Table S1. LC-MS/MS results from Murine-CMs treated *in vitro* with BTZ, CFZ, and DMSO, related to Figure 2

Table S2. LC-MS/MS results from murine hearts treated with BTZ, CFZ, and vehicle, related to Figure 3

Table S3. LC-MS/MS results from murine hearts treated with CFZ, CFZ and atRA, and vehicle, related to Figure 3

Table S4. Single-cell RNA sequencing marker genes per cluster for cluster identification, related to Figure 4

Table S8. Differentially expressed genes in different populations identified by scRNA-seq after treatment with CFZ vs. vehicle, related to Figure 4

Table S9. Differentially expressed genes in different populations identified by scRNA-seq after treatment with CFZ and atRA vs. CFZ, related to Figure 4

Table S10. Identified metabolites from murine hearts after BTZ, CFZ, or vehicle treatments and comparisons, related to Figure 6

Table S11. Identified metabolites from murine hearts after BTZ, CFZ, or vehicle treatments and comparisons, related to Figure 6
