# Supplementary material for: Carfilzomib-specific proteasome β5/β2 inhibition drives cardiotoxicity via remodeling of protein homeostasis and the renin-angiotensin-system
Source: iScience. 2025 Jul 29;28(9):113228. doi: 10.1016/j.isci.2025.113228 (PMC12392329; doi:10.1016/j.isci.2025.113228)
Supplement: Document S1. Figures S1–S7 and Tables S5, S6, S7, and S12 [file mmc1.pdf]

iScience, Volume *28*

Supplemental information

Carfilzomib-specific proteasome β5/β2 inhibition drives cardiotoxicity via remodeling of protein homeostasis and the renin-angiotensin-system

Max Mendez-Lopez, Andrej Besse, Christian Zuppinger, Christian Perez-Shibayama, Cristina Gil-Cruz, Bogdan I. Florea, Angelina De Martin, Mechthild Lütge, Deborah Beckerova, Simon Klimovic, Xiang Zhou, Leo Rasche, Jan Pribyl, Vladimir Rotrekl, Burkhard Ludewig, Herman S. Overkleeft, Lenka Besse, and Christoph Driessen

SUPPLEMENTAL FIGURES

Figure S1

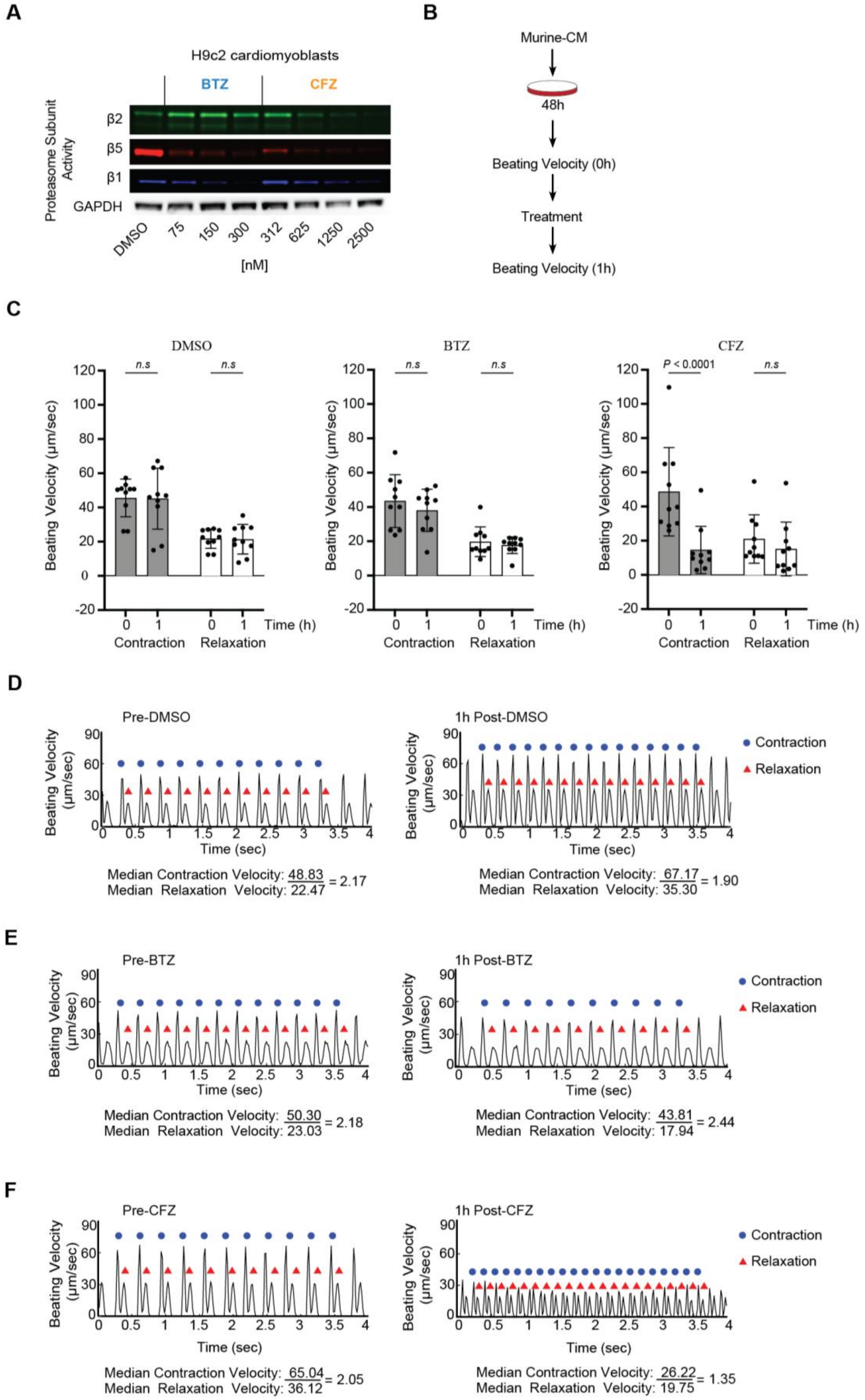

Figure S2

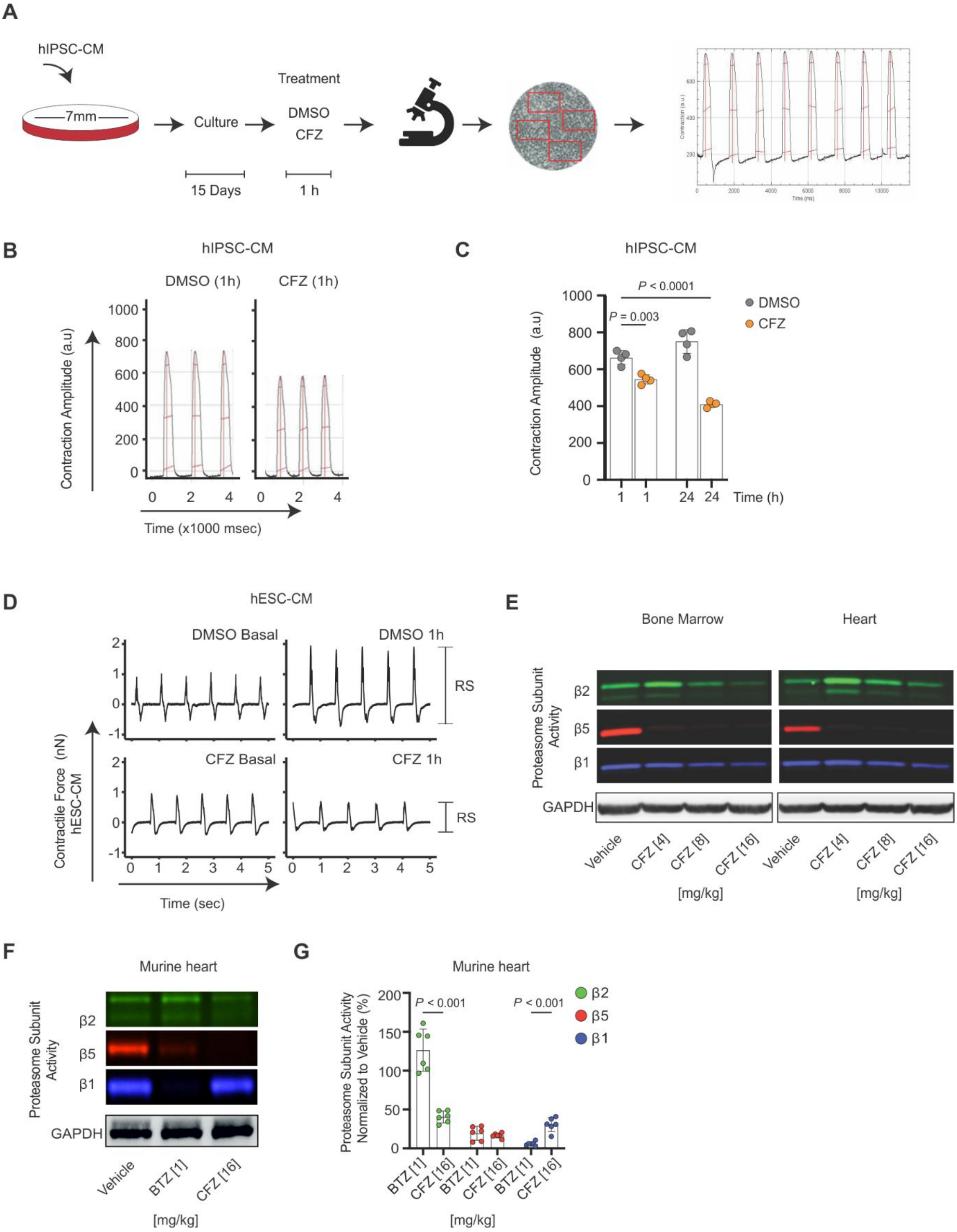
A
hIPSC-CM
7mm
Culture
Treatment
DMSO
CFZ
15 Days
1 h
B
hIPSC-CM
DMSO (1h)
CFZ (1h)
Contraction Amplitude (a.u)
Time (x1000 msec)
C
hIPSC-CM
P < 0.0001
P = 0.003
DMSO
CFZ
Contraction Amplitude (a.u)
Time (h)
D
hESC-CM
DMSO Basal
DMSO 1h
CFZ Basal
CFZ 1h
RS
Contractile Force (nN)
hESC-CM
Time (sec)
E
Bone Marrow
Heart
Proteasome Subunit Activity
β2
β5
β1
GAPDH
Vehicle
CFZ [4]
CFZ [8]
CFZ [16]
[mg/kg]
F
Murine heart
Proteasome Subunit Activity
β2
β5
β1
GAPDH
Vehicle
BTZ [1]
CFZ [16]
[mg/kg]
G
Murine heart
Proteasome Subunit Activity Normalized to Vehicle (%)
P < 0.001
P < 0.001
β2
β5
β1
BTZ [1]
CFZ [16]
[mg/kg]

Figure S3

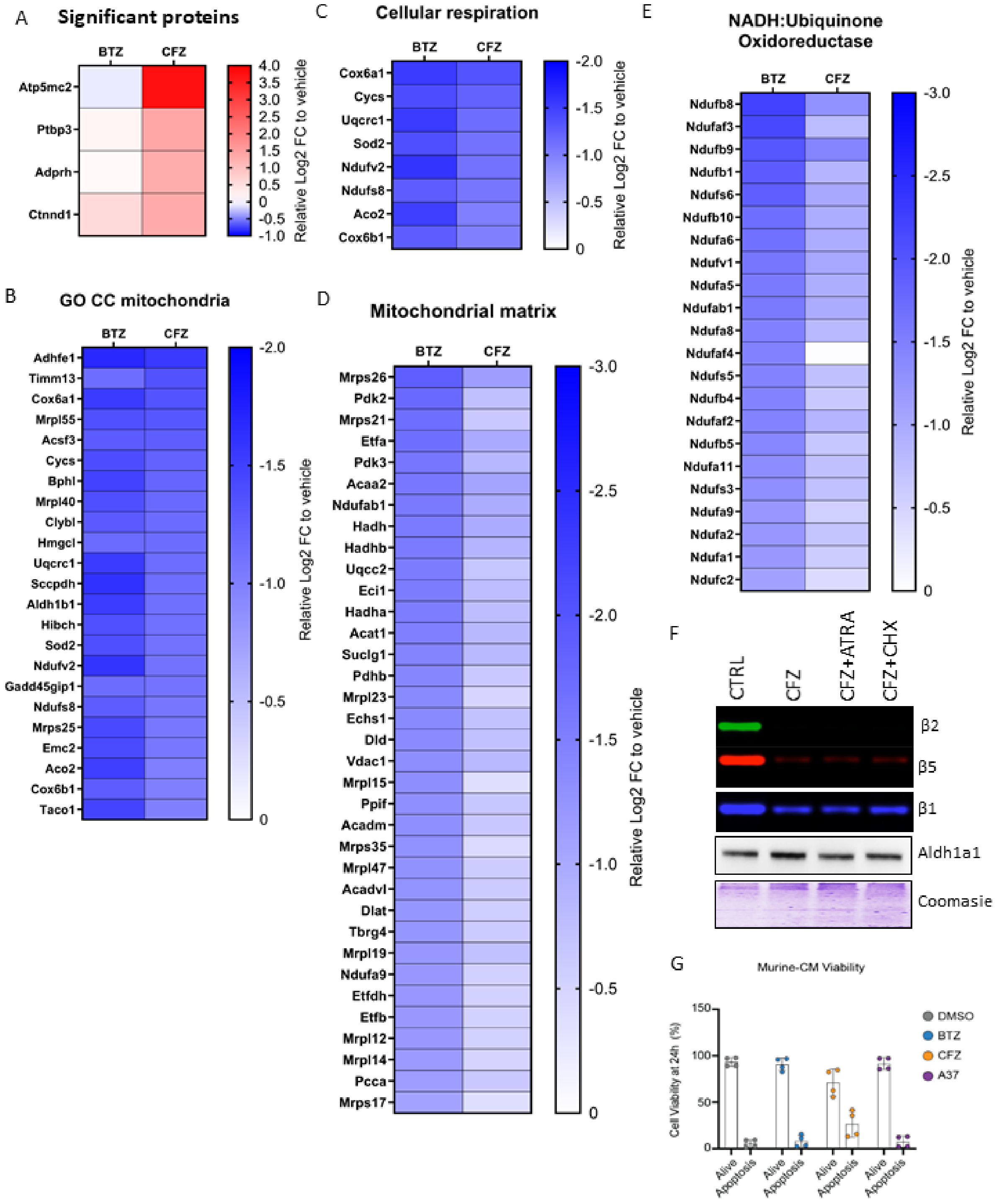
A
Significant proteins
BTZ
CFZ
Atp5mc2
Ptbp3
Adprh
Ctnnd1
Relative Log2 FC to vehicle
B
GO CC mitochondria
C
Cellular respiration
D
Mitochondrial matrix
E
NADH:Ubiquinone Oxidoreductase
F
CTRL
CFZ
CFZ+ATRA
CFZ+CHX
β2
β5
β1
Aldh1a1
Coomasie
G
Murine-CM Viability
Cell Viability at 24h (%)
DMSO
BTZ
CFZ
A37
Alive
Apoptosis

Figure S4

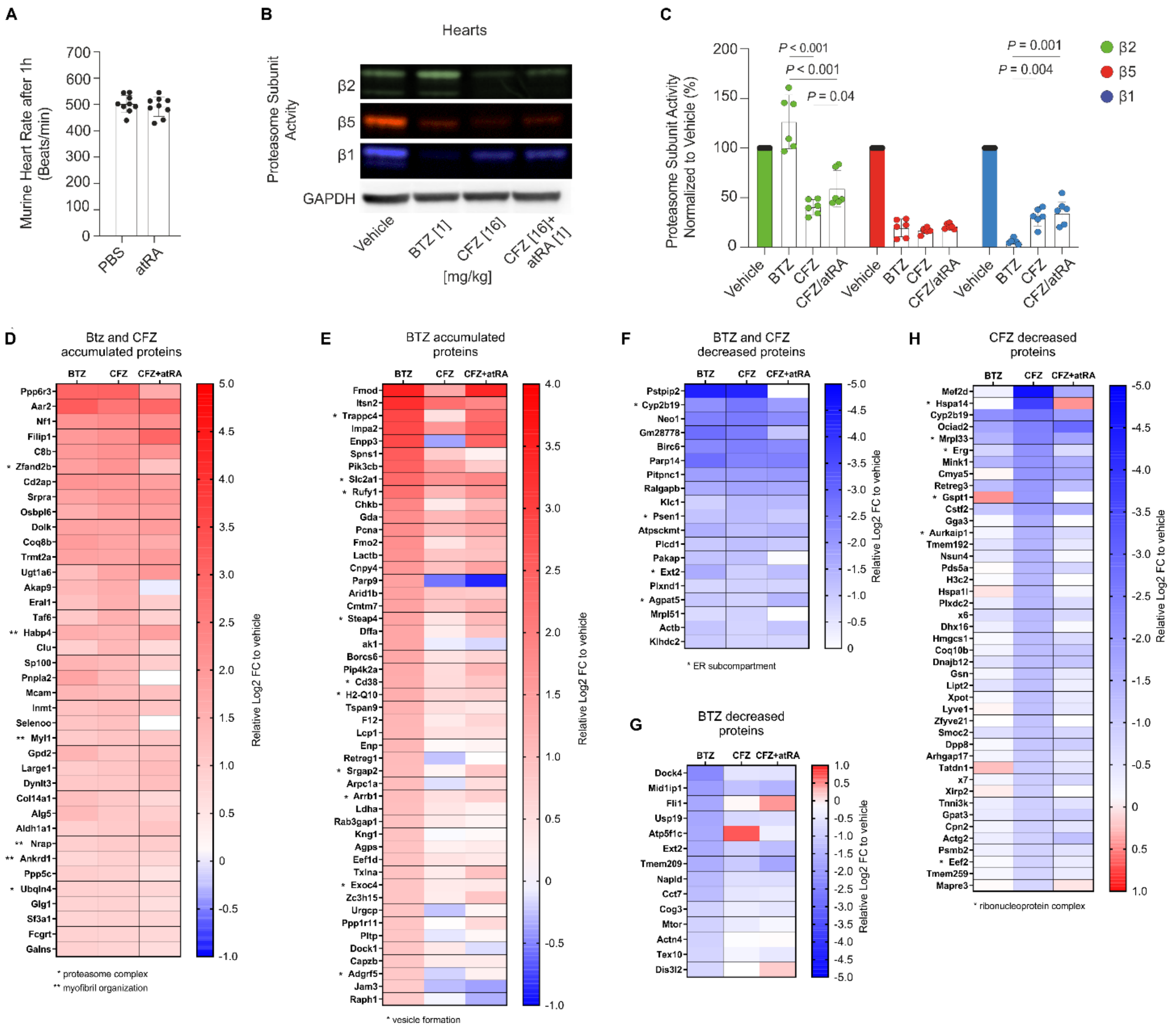
A
Murine Heart Rate after 1h (Beats/min)
PBS
atRA
B
Hearts
Proteasome Subunit Activity
β2
β5
β1
GAPDH
Vehicle
BTZ [1]
CFZ [16]
CFZ [16]+ atRA [1]
[mg/kg]
C
Proteasome Subunit Activity Normalized to Vehicle (%)
P < 0.001
P < 0.001
P = 0.04
P = 0.001
P = 0.004
β2
β5
β1
Vehicle
BTZ
CFZ
CFZ/atRA
D
Btz and CFZ accumulated proteins
Relative Log2 FC to vehicle
* proteasome complex
** myofibril organization
E
BTZ accumulated proteins
* vesicle formation
F
BTZ and CFZ decreased proteins
* ER subcompartment
G
BTZ decreased proteins
H
CFZ decreased proteins
* ribonucleoprotein complex

Figure S5

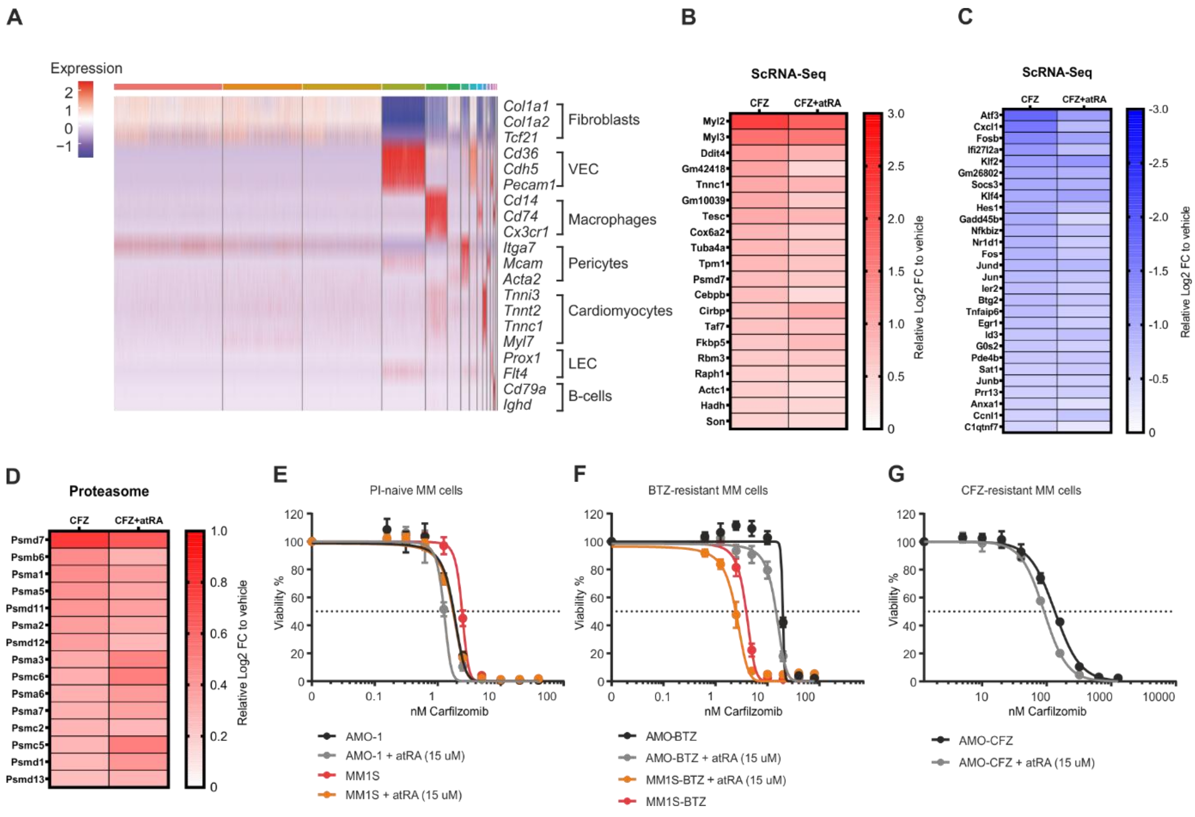
A
Expression
Col1a1
Col1a2
Tcf21
Fibroblasts
Cd36
Cdh5
Pecam1
VEC
Cd14
Cd74
Cx3cr1
Macrophages
Itga7
Mcam
Acta2
Pericytes
Tnni3
Tnnt2
Tnnc1
Myl7
Cardiomyocytes
Prox1
Flt4
LEC
Cd79a
Ighd
B-cells
B
ScRNA-Seq
CFZ
CFZ+atRA
Relative Log2 FC to vehicle
C
ScRNA-Seq
CFZ
CFZ+atRA
Relative Log2 FC to vehicle
D
Proteasome
CFZ
CFZ+atRA
Relative Log2 FC to vehicle
E
PI-naive MM cells
Viability %
nM Carfilzomib
AMO-1
AMO-1 + atRA (15 uM)
MM1S
MM1S + atRA (15 uM)
F
BTZ-resistant MM cells
Viability %
nM Carfilzomib
AMO-BTZ
AMO-BTZ + atRA (15 uM)
MM1S-BTZ + atRA (15 uM)
MM1S-BTZ
G
CFZ-resistant MM cells
Viability %
nM Carfilzomib
AMO-CFZ
AMO-CFZ + atRA (15 uM)

Figure S6

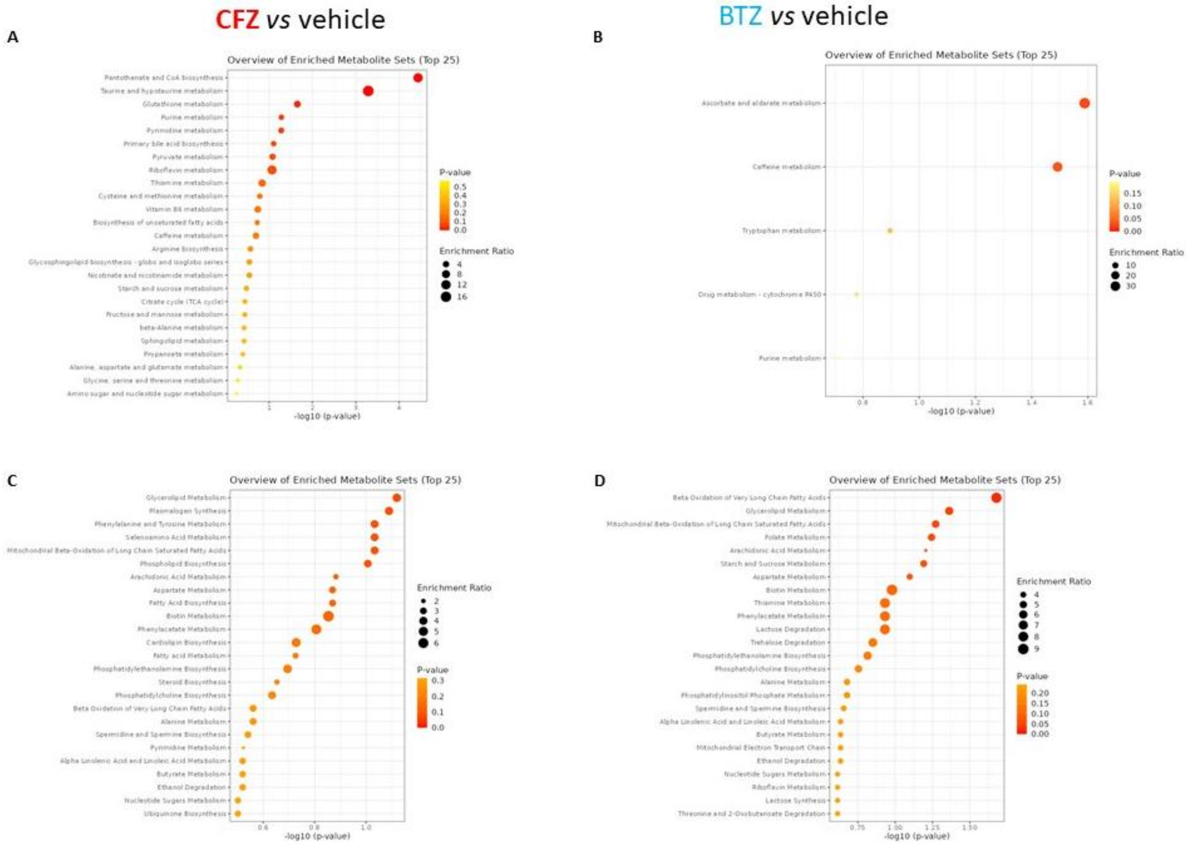

CFZ vs vehicle
BTZ vs vehicle
A
B
C
D
Overview of Enriched Metabolite Sets (Top 25)
P-value
Enrichment Ratio
-log10 (p-value)

Figure S7

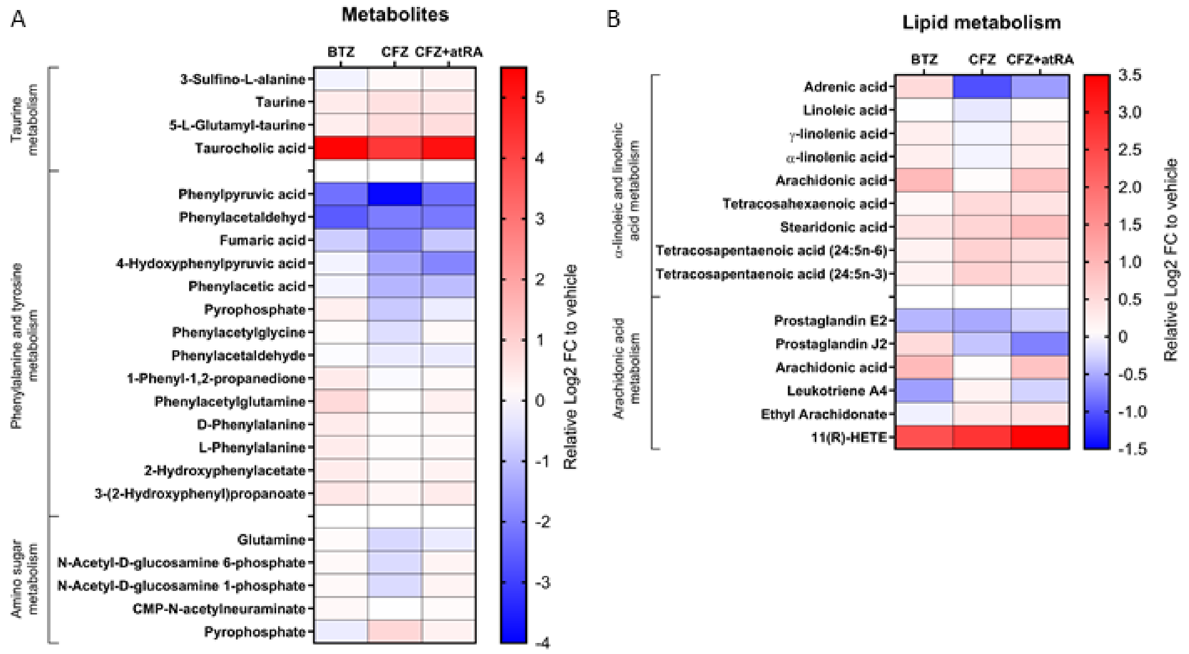

A
Metabolites
BTZ
CFZ
CFZ+atRA
Taurine metabolism
3-Sulfino-L-alanine
Taurine
5-L-Glutamyl-taurine
Taurocholic acid
Phenylalanine and tyrosine metabolism
Phenylpyruvic acid
Phenylacetaldehyd
Fumaric acid
4-Hydoxyphenylpyruvic acid
Phenylacetic acid
Pyrophosphate
Phenylacetylglycine
Phenylacetaldehyde
1-Phenyl-1,2-propanedione
Phenylacetylglutamine
D-Phenylalanine
L-Phenylalanine
2-Hydroxyphenylacetate
3-(2-Hydroxyphenyl)propanoate
Amino sugar metabolism
Glutamine
N-Acetyl-D-glucosamine 6-phosphate
N-Acetyl-D-glucosamine 1-phosphate
CMP-N-acetylneuraminate
Pyrophosphate
5
4
3
2
1
0
-1
-2
-3
-4
Relative Log2 FC to vehicle
B
Lipid metabolism
BTZ
CFZ
CFZ+atRA
α-linoleic and linolenic acid metabolism
Adrenic acid
Linoleic acid
γ-linolenic acid
α-linolenic acid
Arachidonic acid
Tetracosahexaenoic acid
Stearidonic acid
Tetracosapentaenoic acid (24:5n-6)
Tetracosapentaenoic acid (24:5n-3)
Arachidonic acid metabolism
Prostaglandin E2
Prostaglandin J2
Arachidonic acid
Leukotriene A4
Ethyl Arachidonate
11(R)-HETE
3.5
3.0
2.5
2.0
1.5
1.0
0.5
0
-0.5
-1.0
-1.5
Relative Log2 FC to vehicle

SUPPLEMENTAL FIGURE LEGENDS

Supplemental Figure S1: CFZ-mediated proteasome inhibition impairs cardiomyocyte contractility in different rodent models. Related to Figure 1.

(A) Representative images of dose-dependent proteasome subunit-specific inhibitory effect after 1 h pulse treatment with BTZ or CFZ at the indicated doses in rat H9c2 cardiomyoblasts, as measured by activity-based proteasome probes. (B) Experimental design of motion vector measurements in Murine-CMs. (C) Beating velocity measured before (0 h) and after (1 h) treatment with the respective compounds (BTZ = 300 nM, CFZ = 2500 nM). Each value represents an individual technical replicate; the data represent the mean ± SD (*n* = 5 independent experiments in duplicate). (D-F) Normalized beating velocity for contraction and relaxation in Murine-CMs before (pre) and 1h after (post) treatment with DMSO, BTZ (300 nM), or CFZ (2500 nM). Statistical significance was tested using two-way ANOVA and Bonferroni test for multiple comparisons. A p-value < 0.05 is considered statistically significant.

BTZ: bortezomib; CFZ: carfilzomib; DMSO: dimethyl sulfoxide; Murine-CMs: murine neonatal cardiomyocytes.

Figure S2: CFZ-specific β5 and β2 proteasome inhibition alters contractility in various models of cardiomyocytes. Related to Figure 1.

(A) Experimental design for motion vector measurements in hiPSC-CMs. (**B)** Representative images of the contraction amplitude of hiPSC-CMs after 1 h pulse treatment with DMSO or CFZ (2500 nM). **(C)** Effects of DMSO or CFZ (2500 nM)-mediated proteasome inhibition on contraction amplitude in hiPSC-CMs after 24 h; the data represent the mean ± SD (*n* = 4 independent experiments in duplicate). **(D)** Representative example of changes in the RS amplitude after acute CFZ (2500 nM) treatment in hESC-CMs. **(E)** Representative example of activity-based proteasome probe labelling showing proteasome subunit activity in murine bone marrow cells and hearts after dose titration with CFZ. **(F)** Example of activity-based proteasome probe labelling showing BTZ-type β5 and β1 subunit inhibition (1 mg/kg) and CFZ-type β5 and β2 subunit inhibition (16 mg/kg) in murine hearts after 1 h *i.v.* treatment and **(G)** its quantification; the data represent the mean ± SD (*n* = 6 mice). Statistical significance was tested with two-way ANOVA and Bonferroni correction for multiple comparisons. A p-value $<$ 0.05 is considered statistically significant.

BTZ: bortezomib; CFZ: carfilzomib; DMSO: dimethyl sulfoxide; Murine-CMs: murine neonatal cardiomyocytes; hESC-CMs: human embryonic stem cell-derived cardiomyocytes; hiPSC-CMs: human induced pluripotent stem cell-derived cardiomyocytes.

Figure S3: CFZ-mediated proteasome inhibition rapidly alters the Murine-CM proteome, with significant depletion of proteins involved in mitochondrial respiration. Related to Figure 2.

Heatmaps depicting proteins in Murine-CM deregulated 1 h after treatment with BTZ (300 nM) and CFZ (2500 nM) *in vitro* and normalized to vehicle (DMSO) treatment (*n* = 3 biological replicates). **(A)** Significantly accumulated proteins after treatment with CFZ, but not BTZ (Log2FC for CFZ ≥ 1; Log2FC_CFZ - Log2FC_BTZ ≥ 0.5; $p < 0.01$). **(B)** Significantly reduced proteins after treatment with BTZ and CFZ (Log2FC for BTZ ≤ -1; Log2FC for CFZ ≤ -1; Log2FC_BTZ - Log2FC_CFZ ≤ 0.5; $p < 0.01$) mapped to the mitochondria based on GO CC. **(C)** Significantly reduced proteins after treatment with BTZ and CFZ (Log2FC for BTZ ≤ -1; Log2FC for CFZ ≤ -1; Log2FC_BTZ - Log2FC_CFZ ≤ 0.5; $p < 0.01$) mapped to cellular respiration based on GO BP. **(D)** Significantly reduced proteins after treatment with BTZ, but not CFZ (Log2FC for BTZ ≤ -1; Log2FC_BTZ - Log2FC_CFZ ≥ 0.5; $p < 0.01$) mapped to the mitochondrial matrix, based on GO CC. **(E)** Significantly depleted proteins after treatment with BTZ, but not CFZ (Log2FC for BTZ ≤ -1; Log2FC_BTZ - Log2FC_CFZ ≥ 0.5; $p < 0.01$), belonging to mitochondrial NADH:Ubiquinone Oxidoreductases that form the respiratory chain. **(F)** Immunoblot of Aldh1a1 in murine-CMs after treatment for 1 h with CFZ (2500 nM) CFZ + ATRA (15μM) or CFZ with CHX;Coomasie served as a control of loading. **(G)** Murine-CMs viability after treatment for 1 h with BTZ (300 nM), CFZ (2500 nM) or A37 (6μM); the data represent the mean ± SD (*n* = 4 independent experiments).

ATRA: All-trans retinoic acid; BTZ: bortezomib; BP: biological process; CC: cellular component; CFZ: carfilzomib; CHX: cycloheximide; DMSO: dimethyl sulfoxide; FC: fold change; GO: gene ontology; Murine-CMs: murine neonatal cardiomyocytes

Figure S4: CFZ-mediated proteasome inhibition *in vivo* alters the proteome of murine hearts, leading to a significant depletion of proteins involved in cellular translation and chaperoning. Related to Figure 3.

(A) Murine heart rate after 1 h of *i.v.* treatment with PBS (vehicle for atRA) or atRA (1 mg/kg), the data represent the mean ± SD (*n* = 9 mice). **(B)** Representative example of activity-based proteasome probe labelling showing proteasome subunit activity in murine hearts after the treatment for 1 h with BTZ (1 mg/kg), CFZ (16 mg/kg) or CFZ + atRA (16 mg/kg and 1 mg/kg, respectively). **(C)** Quantification of proteasome subunit inhibition at the end of 1 h treatment with BTZ (1 mg/kg), CFZ (16 mg/kg), or CFZ + atRA (16 mg/kg and 1 mg/kg, respectively); the data represent the mean ± SD (*n* = 6 mice). Statistical significance was tested with two-way ANOVA and Bonferroni correction for multiple comparisons. A p-value < 0.05 is considered statistically significant. **(D-H)** Heatmaps depicting proteins in murine hearts

deregulated 1 h after treatment with BTZ (1 mg/kg), CFZ (16 mg/kg) and CFZ+atRA and normalized to vehicle (Captisol) treatment (*n* = 4 mice per cohort). **(D)** Significantly accumulated proteins after treatment with BTZ and CFZ (Log2FC ≥ 0.8 for BTZ and CFZ; Log2FC_BTZ - Log2FC_CFZ ≤ 0.5; $p < 0.05$), with stars are indicated proteins belonging to proteasome complex and myofibril organization, based on GO CC. **(E)** Significantly accumulated proteins after treatment with BTZ, but not CFZ (Log2FC for BTZ ≥ 0.8; Log2FC_BTZ - Log2FC_CFZ ≥ 0.5; $p < 0.05$), with stars are indicated proteins belonging to vesicle formation, based on GO CC. **(F)** Significantly depleted proteins after treatment with BTZ and CFZ (Log2FC ≤ -0.8 for BTZ and CFZ; Log2FC_BTZ - Log2FC_CFZ ≤ 0.5; $p < 0.05$), with stars are indicated proteins belonging to ER sub-compartment, based on GO CC. **(G)** Significantly depleted proteins after treatment with BTZ, but not CFZ (Log2FC for BTZ ≤ -0.8; Log2FC_BTZ - Log2FC_CFZ ≥ 0.5; $p < 0.05$). **(H)** Significantly depleted proteins after treatment with CFZ, but not BTZ (Log2FC for CFZ ≤ -0.8; Log2FC_CFZ - Log2FC_BTZ ≥ 0.5; $p < 0.05$), with stars are indicated proteins belonging to the ER sub-compartment, based on GO CC.

atRA: all-trans retinoic acid; BTZ: bortezomib; CC: cellular component; CFZ: carfilzomib; FC: fold change; GO: gene ontology

Figure S5: CFZ-associated changes in murine cardiomyocytes *in vivo* and the effect of atRA on CFZ-specific gene expression changes. Related to Figure 4 and Figure 5.

(A) Cluster-specific marker gene identification of cellular subtypes in murine hearts treated with vehicle (*n* = 2 mice). **(B-D)** Heatmaps representing significantly deregulated gene expression in cardiomyocytes of CFZ (16 mg/kg) and CFZ + atRA (16 mg/kg and 1 mg/kg, respectively) treated mice. The data are presented as a Log2 FC of a condition related to vehicle treatment (*n* = 2 mice) and statistical differences were tested with a *t* test. (B) Heatmap representing gene expression significantly induced by CFZ and values for CFZ + atRA cotreatment. (C) Heatmap representing gene expression significantly downregulated by CFZ and values for CFZ + atRA cotreatment. (D) Heatmap representing gene expression of proteasome-related genes induced by CFZ and values for CFZ + atRA cotreatment. **(E-G)** Dose-response curves of MM cell lines to increasing doses of CFZ in monotherapy or in combination with a single dose of atRA (15μM) in (E) PI-naïve cells, (F) BTZ-resistant cells, (G) CFZ-resistant cells; the data represent the mean ± SD (*n* = 2 independent experiments in triplicate).

atRA: all-trans retinoic acid; BTZ: bortezomib; CFZ: carfilzomib; FC: fold change; MM: multiple myeloma; PBS: phosphate buffered saline

Figure S6: Enrichment analysis of the deregulated metabolites after PI treatment and their classification using the KEGG database. Related to Figure 6.

KEGG pathway analysis was performed to visualize metabolic pathways deregulated 1 h after treatment with BTZ (1 mg/kg) or CFZ (16 mg/kg) *in vivo* and normalized to vehicle treatment (n = 3 mice per cohort). **(A)** Dot-plot visualizing enriched metabolic pathways for metabolites decreased after CFZ (16 mg/kg) treatment, after normalization to vehicle treatment. **(B)** Dot-plot visualizing enriched metabolic pathways for metabolites decreased after BTZ (1 mg/kg) treatment, after normalization to vehicle treatment. **(C)** Dot-plot visualizing enriched metabolic pathways for metabolites increased after CFZ (16 mg/kg) treatment, after normalization to vehicle treatment. **(D)** Dot-plot visualizing enriched metabolic pathways for metabolites increased after BTZ (1 mg/kg) treatment, after normalization to vehicle treatment.

BTZ: bortezomib; CFZ: carfilzomib; KEGG: Kyoto encyclopedia of genes and genome.

Figure S7: Circulating metabolites in murine plasma. Related to Figure 6.

(A-B) Heatmaps depicting circulating metabolites in murine plasma deregulated 1 h after treatment with BTZ (1 mg/kg), CFZ (16 mg/kg) and CFZ + atRA (16 mg/kg and 1 mg/kg, respectively) *in vivo* and normalized to vehicle treatment (n = 3 mice per cohort). (A) Deregulated metabolites identified from the metabolism of taurine, phenylalanine and tyrosine and amino sugars. (B) Deregulated metabolites identified from the metabolism of α-linoleic and linolenic acid and from arachidonic acid. Data are presented as Log2 FC of treatment relative to vehicle treatment.

atRA: all-trans retinoic acid; BTZ: bortezomib; CFZ: carfilzomib; FC: fold change.

SUPPLEMENTAL TABLES

Table S5: Number of cells used for single-cell RNA analysis. Related to Figure 4.

Cell population	Total number of cells analyzed
**fibroblasts**	33797
**endothelial cells**	5056
**macrophages**	2418
**pericytes**	910
**cardiomyocytes**	378

Table S6: GSEA and TF network analysis in cardiomyocytes *in vivo*: CFZ *vs.* vehicle.

Related to Figure 4.

Gene Set	ES	NES	FDR	size	LEN
CEBPDELTA_Q6	-0.5157	-2.3541	0.0617	23	7
IRF1_01	-0.4623	-2.2728	0.0647	29	19
CREB_Q2	-0.3739	-2.1067	0.0818	44	16
MEIS1_01	-0.4307	-2.0646	0.0808	27	15
RGAANNTTC_HSF1_01	-0.3319	-2.0498	0.0821	60	41
FAC1_01	-0.4177	-2.0425	0.0798	29	25
AP4_Q6	-0.4903	-2.0119	0.0829	18	12
NKX61_01	-0.4469	-1.9953	0.0820	22	16
WHN_B	-0.4136	-1.9949	0.0780	28	14
TGTTTGY_HNF3_Q6	-0.2948	-1.9847	0.0794	82	65
TGANNYRGCA_TCF11MAFG_01	0.3292	1.9858	0.0888	47	15
AR_01	0.4800	1.9912	0.0939	18	8
MMEF2_Q6	0.3687	2.0187	0.0844	37	8
SREBP1_Q6	0.4233	2.0594	0.0710	26	8
MEF2_Q6_01	0.3837	2.0843	0.0702	37	11
MEF2_02	0.3958	2.1049	0.0726	34	12
TAAWWATAG_RSRFC4_Q2 (MEF2A)	0.4304	2.1557	0.0606	30	14
AMEF2_Q6	0.4164	2.2395	0.0377	37	10
RSRFC4_Q2 (MEF2A)	0.4701	2.3090	0.0329	28	11
RSRFC4_01 (MEF2A)	0.4481	2.3589	0.0443	33	15

GeneSet: ID of the gene set.

ES: Enrichment score

NES: Enrichment score normalized to average ES of all permutations.

FDR: p value corrected for multiple testing.

size: Number of genes in the set after filtering.

LEN: Leading Edge Number. Number of genes in the leading edge.

In yellow are marked positively enriched Gene sets that reached statistical significance based on FDR < 0.05.

Table S7: Differentially expressed genes classified based on GSEA GO BP, in different populations identified by scRNA-seq after treatment with CFZ *vs.* vehicle. Related to Figure 4.

Cardiomyocytes						
**Gene Set**	**Description**	**ES**	**NES**	**FDR**	**size**	**LEN**
GO:0002237	response to molecule of bacterial origin	-0.4885	-2.5314	0.0038	33	14
GO:0007178	transmembrane receptor protein serine/threonine kinase signaling pathway	-0.5126	-2.5144	0.0032	27	16
GO:0014074	response to purine-containing compound	-0.7000	-2.4076	0.0060	11	6
GO:0046683	response to organophosphorus	-0.7000	-2.4076	0.0060	11	6
GO:0043620	regulation of DNA-templated transcription in response to stress	-0.5824	-2.2882	0.0174	16	6
GO:0007389	pattern specification process	-0.4621	-2.2639	0.0181	28	16
GO:0048511	rhythmic process	-0.4450	-2.1602	0.0409	30	9
GO:0071241	cellular response to inorganic substance	-0.5411	-2.1557	0.0379	16	7
GO:0009612	response to mechanical stimulus	-0.4404	-2.1426	0.0379	27	15
GO:0050727	regulation of inflammatory response	-0.4911	-2.1389	0.0350	22	9
GO:0043462	regulation of ATPase activity	0.5937	2.0485	0.0397	11	5
GO:0003007	heart morphogenesis	0.4028	2.0489	0.0435	30	7
GO:0072521	purine-containing compound metabolic process	0.3364	2.2059	0.0141	67	32
GO:0009123	nucleoside monophosphate metabolic process	0.3671	2.2476	0.0110	54	27
GO:0019693	ribose phosphate metabolic process	0.3523	2.2721	0.0098	65	32
GO:0010927	cellular component assembly involved in morphogenesis	0.6286	2.2758	0.0117	13	8
GO:0003012	muscle system process	0.4160	2.2984	0.0116	41	13
GO:0003013	circulatory system process	0.4187	2.3293	0.0111	37	9
GO:0009141	nucleoside triphosphate metabolic process	0.3860	2.3635	0.0124	53	27
GO:0031032	actomyosin structure organization	0.6335	2.5416	0.0057	16	7
**Endothelial cells**						
**Gene Set**	**Description**	**ES**	**NES**	**FDR**	**size**	**LEN**
GO:0046683	response to organophosphorus	-0.6731	-2.3696	0.0162	12	6
GO:0014074	response to purine-containing compound	-0.6397	-2.3257	0.0135	13	6
GO:0048568	embryonic organ development	-0.5220	-2.1778	0.0268	19	10
GO:0048511	rhythmic process	-0.5326	-2.1177	0.0388	16	7
GO:0030099	myeloid cell differentiation	-0.4875	-2.0225	0.0751	19	11
GO:0010038	response to metal ion	-0.4306	-1.9559	0.1123	23	9
GO:0043620	regulation of DNA-templated transcription in response to stress	-0.6516	-1.9425	0.1071	8	5
GO:0006260	DNA replication	-0.7225	-1.8925	0.1372	6	5
GO:0071241	cellular response to inorganic substance	-0.5158	-1.8911	0.1238	14	6
GO:0071559	response to transforming growth factor beta	-0.5045	-1.8726	0.1276	13	10
GO:0022613	ribonucleoprotein complex biogenesis	0.3377	1.5080	0.5213	24	18
GO:0051186	cofactor metabolic process	0.4986	1.5225	0.5225	8	3

GO:0072503	cellular divalent inorganic cation homeostasis	0.4003	1.6544	0.5048	19	7
GO:0042594	response to starvation	0.4385	1.7073	0.4343	16	8
GO:0072524	pyridine-containing compound metabolic process	0.6719	1.7110	0.4627	5	3
GO:0001764	neuron migration	0.5945	1.7127	0.5041	7	5
GO:0002181	cytoplasmic translation	0.6120	1.7305	0.5025	7	7
GO:0097193	intrinsic apoptotic signaling pathway	0.3905	1.7594	0.4743	24	7
GO:0060560	developmental growth involved in morphogenesis	0.4951	1.7749	0.4885	13	8
GO:0006959	humoral immune response	0.6782	1.7969	0.4938	6	4
**Pericytes**						
**Gene Set**	**Description**	**ES**	**NES**	**FDR**	**size**	**LEN**
GO:0044706	multi-multicellular organism process	-0.6870	-2.5991	0.0038	15	14
GO:1903706	regulation of hemopoiesis	-0.5153	-2.5205	0.0026	29	15
GO:0019221	cytokine-mediated signaling pathway	-0.7330	-2.4845	0.0034	11	6
GO:0097305	response to alcohol	-0.7050	-2.4326	0.0051	11	6
GO:0019216	regulation of lipid metabolic process	-0.5780	-2.4042	0.0049	19	9
GO:0048511	rhythmic process	-0.5242	-2.2899	0.0149	21	10
GO:0009612	response to mechanical stimulus	-0.5359	-2.2511	0.0172	19	9
GO:0002237	response to molecule of bacterial origin	-0.5660	-2.2049	0.0214	16	8
GO:0034612	response to tumor necrosis factor	-0.6311	-2.1689	0.0279	11	5
GO:0042110	T cell activation	-0.4701	-2.1123	0.0370	22	12
GO:0061919	process utilizing autophagic mechanism	0.4327	1.6699	0.3479	14	12
GO:0007015	actin filament organization	0.3816	1.7014	0.3127	22	7
GO:0015698	inorganic anion transport	0.6309	1.7098	0.3213	6	4
GO:0008380	RNA splicing	0.4019	1.7148	0.3383	18	15
GO:0031032	actomyosin structure organization	0.5366	1.7442	0.3481	9	4
GO:0015893	drug transport	0.7046	1.7967	0.3287	5	2
GO:0071826	ribonucleoprotein complex subunit organization	0.5377	1.9447	0.2000	13	12
GO:0006970	response to osmotic stress	0.6161	1.9653	0.2266	9	6
GO:0006413	translational initiation	0.6619	2.1394	0.0760	9	7
GO:0002181	cytoplasmic translation	0.8514	2.1764	0.1144	5	5
**Macrophages**						
**Gene Set**	**Description**	**ES**	**NES**	**FDR**	**size**	**LEN**
GO:0002237	response to molecule of bacterial origin	-0.6540	-2.6036	0.0000	28	20
GO:0009612	response to mechanical stimulus	-0.8357	-2.5293	0.0000	12	11
GO:0050900	leukocyte migration	-0.6721	-2.4983	0.0000	22	11
GO:0019221	cytokine-mediated signaling pathway	-0.6660	-2.4607	0.0003	22	14
GO:0070555	response to interleukin-1	-0.7676	-2.4110	0.0002	12	9
GO:0034612	response to tumor necrosis factor	-0.7254	-2.3649	0.0008	16	12
GO:0097305	response to alcohol	-0.7334	-2.3333	0.0008	14	12
GO:1990868	response to chemokine	-0.7288	-2.3281	0.0007	13	8
GO:0071216	cellular response to biotic stimulus	-0.6211	-2.3059	0.0007	22	15
GO:0070371	ERK1 and ERK2 cascade	-0.6737	-2.2977	0.0008	18	12
GO:0022411	cellular component disassembly	0.4038	1.2355	0.5281	8	3
GO:0032409	regulation of transporter activity	0.5192	1.2854	0.5182	5	5
GO:0006457	protein folding	0.5496	1.3404	0.5089	5	2

GO:0031032	actomyosin structure organization	0.5058	1.5476	0.5310	8	8
GO:0008380	RNA splicing	0.5238	1.6282	0.4129	8	6
GO:0051186	cofactor metabolic process	0.4937	1.7783	0.2128	12	8
GO:0006397	mRNA processing	0.6241	1.8032	0.2300	7	6
GO:0022613	ribonucleoprotein complex biogenesis	0.6809	2.0443	0.0526	8	8
GO:0055076	transition metal ion homeostasis	0.8385	2.0820	0.0572	5	5
GO:0071826	ribonucleoprotein complex subunit organization	0.6836	2.1518	0.0606	9	9
**Fibroblasts**						
**Gene Set**	**Description**	**ES**	**NES**	**FDR**	**size**	**LEN**
GO:0097305	response to alcohol	-0.8269	-2.3449	0.0023	9	6
GO:0048511	rhythmic process	-0.5970	-2.2415	0.0075	18	13
GO:0009612	response to mechanical stimulus	-0.6951	-2.1513	0.0142	11	8
GO:0034612	response to tumor necrosis factor	-0.7729	-2.1418	0.0121	8	7
GO:0014074	response to purine-containing compound	-0.7268	-2.1063	0.0125	9	8
GO:0046683	response to organophosphorus	-0.7268	-2.1063	0.0125	9	8
GO:0002237	response to molecule of bacterial origin	-0.5953	-2.0451	0.0211	15	9
GO:0019221	cytokine-mediated signaling pathway	-0.7168	-1.9868	0.0351	8	7
GO:0002697	regulation of immune effector process	-0.6430	-1.8883	0.0818	9	6
GO:0070555	response to interleukin-1	-0.6309	-1.7377	0.1730	8	7
GO:0009141	nucleoside triphosphate metabolic process	0.3194	0.9083	0.8631	7	1
GO:0090407	organophosphate biosynthetic process	0.3559	0.9221	0.8488	6	1
GO:0019439	aromatic compound catabolic process	0.2817	0.9240	0.8594	10	2
GO:0046700	heterocycle catabolic process	0.2817	0.9240	0.8594	10	2
GO:1901361	organic cyclic compound catabolic process	0.2817	0.9240	0.8594	10	2
GO:0070661	leukocyte proliferation	0.3182	0.9920	0.8526	9	2
GO:0006397	mRNA processing	0.3742	0.9928	0.8684	6	2
GO:0032963	collagen metabolic process	0.4065	1.0012	0.8667	5	1
GO:0045926	negative regulation of growth	0.5530	1.8068	0.5536	11	5
GO:0071826	ribonucleoprotein complex subunit organization	0.6330	1.8541	0.7624	8	8

GeneSet: ID of the gene set.

ES: Enrichment score

NES: Enrichment score normalized to average ES of all permutations.

FDR: p value corrected for multiple testing.

size: Number of genes in the set after filtering.

LEN: Leading Edge Number. Number of genes in the leading edge.

In yellow are marked positively enriched Gene sets that reached statistical significance based on FDR < 0.05.

In green are marked negatively enriched Gene sets that reached statistical significance based on FDR < 0.05.

Table S12: Basic characteristics of MM patients. Related to STAR Methods

Age	Sex	Carfilzomib Dose (mg/m^2, iv)	Response Status (IMWG)	Disease Subtype	Line of therapy
66	M	20	PD	IgG Kappa	3
78	M	27	PR	IgG Lambda	4
77	M	27	PD	LC Lambda	3
47	F	36	PD	IgG Lambda	12
79	M	36	PR	IgM Lambda	4
62	M	56	SD	IgG Kappa	13

F: female; M: male; PD: progressive disease; PR: partial remission; SD: stable disease
