# Supplementary material for: Carfilzomib-specific proteasome β5/β2 inhibition drives cardiotoxicity via remodeling of protein homeostasis and the renin-angiotensin-system
Source: iScience. 2025 Jul 29;28(9):113228. doi: 10.1016/j.isci.2025.113228 (PMC12392329; doi:10.1016/j.isci.2025.113228)

Carfilzomib specific proteasome β5 and β2 inhibition drives cardiotoxicity via remodeling of protein homeostasis and renin-angiotensin-system

Original western blot data

Figure 1A

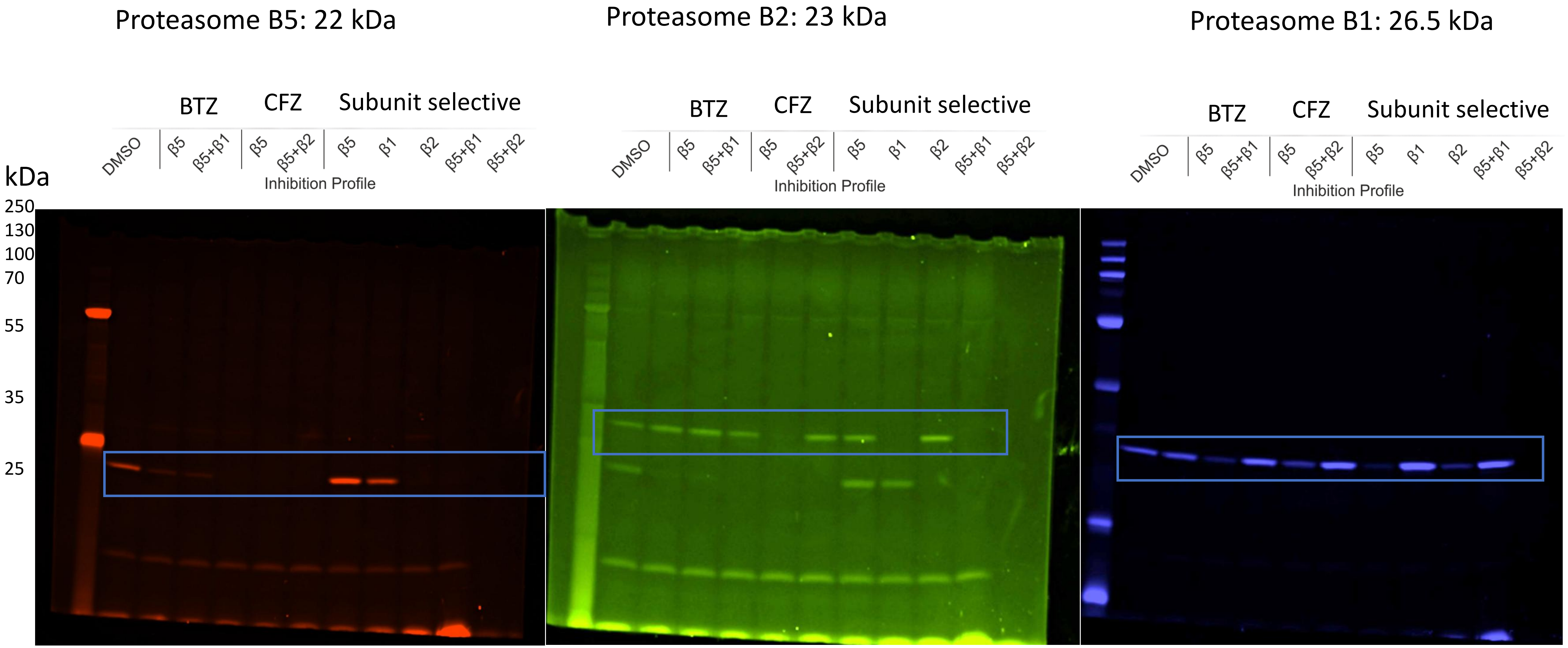

Proteasome B5: 22 kDa
Proteasome B2: 23 kDa
Proteasome B1: 26.5 kDa
BTZ
CFZ
Subunit selective
DMSO
β5
β5+β1
β5
β5+β2
β5
β1
β2
β5+β1
β5+β2
Inhibition Profile
kDa
250
130
100
70
55
35
25

Figure 1A

GAPDH: 37 kDa

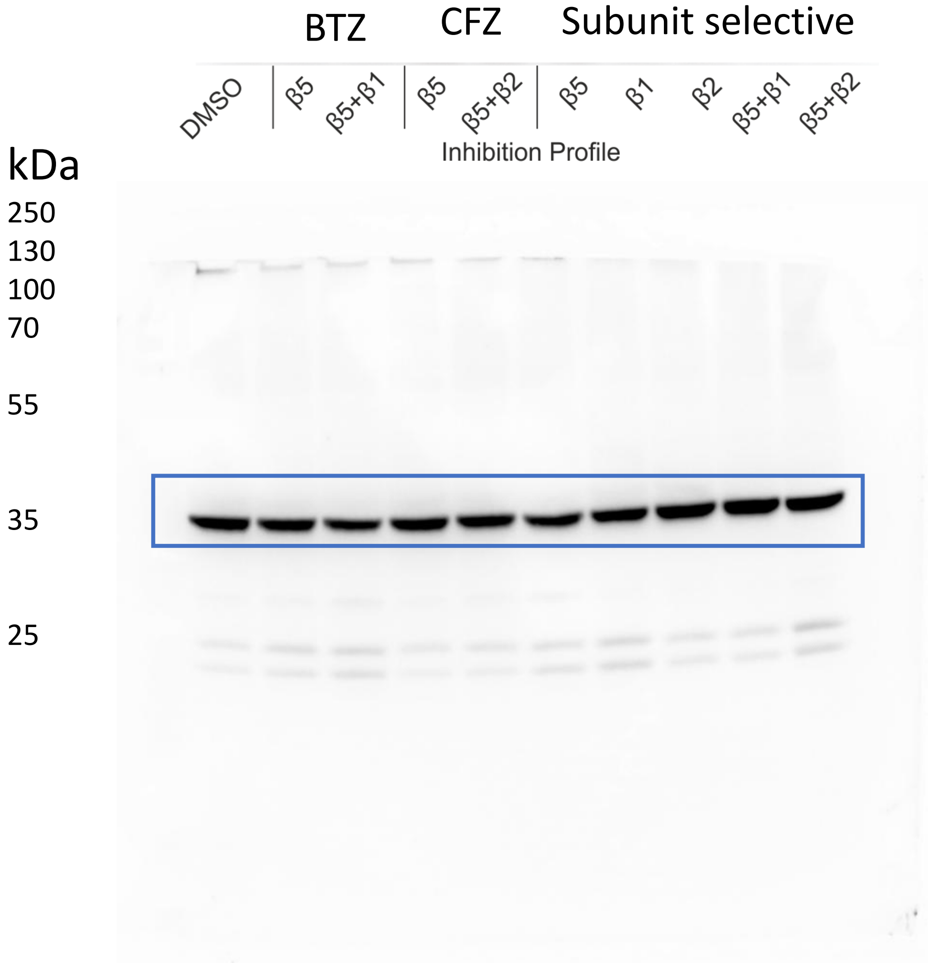

Figure 5A

CaMKII (pThr386): 50 kDa

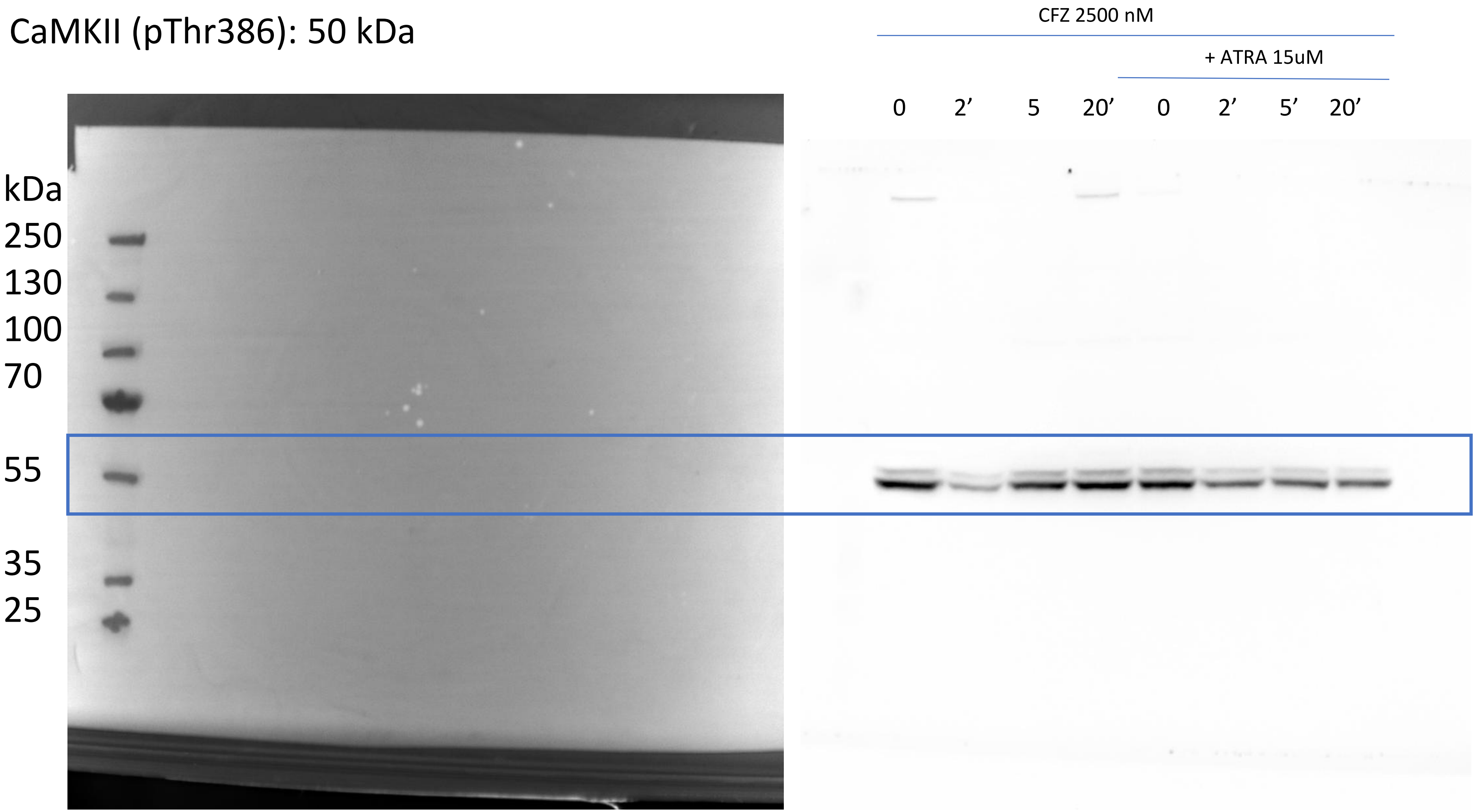

Figure 5A

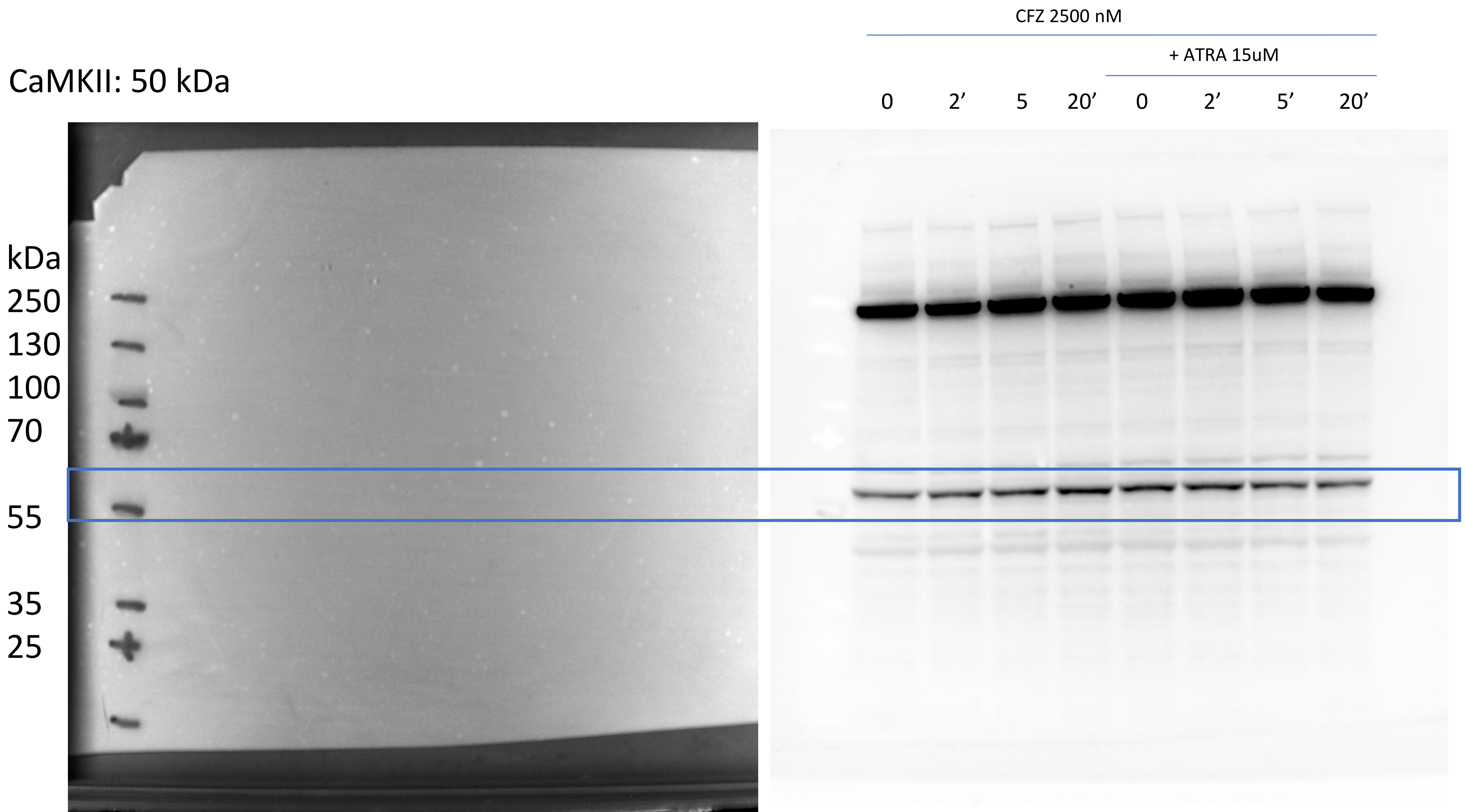
CFZ 2500 nM
+ ATRA 15uM
CaMKII: 50 kDa
0
2’
5
20’
0
2’
5’
20’
kDa
250
130
100
70
55
35
25

Figure 5A

CREB (pSer133): 43 kDa

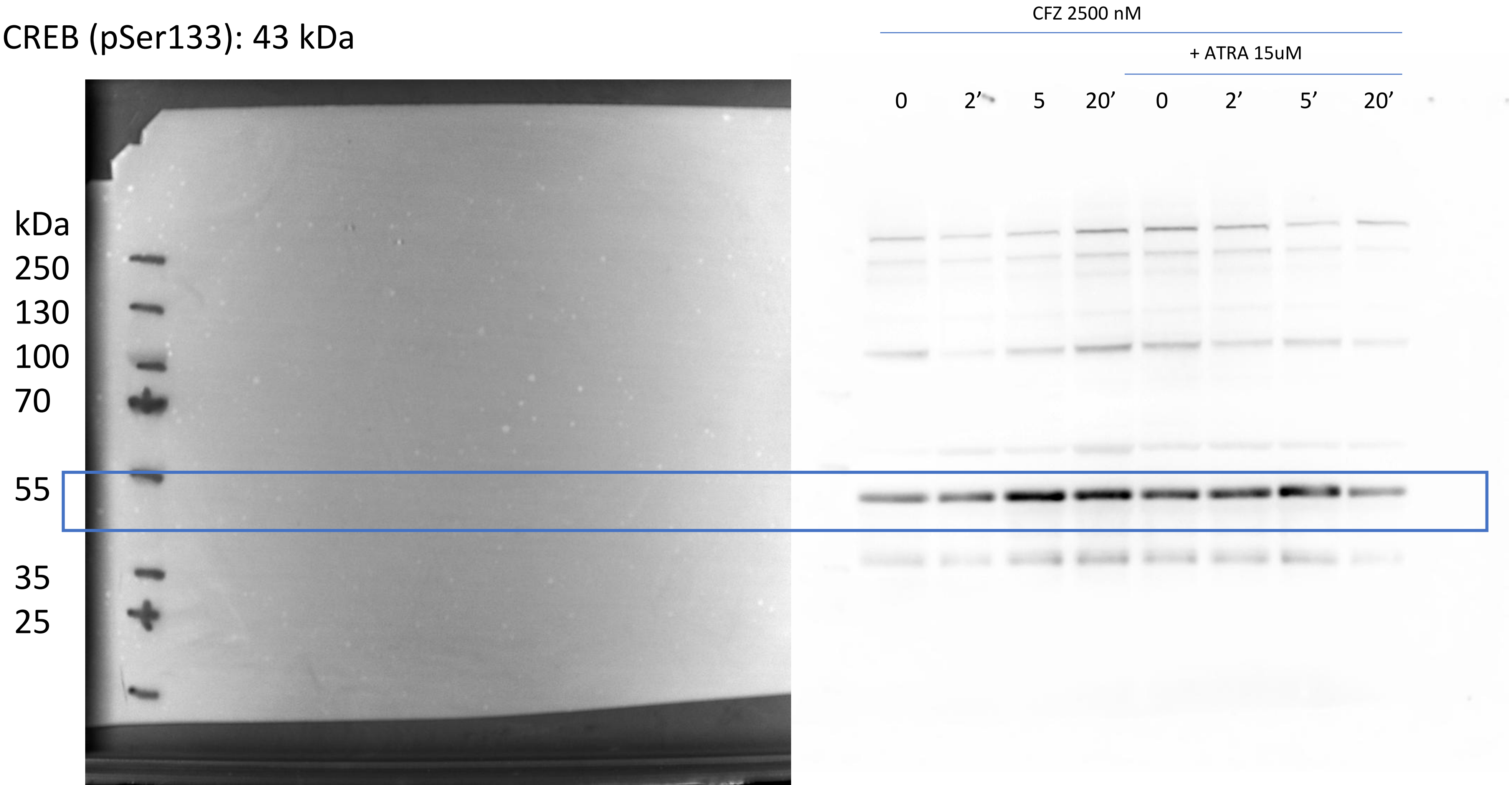

Figure 5A

CREB: 43 kDa

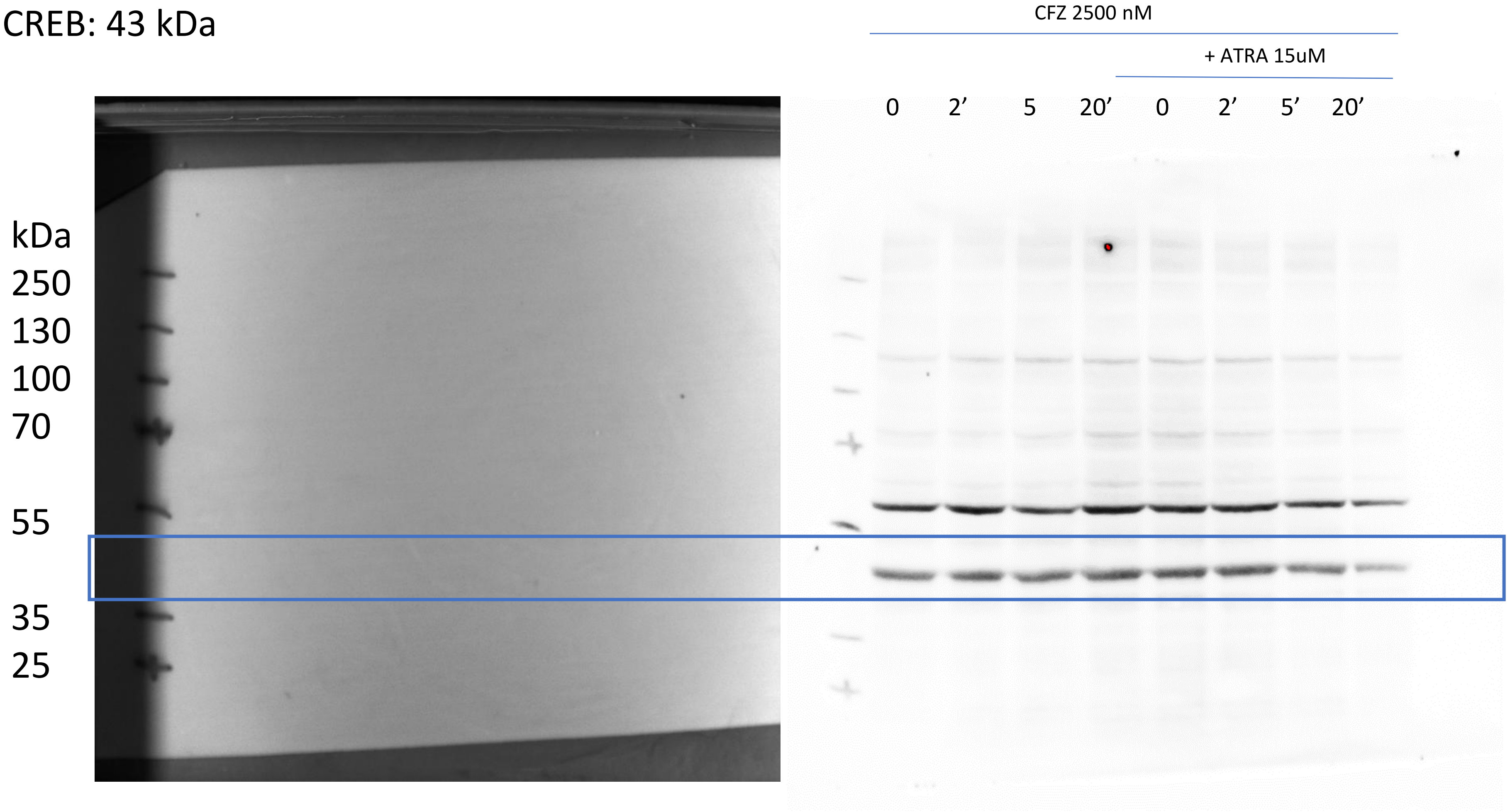

Figure 5A

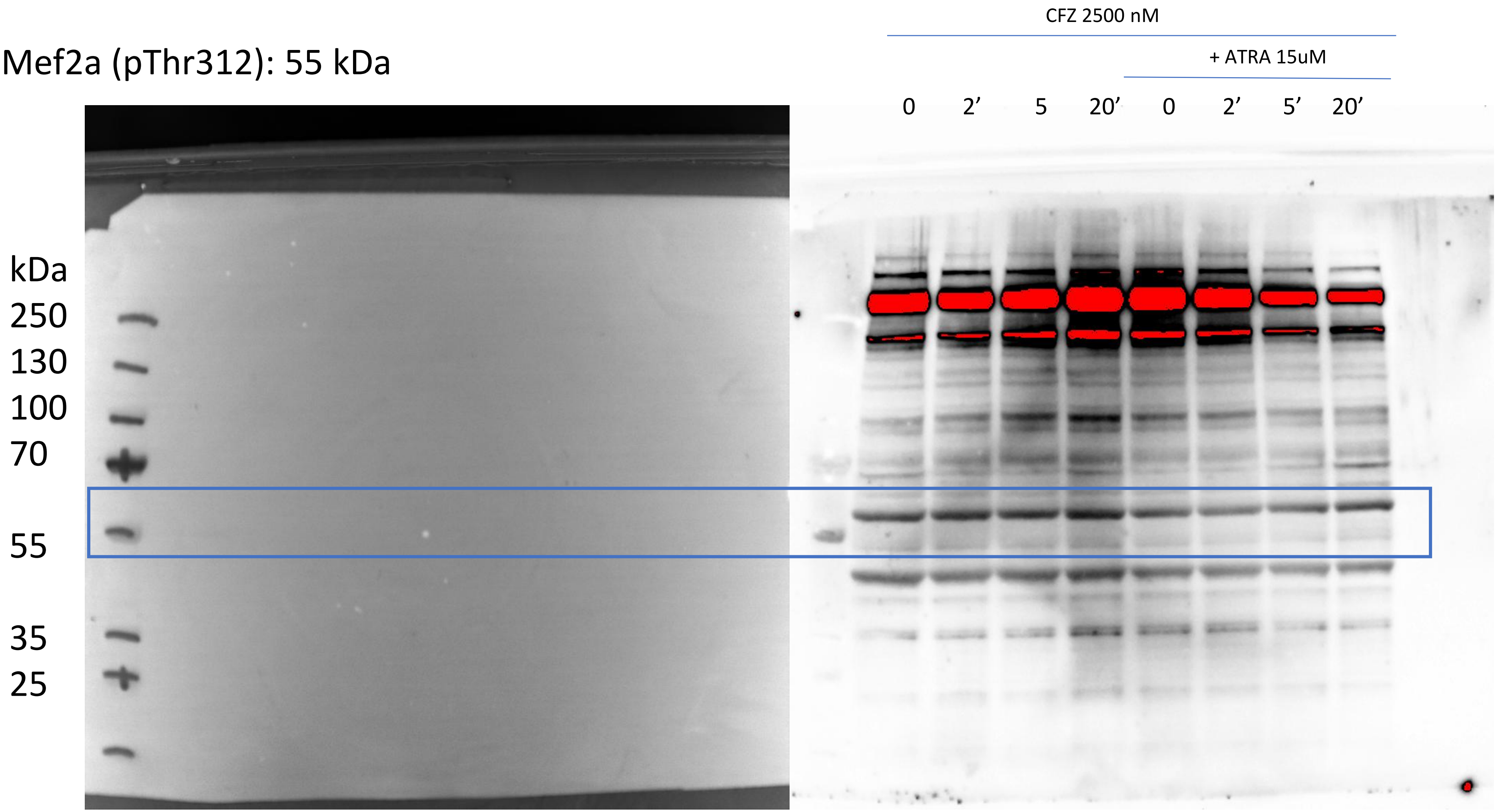

CFZ 2500 nM
+ ATRA 15uM
0 2' 5 20' 0 2' 5' 20'
Mef2a (pThr312): 55 kDa
kDa
250
130
100
70
55
35
25

Figure 5A

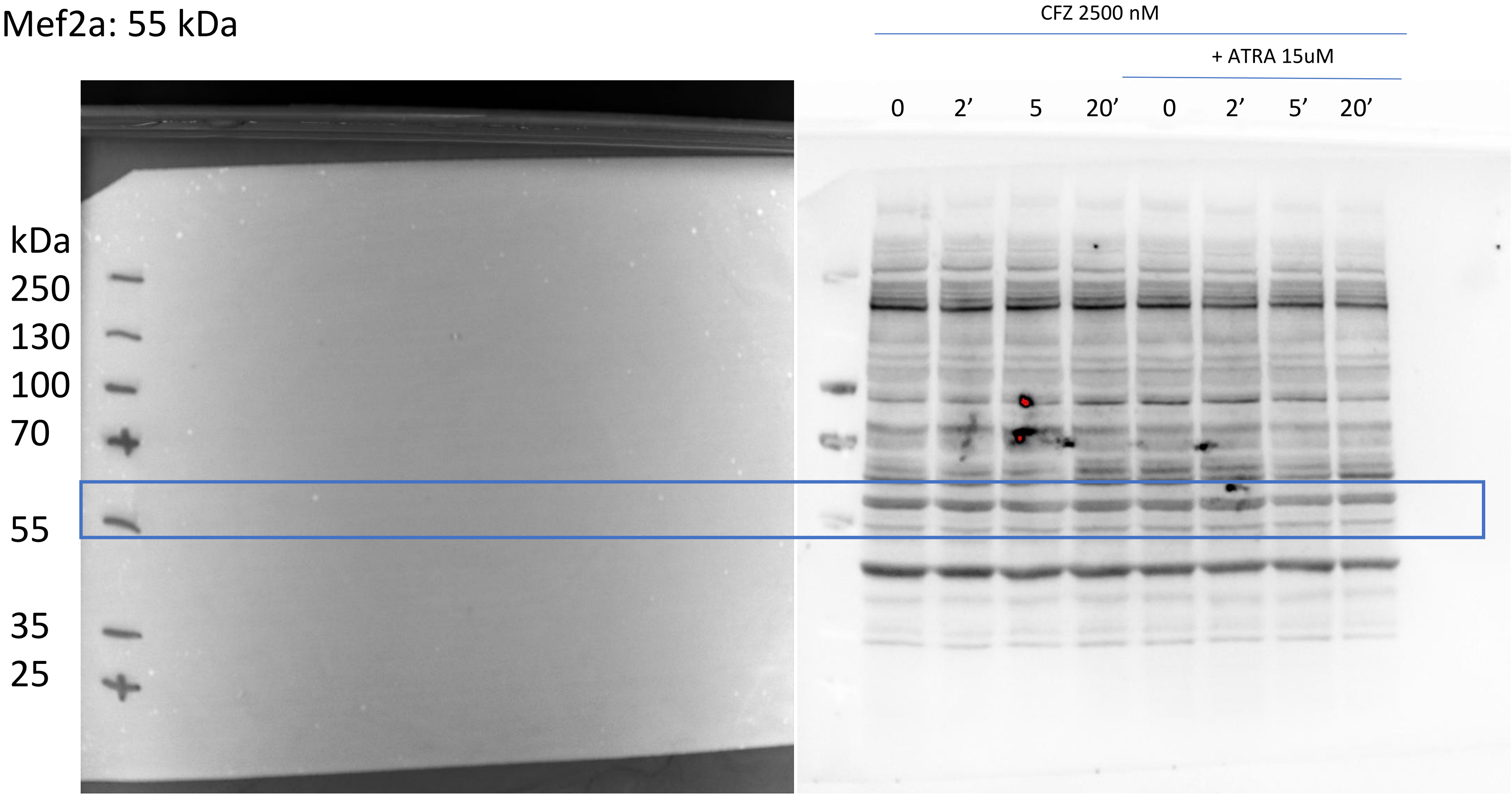

Mef2a: 55 kDa
CFZ 2500 nM
+ ATRA 15uM
0
2'
5
20'
0
2'
5'
20'
kDa
250
130
100
70
55
35
25

Figure 5A

Alpha-tubulin: 50 kDa

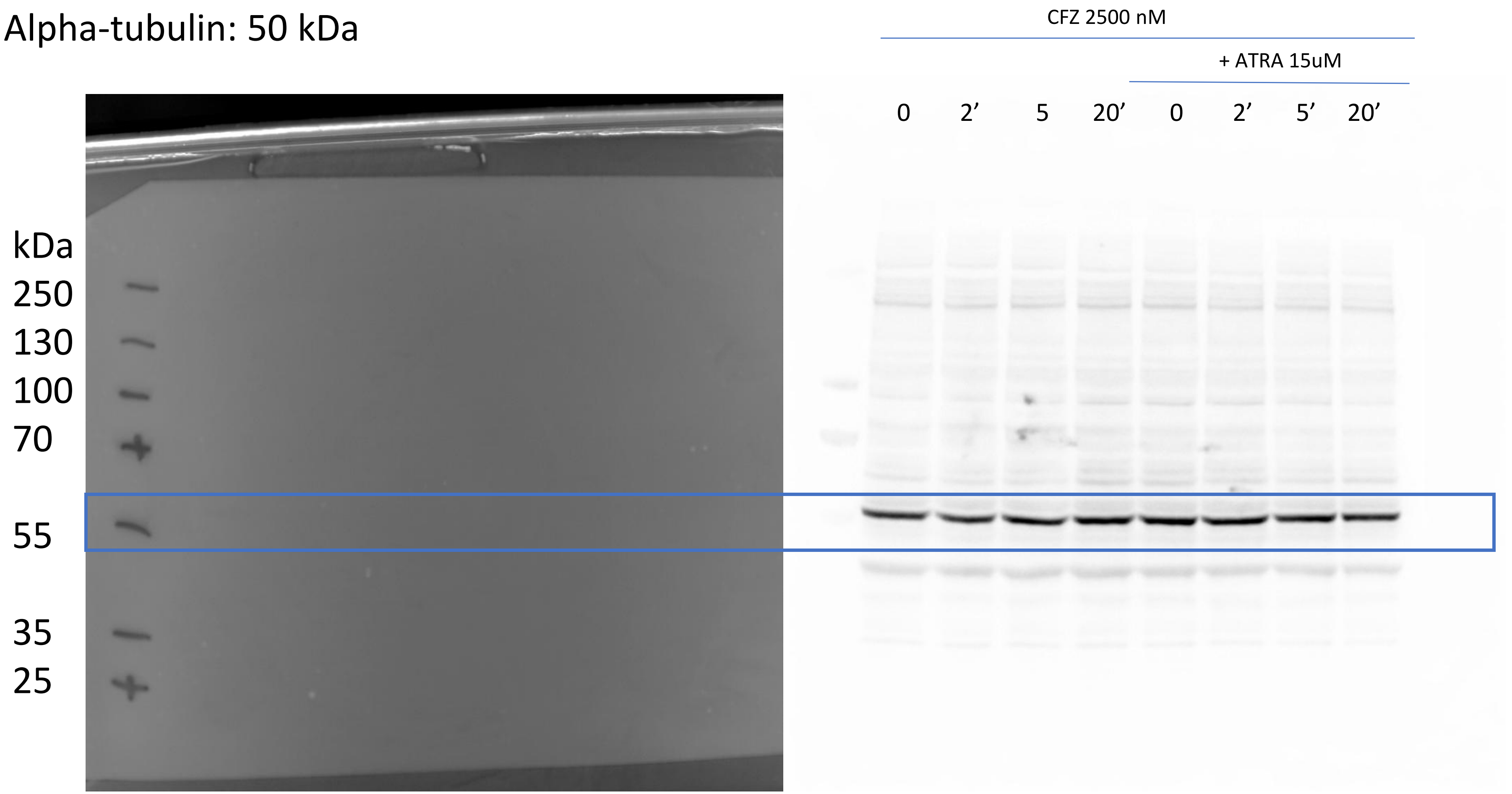

Figure 5C

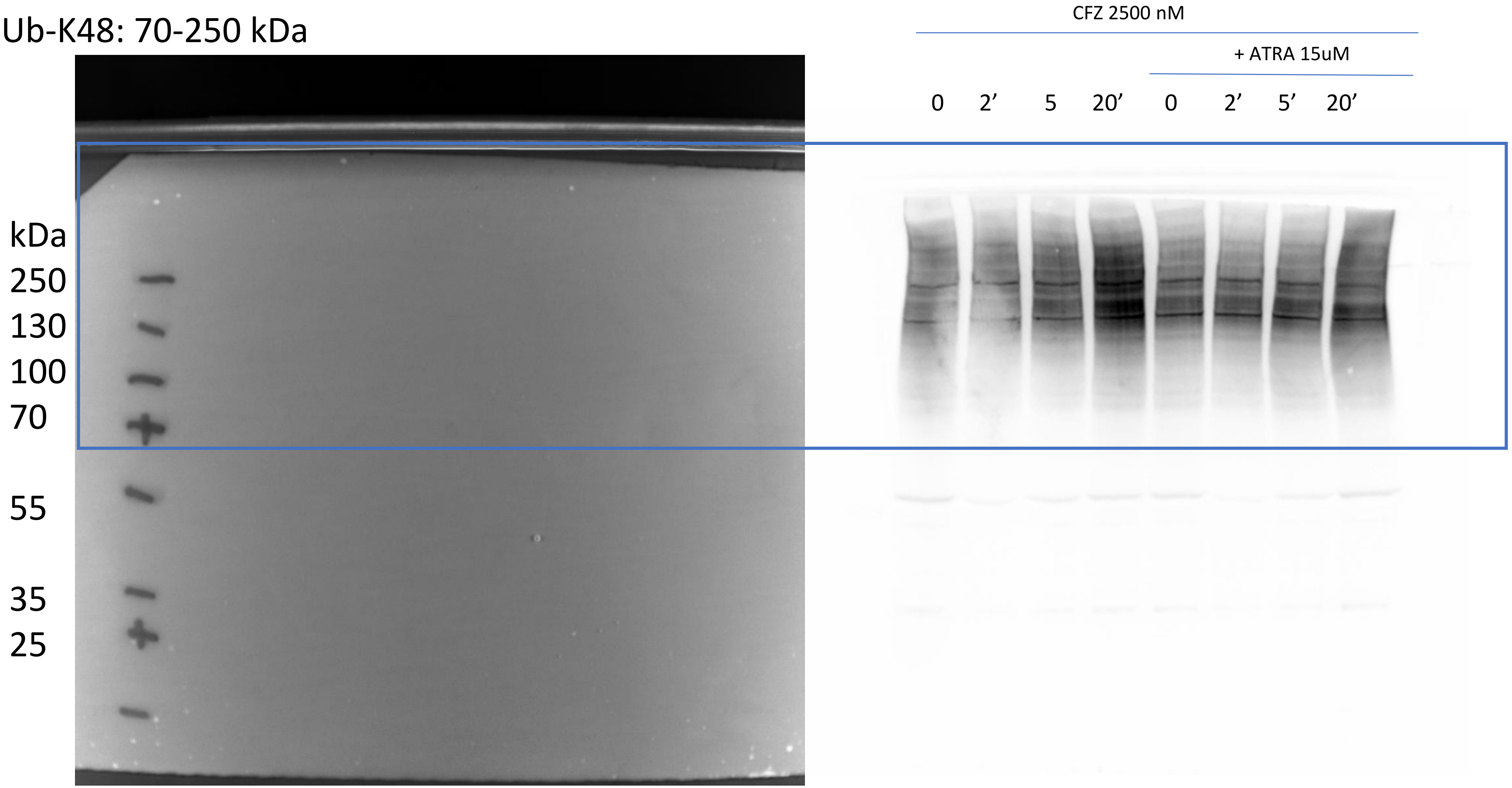
Ub-K48: 70-250 kDa
CFZ 2500 nM
+ ATRA 15uM
0
2’
5
20’
0
2’
5’
20’
kDa
250
130
100
70
55
35
25

Figure 5C

GAPDH: 37 kDa

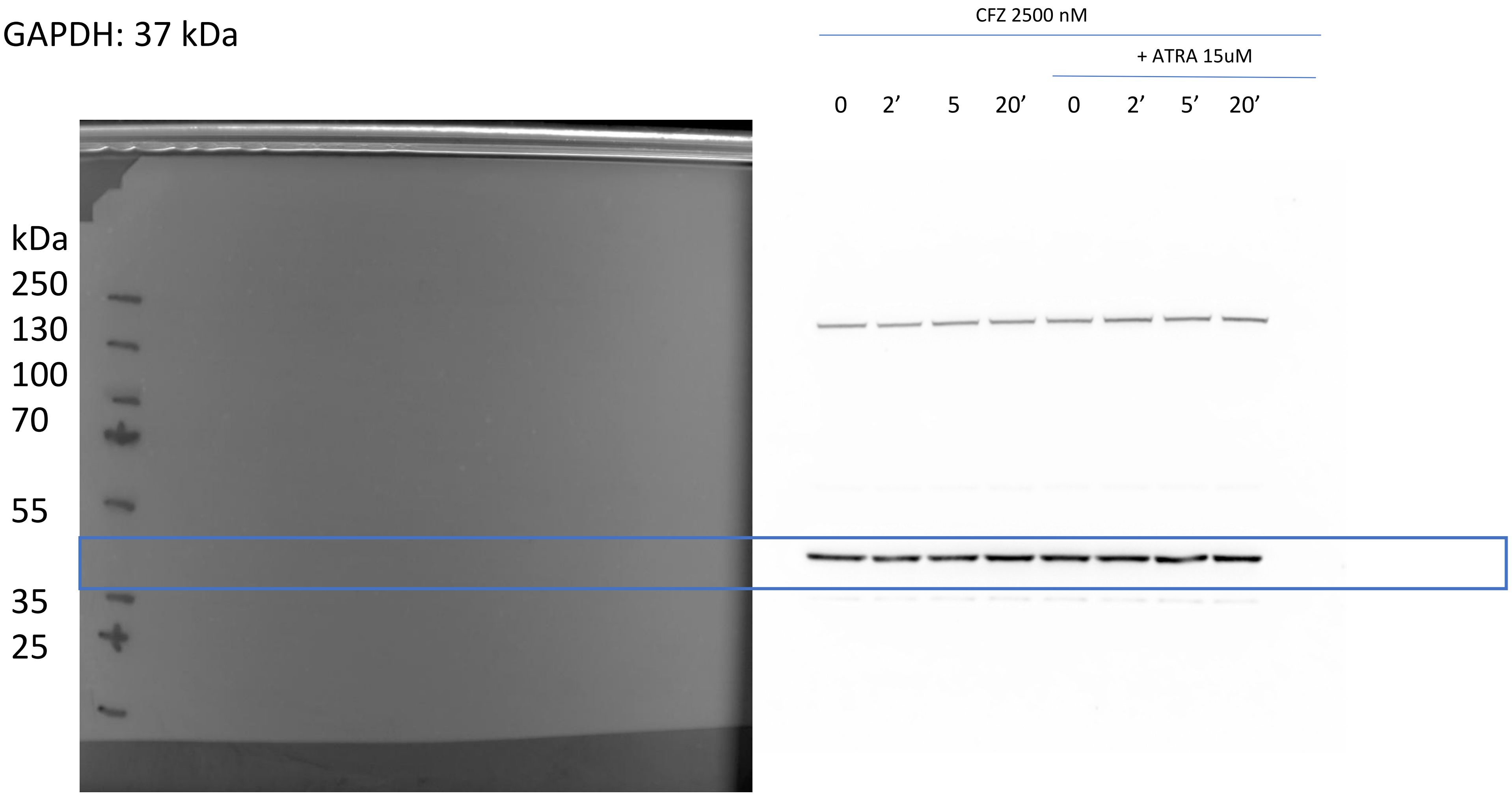

Figure 5C

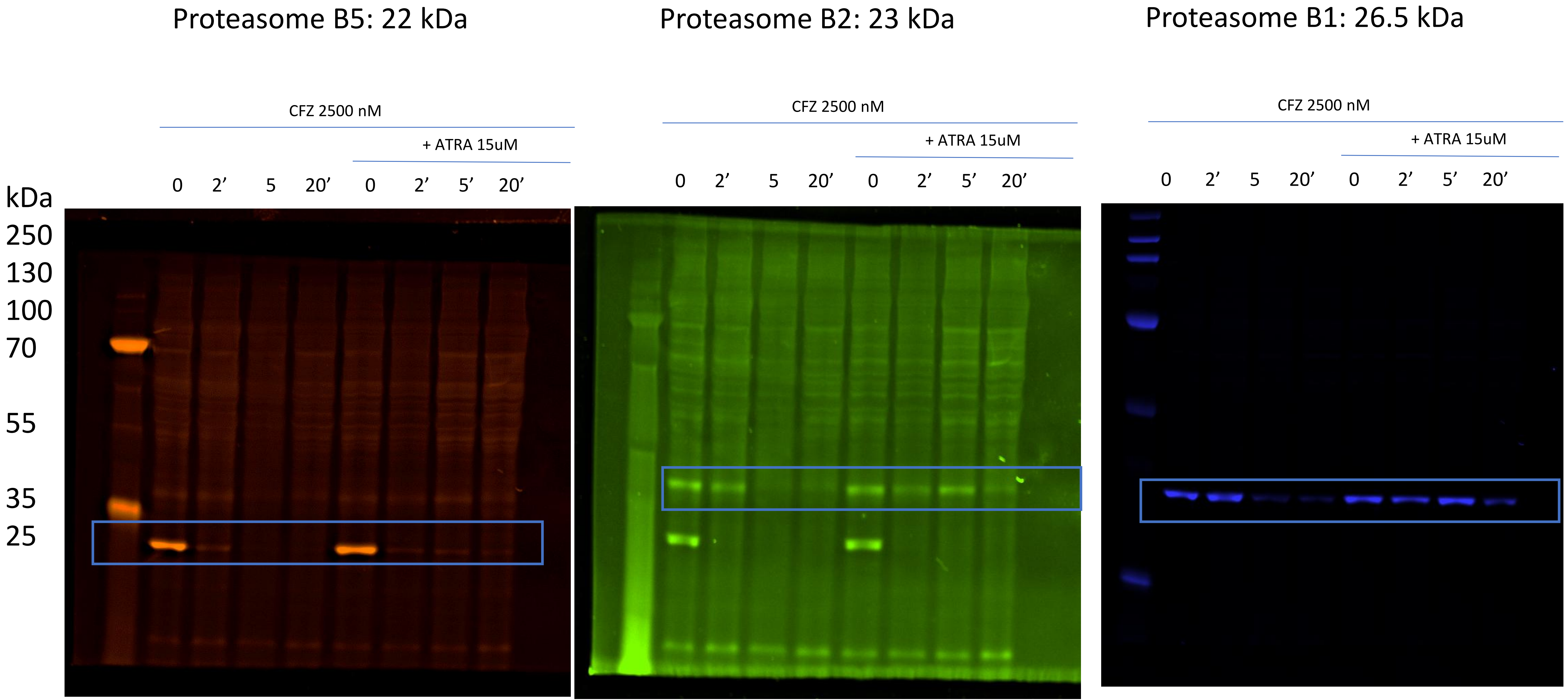
Proteasome B5: 22 kDa
Proteasome B2: 23 kDa
Proteasome B1: 26.5 kDa
CFZ 2500 nM
+ ATRA 15uM
0 2’ 5 20’ 0 2’ 5’ 20’
kDa
250
130
100
70
55
35
25

Figure S1A

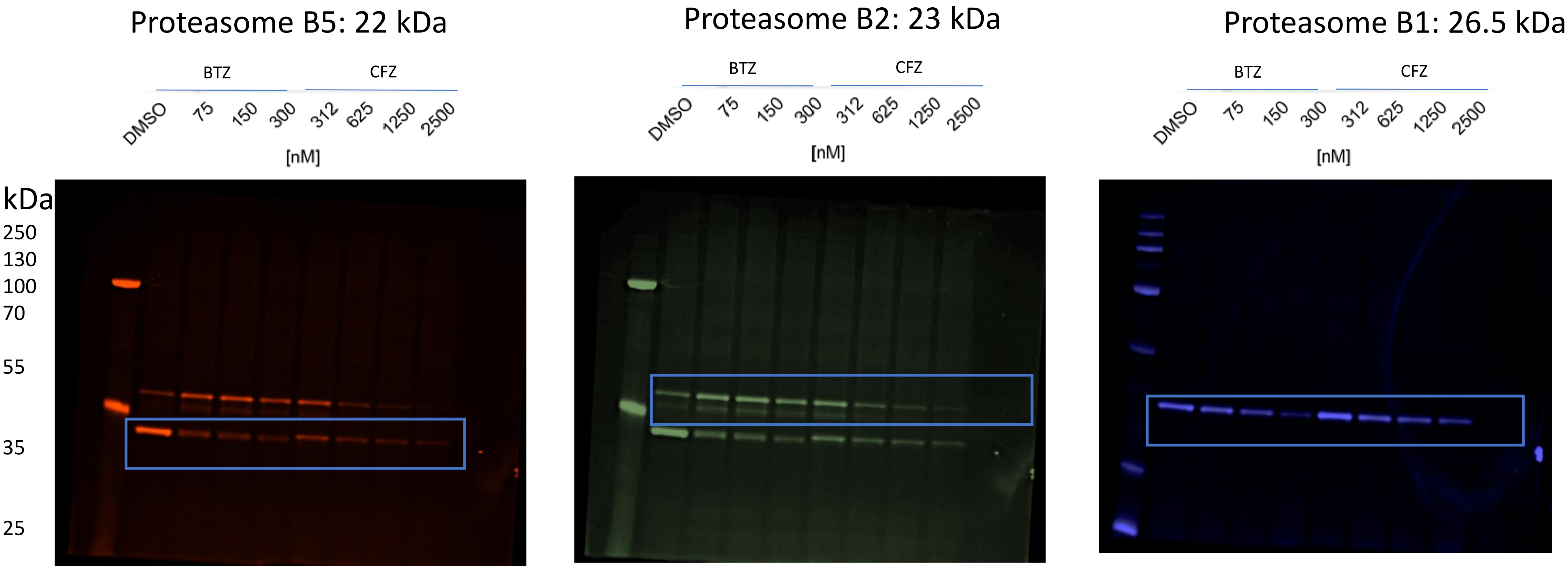
Proteasome B5: 22 kDa
Proteasome B2: 23 kDa
Proteasome B1: 26.5 kDa
BTZ
CFZ
DMSO
75
150
300
312
625
1250
2500
[nM]
kDa
250
130
100
70
55
35
25

Figure S1A

GAPDH: 37 kDa

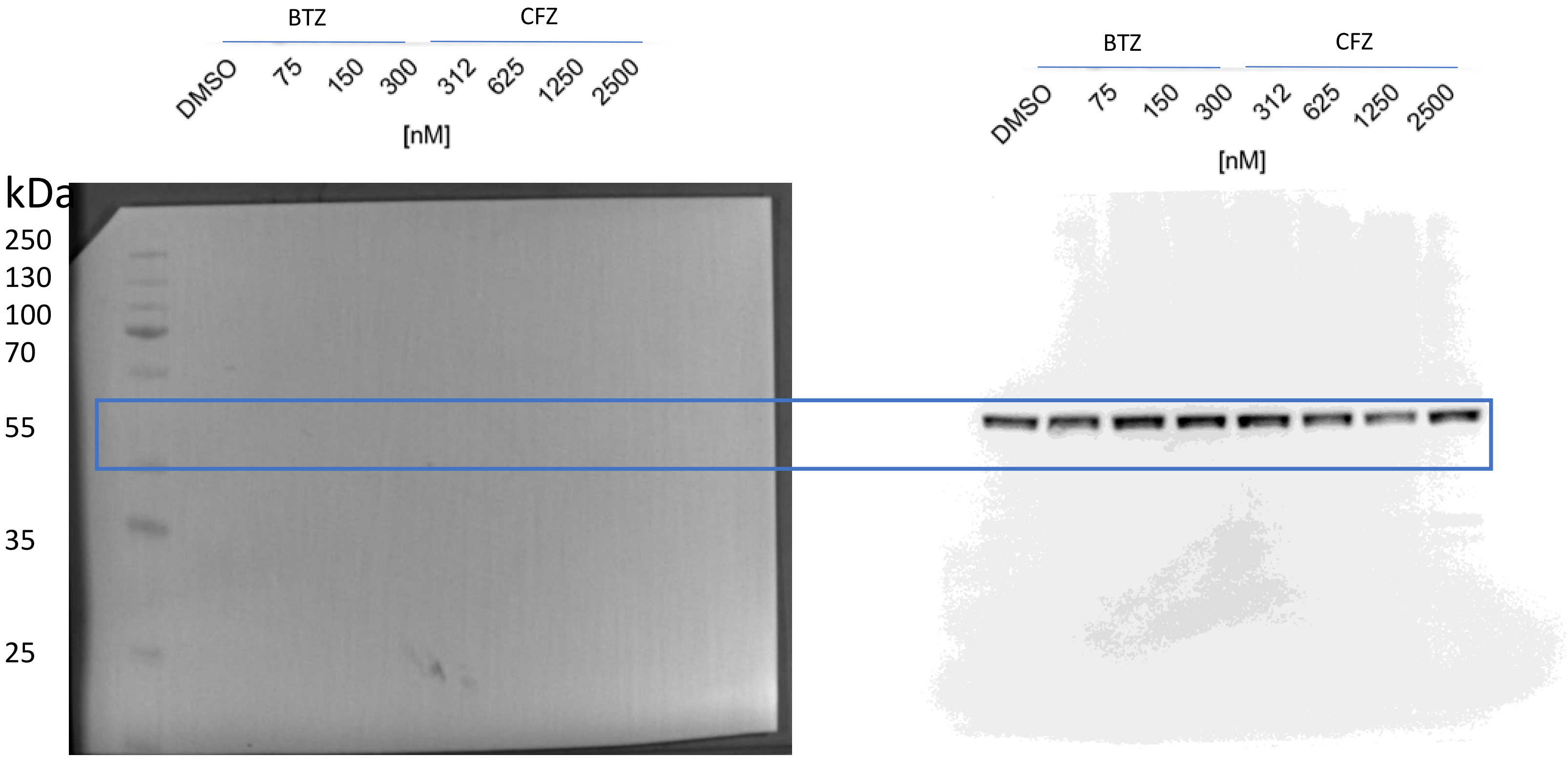

Figure S2E

X – indicates treatment with LU-102, a proteasome B2 inhibitor, which was not used in current study.

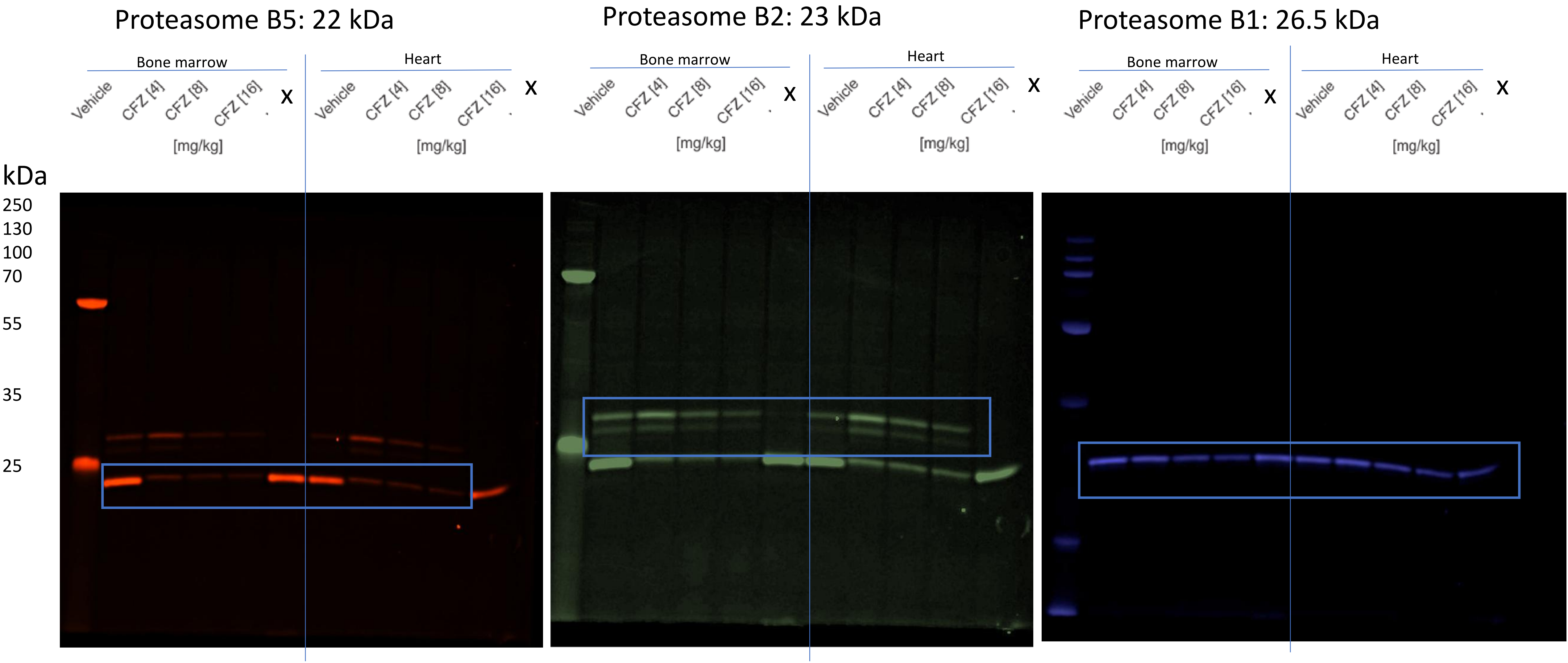

Figure S2E

X – indicates treatment with LU-102, a proteasome B2 inhibitor, which was not used in current study.

GAPDH: 37 kDa

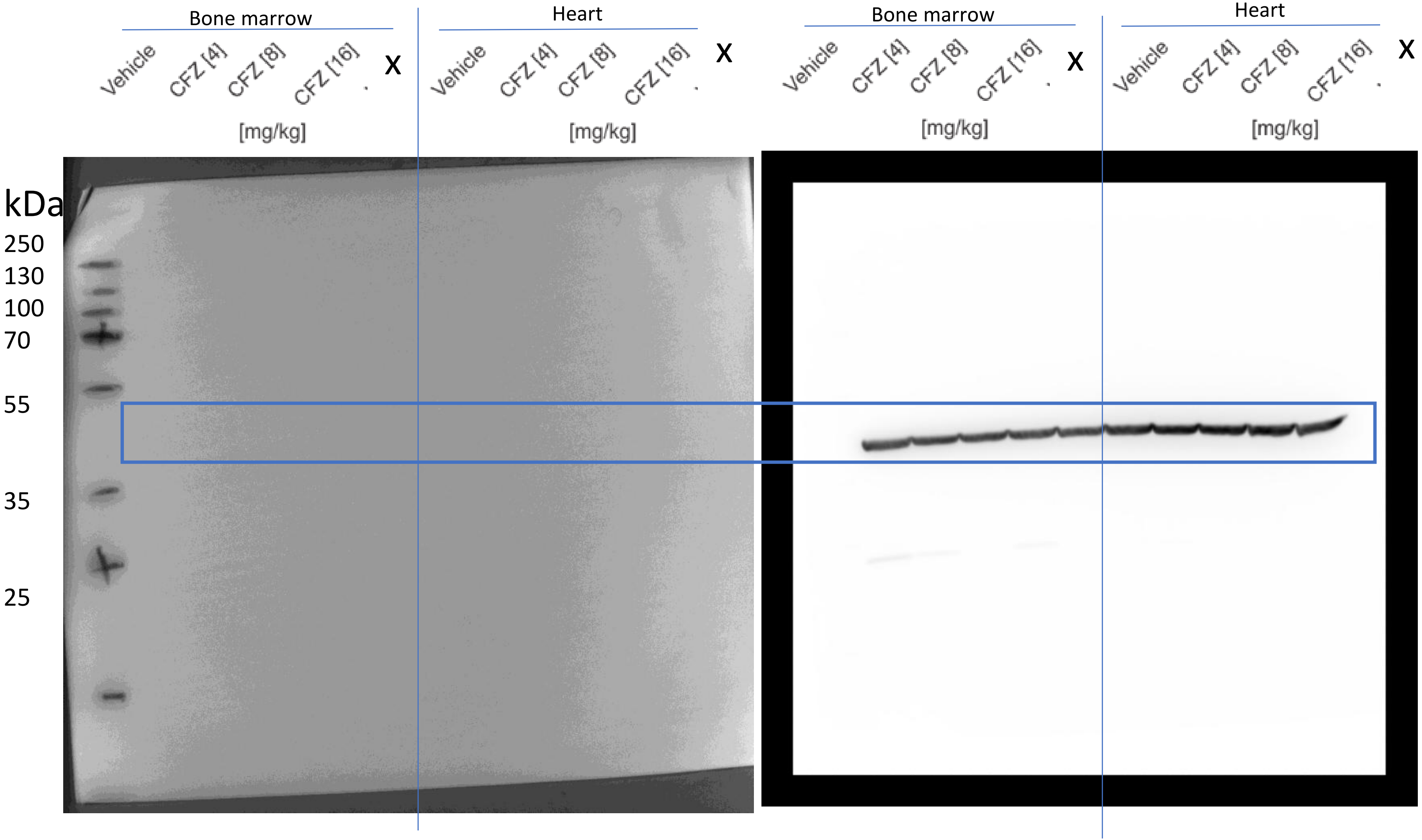

Figure S2F

X – indicates treatment with CFZ (16 mg/kg), but for clarity, only one representative animal was used.

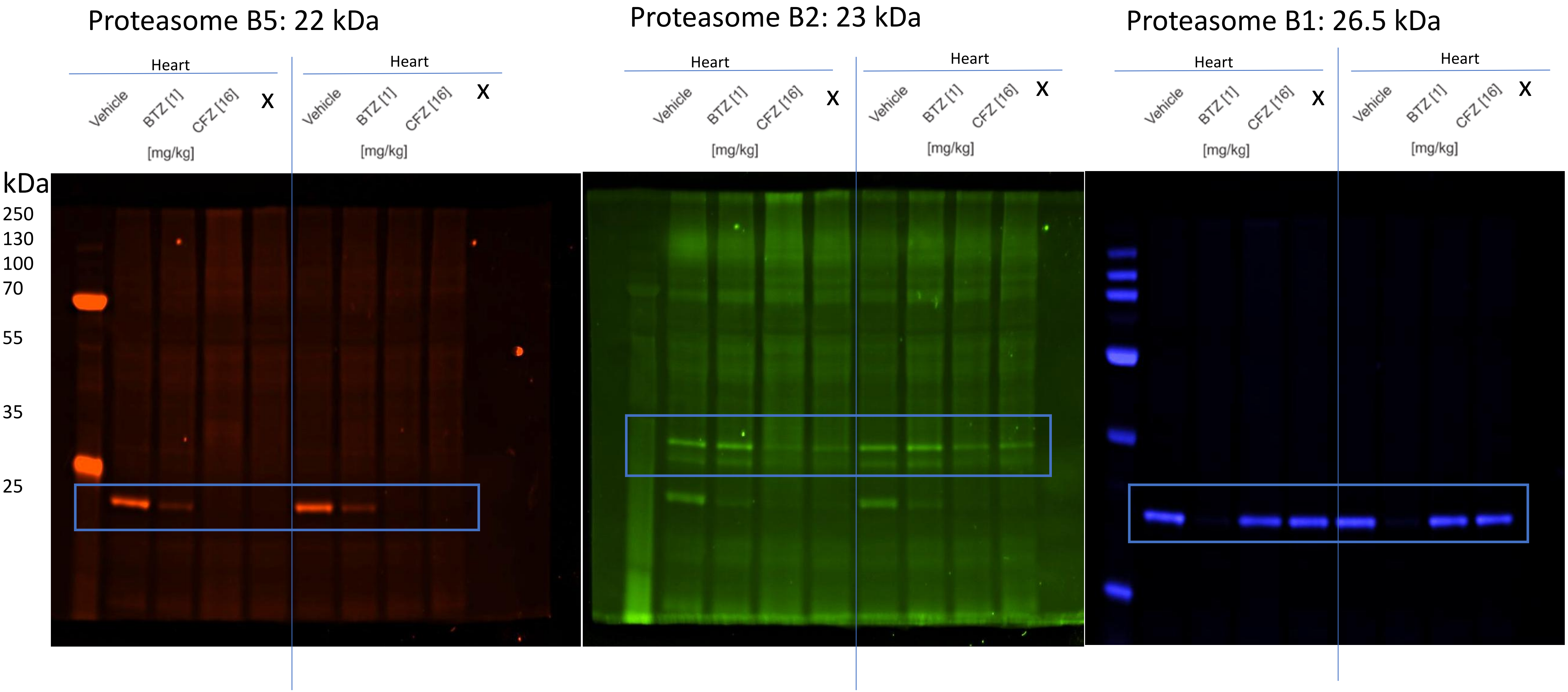

Figure S2F

X – indicates treatment with CFZ (16 mg/kg), but for clarity, only one representative animal was used.

GAPDH: 37 kDa

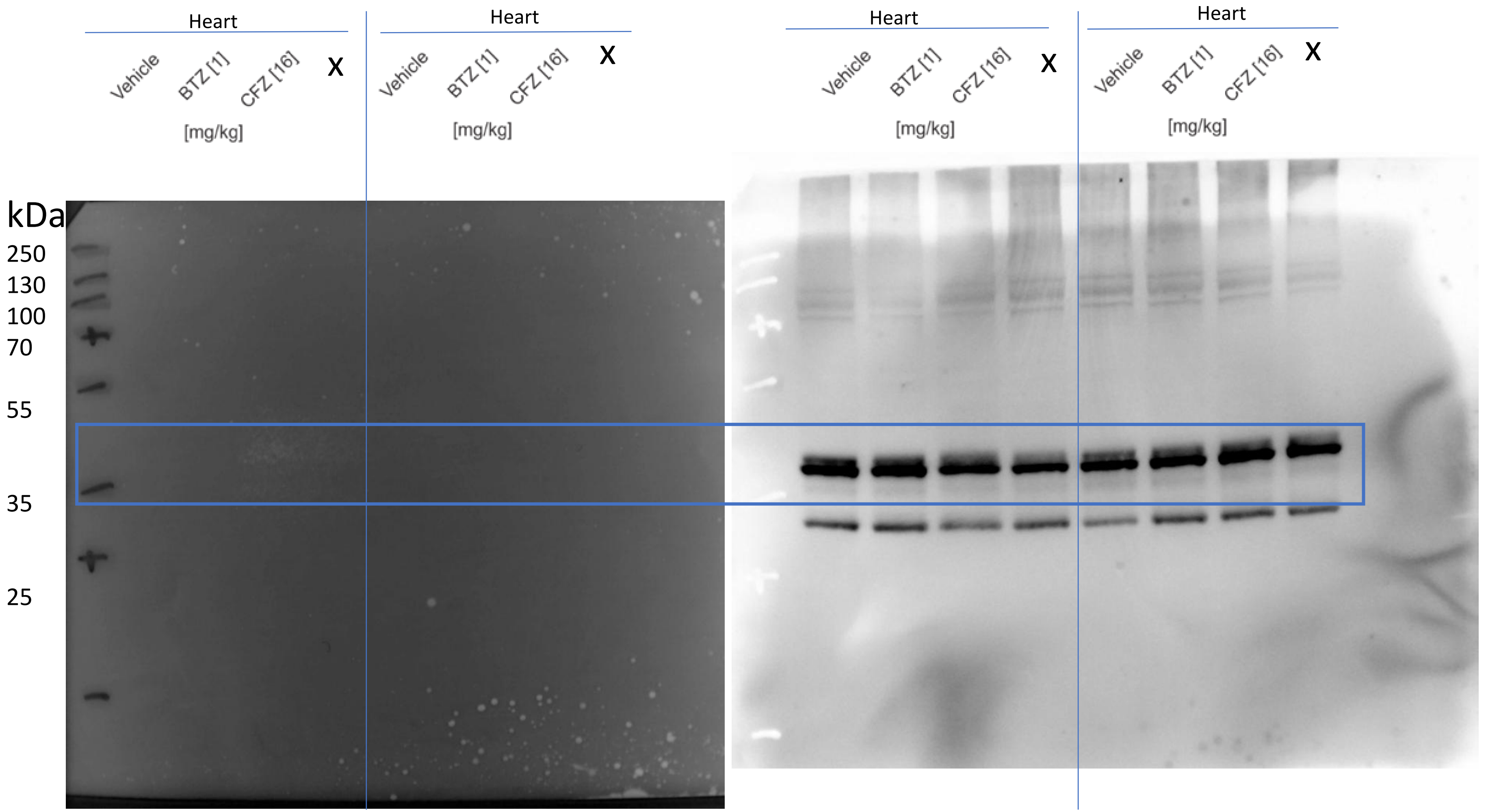

Figure S3F

X – indicates treatment with BTZ, which was not used in this respective Figure.

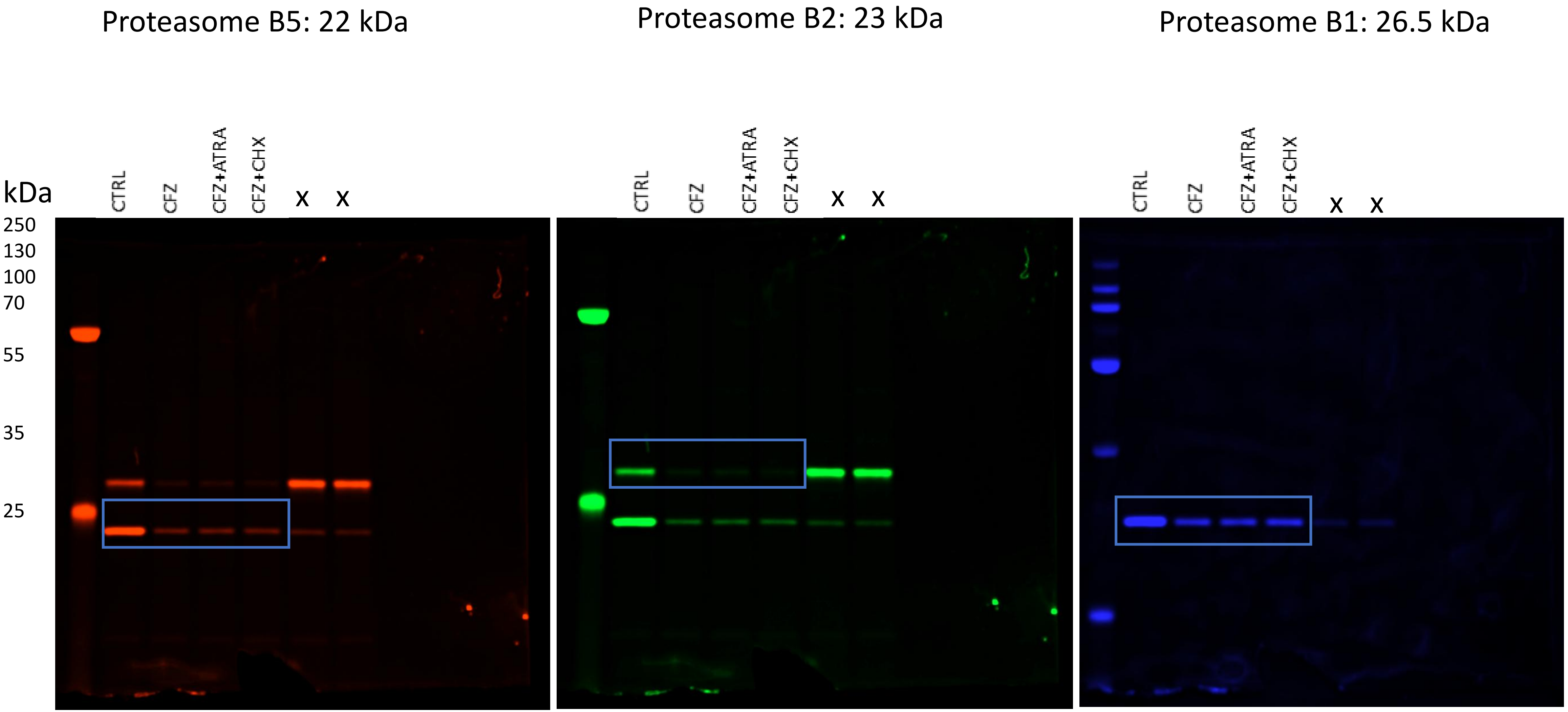

Figure S3F

X – indicates treatment with BTZ, which was not used in this respective Figure.

ALDH1A1: 55kDa, dimer 110 kDa

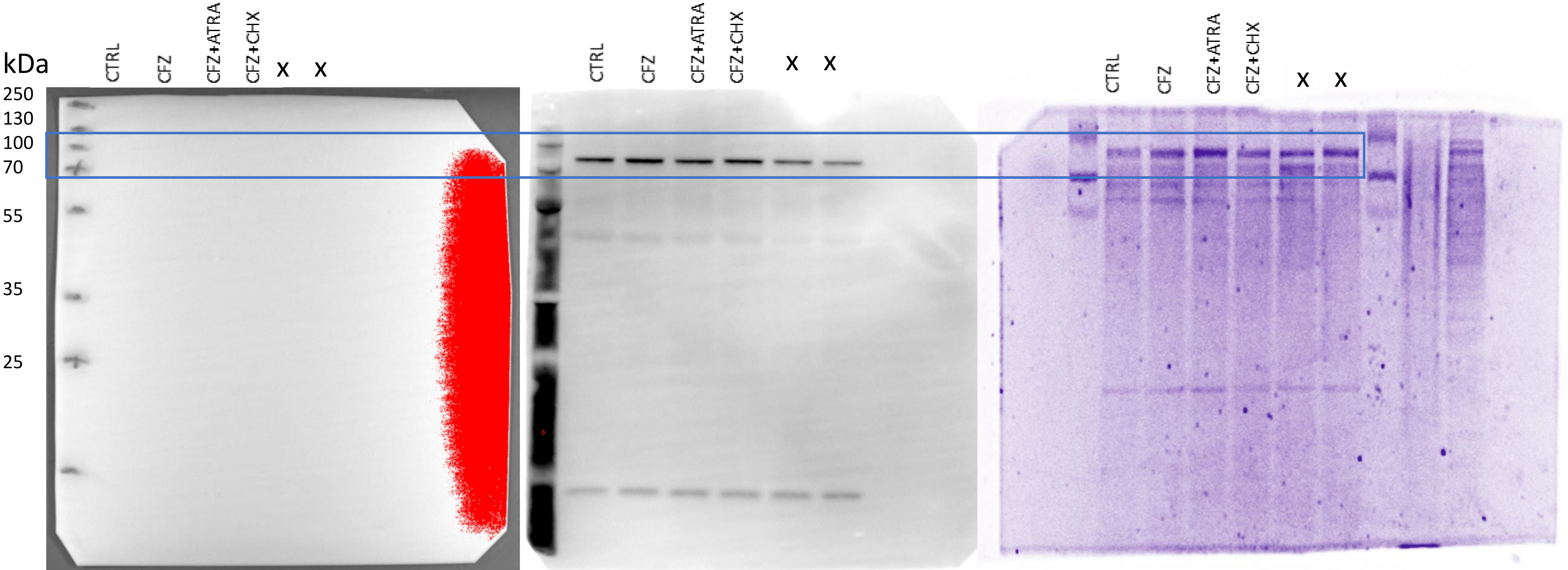

Figure S4B

Proteasome B5: 22 kDa

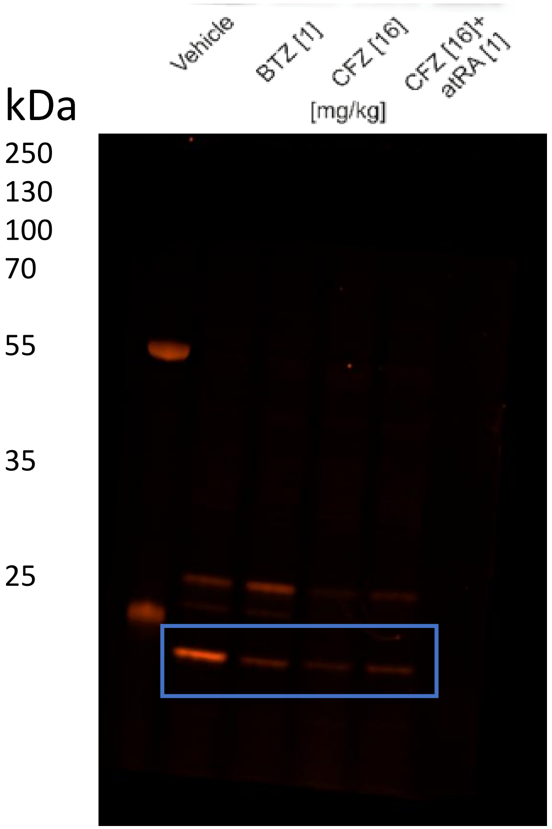

Proteasome B2: 23 kDa

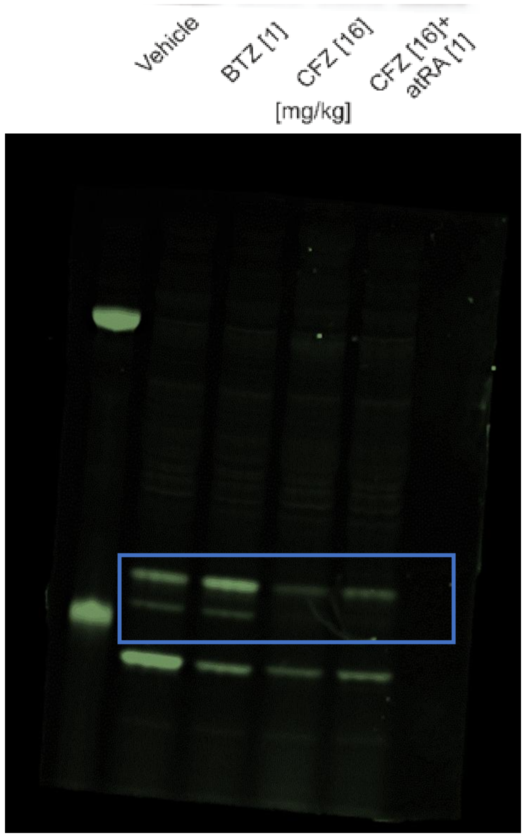

Proteasome B1: 26.5 kDa

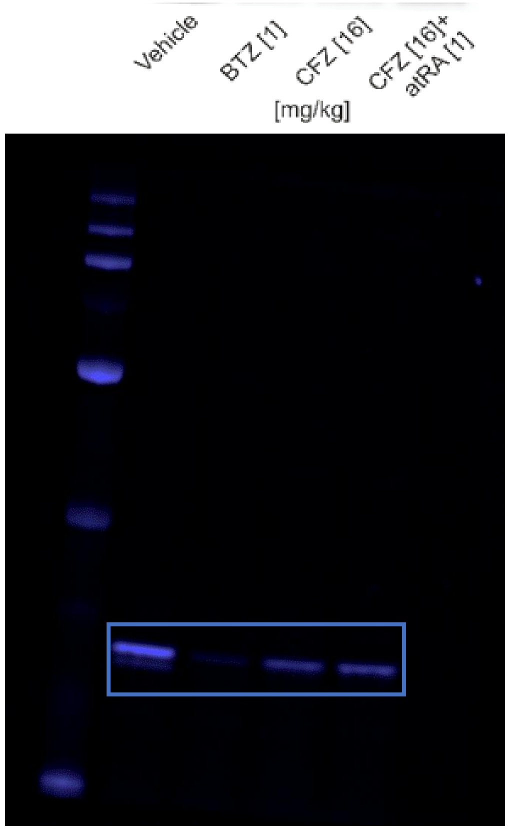

Figure S4B

GAPDH: 37 kDa

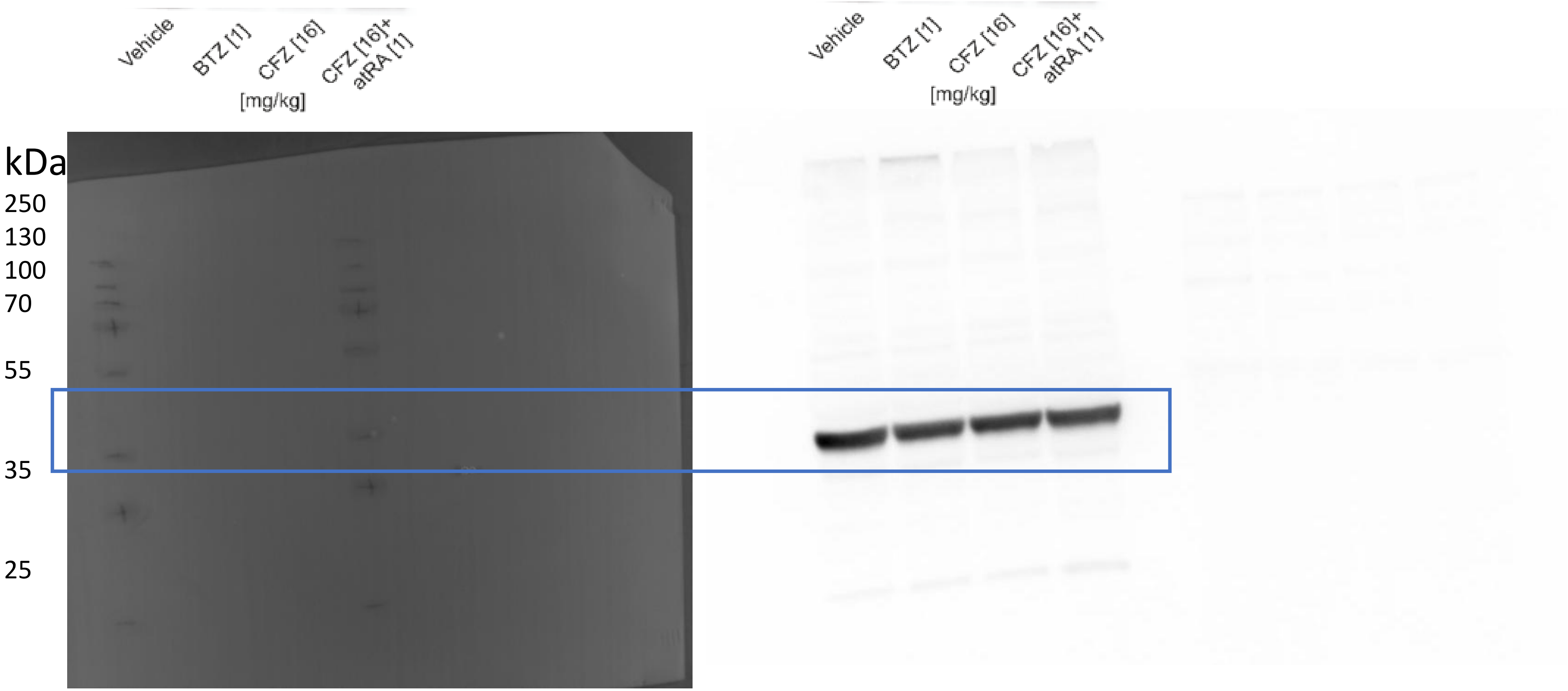

Supplement: Document S2. Original western blot data [file mmc2.pdf]
